# Combinatorial Biosynthesis Creates a Novel Aglycone Polyether with High Potency and Low Side Effects Against Bladder Cancer

**DOI:** 10.1002/advs.202404668

**Published:** 2024-06-27

**Authors:** Pan Yan, Gang Wang, Minjian Huang, Zhen Liu, Chong Dai, Ben Hu, Meijia Gu, Zixin Deng, Ran Liu, Xinghuan Wang, Tiangang Liu

**Affiliations:** ^1^ Key Laboratory of Combinatorial Biosynthesis and Drug Discovery Ministry of Education and School of Pharmaceutical Sciences Wuhan University Wuhan 430071 China; ^2^ Department of Urology Zhongnan Hospital of Wuhan University Wuhan 430071 China; ^3^ Department of Biological Repositories Human Genetic Resource Preservation Center of Hubei Province Zhongnan Hospital of Wuhan University Wuhan 430071 China; ^4^ Medical Research Institute Frontier Science Center of Immunology and Metabolism Wuhan University Wuhan 430071 China; ^5^ Wuhan Hesheng Technology Co., Ltd Wuhan 430074 China; ^6^ Precision Cancer Diagnostic Center Zhongnan Hospital of Wuhan University Wuhan 430071 China; ^7^ State Key Laboratory of Microbial Metabolism School of Life Sciences and Biotechnology Shanghai Jiao Tong University Shanghai 200240 China; ^8^ Key Laboratory of Quantitative Synthetic Biology Shenzhen Institute of Synthetic Biology Shenzhen Institutes of Advanced Technology Chinese Academy of Sciences Shenzhen 518055 China; ^9^ Department of Urology Zhongnan Hospital of Wuhan University School of Pharmaceutical Sciences Wuhan University Wuhan 430071 China

**Keywords:** aglycone polyether, anti‐bladder cancer agents, combinatorial biosynthesis, drug design, structure‐activity relationships

## Abstract

Polyethers play a crucial role in the development of anticancer drugs. To enhance the anticancer efficacy and reduce the toxicity of these compounds, thereby advancing their application in cancer treatment, herein, guided by the structure‐activity relationships of aglycone polyethers, novel aglycone polyethers are rationally redesigned with potentially improved efficacy and reduced toxicity against tumors. To realize the biosynthesis of the novel aglycone polyethers, the gene clusters and the post‐polyketide synthase tailoring pathways for aglycone polyethers endusamycin and lenoremycin are identified and subjected to combinatorial biosynthesis studies, resulting in the creation of a novel aglycone polyether termed End‐16, which demonstrates significant potential for treating bladder cancer (BLCA). End‐16 demonstrates the ability to suppress the proliferation, migration, invasion, and cellular protrusions formation of BLCA cells, as well as induce cell cycle arrest in the G1 phase in vitro. Notably, End‐16 exhibits superior inhibitory activity and fewer side effects against BLCA compared to the frontline anti‐BLCA drug cisplatin in vivo, thereby warranting further preclinical studies. This study highlights the significant potential of integrating combinatorial biosynthesis strategies with rational design to create unnatural products with enhanced pharmacological properties.

## Graphical abstract

Based on the structure‐activity relationship of aglycone polyethers and the biosynthetic mechanisms of lenoremycin and endusamycin, new biosynthetic pathways are rationally designed, resulting in the creation of a novel C11‐glycosylated polyether, End‐16. In mouse models, End‐16 exhibits superior inhibitory activity and fewer side effects against bladder cancer compared to the frontline clinical drug cisplatin.

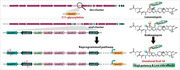

## 1 Introduction

Polyethers, a subclass of polyketides, represent an important class of secondary metabolites in the field of biomedicine. Notably, many polyethers have been reported to exhibit remarkable anticancer properties. Halichondrin B, sourced from the sponge *Halichondria*, demonstrated potent antitumor activity and served as the foundation for the development of eribulin, a drug used to treat metastatic breast cancer.^[^ ^1^ ^]^ In addition, *Streptomyces*‐derived salinomycin has exhibited potent inhibitory activity against various tumor stem cells, particularly breast cancer stem cells, sparking widespread interest in the field of polyethers anticancer research.^[^ ^2^ ^]^ Moreover, in our previous studies, we observed significant inhibitory effects of aglycone polyethers nanchangmycin and its analogues, containing endusamycin (End), lenoremycin (Len), and CP‐80,219, on 39 different types of cancer cells, with enhanced efficacy against breast cancer stem cells in comparison to salinomycin.^[^ ^3^ ^]^ Subsequent work by Ikeda et al., also revealed that Len could selectively inhibit the proliferation of colon cancer stem cells.^[^ ^4^ ^]^ These findings highlight the significant potential of aglycone polyethers as effective anticancer agents.

Bladder cancer (BLCA) comprises one of the most common tumors of the genitourinary system, with an estimated 570 000 new cases and 210 000 deaths worldwide each year.^[^ ^5^ ^]^ BLCA is usually clinically classified as non‐muscle invasive bladder cancer (NMIBC) and muscle invasive bladder cancer (MIBC). Currently, the primary treatment for patients with NMIBC involves transurethral resection of bladder tumor, along with intravesical therapy. However, treated patients with NMIBC often experience high recurrence rates, with some progressing to MIBC. The current standard treatment for patients with MIBC involves radical cystectomy, which is invasive and severely affects the patients' quality of life.^[^ ^6^ ^]^ For advanced BLCA, the first‐line treatment remains cisplatin‐based combination chemotherapy, which often leads to severe side effects.^[^ ^7^ ^]^ In recent years, the Food and Drug Administration has approved several immune checkpoint inhibitors that target programmed cell death protein 1 and programmed cell death ligand 1 for the treatment of advanced or metastatic BLCA. However, the overall objective response rate of these inhibitors has been observed to be less than 24%.^[^ ^8^ ^]^ Thus, considering the practical challenges faced in clinical treatment, it is urgent to discover new effective drugs with low side effects that can be used in the treatment or adjuvant therapy of BLCA.

**Figure 1:**
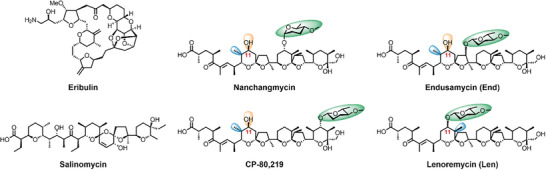
Reported representative polyethers with antiproliferative activity.

We previously found that aglycone polyethers nanchangmycin and its analogues (End, Len, and CP‐80,219) exhibit significant anticancer activity, with Len showing the highest potency followed by End, nanchangmycin, and CP‐80,219, albeit with nonnegligible toxicity associated with Len.^[^ ^3^ ^]^ These compounds share a similar backbone structure, and differ only in their modifying sugar, methyl, and hydroxyl groups (**Figure** **1**), indicating that the anticancer efficacy and toxicity of these drugs are closely correlated with the composition of these modifying groups. Notably, we observed that the anticancer potency of these compounds increases with the shortening of the distance between the sugar moiety and the carboxyl group, with the optimal site for sugar attachment being the C11 position.^[^ ^3^ ^]^ Furthermore, nanchangmycin, End, and CP‐80,219, all of which feature a hydroxyl group at the C11 position, have been shown to facilitate the attachment of additional sugar moieties, with End demonstrating the most prominent anticancer efficacy. This inspired us to explore C11‐glycosylated derivatives based on End and its analogues, with the objective of developing novel compounds with improved efficacy and reduced toxicity for the treatment of BLCA. However, achieving these structural modifications through chemical methods poses challenges due to the complex structure and stereochemistry of polyethers, as well as the susceptibility of polyether rings to cleavage. Alternatively, combinatorial biosynthesis strategies provide a promising approach. Given the presence of a sugar moiety at the C11 position in Len and its structural similarity to End, the biosynthesis of novel C11‐glycosylated derivatives is likely to be achieved through the rational design of new biosynthetic pathways that is based on the biosynthetic mechanisms of End and Len. However, these endeavors have been impeded by the absence of pertinent research on the biosynthetic gene clusters and biosynthetic mechanisms of End and Len.

In this study, to obtain novel C11‐glycosylated polyether derivatives, we first identified the biosynthetic gene cluster of End and successfully generated a series of End analogues harboring a hydroxyl group at the C11 position by employing gene disruption strategy. In addition, we identified the biosynthetic gene cluster and post‐polyketide synthase (PKS) tailoring pathway of Len, and successfully found the glycosyltransferase LenG5 capable of catalyzing C11‐glycosylation. The rational design of new biosynthetic pathways subsequently resulted in the creation of a novel C11‐glycosylated polyether, End‐16, which demonstrated superior inhibitory activity and fewer side effects against BLCA in comparison to the frontline clinical drug cisplatin, warranting further preclinical studies.

## 2 Results and Discussion

### 2.1 Generation of End Structural Analogues

**Figure 2:**
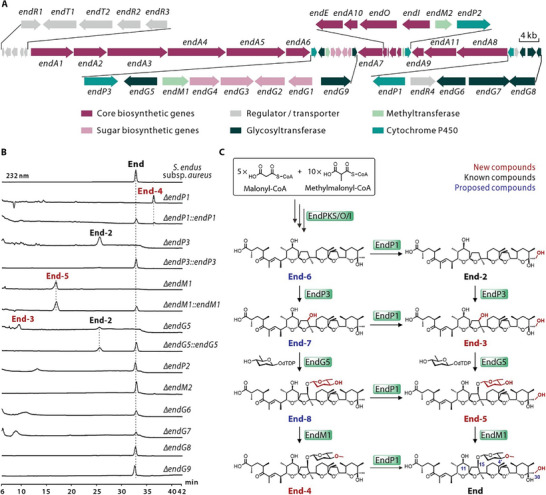
Generation of structural analogues of End. A) Genetic organization of the *end* cluster. B) HPLC profiles of extracts from the *S. endus* subsp. *aureus* wild‐type strain and related derivative strains. C) Proposed the tailoring steps in End biosynthesis. New compounds, known compounds and proposed compounds are indicated by red, black, and blue numbers, respectively.

Initially, to establish a diverse array of substrates for C11‐glycosylated derivatives, we sought to create a library of End structural analogues. A comprehensive understanding of the biosynthetic mechanism of End is crucial for carrying out structural modifications to obtain the analogues. However, the biosynthetic gene cluster of End has not yet been reported. To identify the biosynthetic gene cluster of End, we sequenced the complete genome of End‐producing *Streptomyces endus* subsp. *aureus*. Bioinformatics analyses revealed a type I PKS gene cluster with high similarity to the biosynthetic gene cluster of nanchangmycin.^[^ ^9^ ^]^ Given the structural similarities between these two compounds, this cluster, hereinafter termed the *end* cluster, consisting of 34 open reading frames (ORFs) was hypothesised to be responsible for the biosynthesis of End (**Figure** **2**A; Table S1, Supporting Information). In order to confirm this hypothesis, the entire *end* cluster was directly cloned using the ExoCET method and inserted into the chromosome of *Streptomyces albus* J1074, which does not naturally produce End, for heterologous expression (Figure S1, Supporting Information).^[^ ^10^ ^]^ HRLCMS‐based metabolite analyses revealed that the resulting *S. albus* J1074 pBAC*end* *BGC* mutant successfully acquired the capability to produce End (Figure S2, Supporting Information). This result confirmed that the *end* cluster is responsible for End biosynthesis.

Following the standard rules of type I PKS assembly lines, an initial prediction of the whole biosynthetic pathway of End was subsequently conducted. As shown in Figure S3 (Supporting Information), the process of carbon skeleton formation involved 14 condensation steps, mediated by 15 PKS modules that were distributed over 11 ORFs (*endA1‐11*). The formation of the polyether ring through epoxidation and cascade cyclization was catalyzed by EndO and EndI, which exhibited 66% and 44% similarity, respectively, to the epoxidase Lsd18 and epoxide hydrolase Lsd19, both involved in lasalocid biosynthesis.^[^ ^11^ ^]^ Additionally, EndE, with 78% similarity to type II thioesterase NanE involved in nanchangmycin biosynthesis, was envisioned to be responsible for releasing the polyether backbone.^[^ ^12^ ^]^ Notably, following the formation of the polyether backbone, a series of complex modifications, including methylation, hydroxylation, and glycosylation, are necessary to finalize the structure of End. Bioinformatics analyses revealed ten candidate tailoring genes in the *end* cluster, including three cytochrome monooxygenase P450 genes (*endP1*, *endP2*, and *endP3*), two methyltransferase genes (*endM1* and *endM2*), and five glycosyltransferase genes (*endG5*, *endG6*, *endG7*, *endG8*, and *endG9*) (Figure 2A; Table S1, Supporting Information). Correspondingly, knocking out these tailoring genes is expected to yield End analogues with altered modifying groups, such as sugar, methyl, and hydroxyl groups.

To expand the chemical diversity of End and experimentally elucidate the tailoring steps in End biosynthesis, 10 candidate tailoring genes were individually inactivated in the parent strain (Figures S5–S14, Supporting Information). Metabolite analyses of the resulting mutants revealed that the Δ*endP1*, Δ*endP3*, Δ*endM1*, and Δ*endG5* mutants lost the ability to produce End, but generated additional compounds, including three new intermediates (End‐3, End‐4, and End‐5) and one known structure (End‐2),^[^ ^13^ ^]^ whose structures were elucidated through NMR analyses (Figure 2B; Figure S26 and Tables S6 and S7, Supporting Information). Among them, End‐4, isolated from the Δ*endP1* mutant, was identified as a C30‐dehydroxylated derivative of End; *endP3* inactivation led to the production of End‐2, a C15‐deglycosylated and dehydroxylated derivative of End; End‐5, isolated from the Δ*endM1* mutant, was identified as a C4′‐demethylated derivative of End; *endG5* deletion led to the accumulation of End‐3, a C15‐deglycosylated derivative of End, and End‐2 (Figure 2B,C). All of these End analogues feature hydroxyl groups at the C11 position, facilitating the attachment of additional sugars and thereby generating new C11‐glycosylated derivatives. In addition, complementation of the Δ*endP1*, Δ*endP3*, Δ*endM1*, and Δ*endG5* mutants with functional *endP1*, *endP3*, *endM1*, and *endG5*, respectively, led to restoration of End production (Figure 2B). The inactivation of *endP2*, *endM2, endG6*, *endG7*, *endG8*, and *endG9* did not result in the loss of End, indicating that these six genes are not essential for End biosynthesis (Figure 2B). These results offered compelling evidence for elucidating the tailoring steps in End biosynthesis. As shown in Figure 2C, the tailoring steps in End biosynthesis do not comprise a linear pattern, but instead form a networked metabolic pathway. The initial compound of the tailoring process is End‐6. EndP1 catalyzed C30‐hydroxylation of the intermediates. EndP3 catalyzed C15‐hydroxylation of End‐6 and End‐2, enabling EndG5 to catalyze C15‐glycosylation to afford End‐8 and End‐5. Subsequently, EndM1 was responsible for methylating the C4′‐hydroxyl group of End‐8 and End‐5 to generate End‐4 and End.

### 2.2 Identification of a Glycosyltransferase Responsible for C11‐Glycosylation

**Figure 3:**
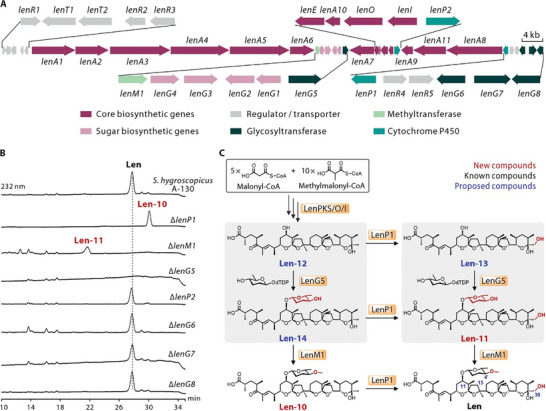
Identification of a glycosyltransferase involved in C11‐glycosylation. A) Genetic organization of the *len* cluster. B) HPLC profiles of extracts from the *S. hygroscopicus* A‐130 wild‐type strain and related mutants. C) Proposed the tailoring steps in Len biosynthesis. The C11‐glycosylation catalyzed by LenG5 is marked by gray shading. New compounds, known compounds and proposed compounds are indicated by red, black, and blue numbers, respectively.

Considering the presence of a sugar moiety at the C11 position in Len and its structural similarity to End, we hypothesize that the biosynthetic gene cluster of Len likely includes glycosyltransferase that is capable of catalyzing C11‐glycosylation of End and its analogues. However, the biosynthetic gene cluster and biosynthetic mechanism of Len have not yet been reported. Thus, to identify the glycosyltransferase responsible for catalyzing C11‐glycosylation, we performed whole‐genome sequencing of the Len‐producing strain *Streptomyces hygroscopicus* A‐130. AntiSMASH analyses revealed a type I PKS gene cluster (hereinafter designated as the *len* cluster) which was composed of 32 ORFs and exhibited significant similarity to both the *end* cluster and the biosynthetic gene cluster of nanchangmycin (**Figure** **3**A; Table S2, Supporting Information). This indicates that the *len* cluster may be responsible for Len biosynthesis. Subsequently, by employing bioinformatics analyses and taking into account the actual structure of Len, the whole biosynthetic pathway of Len was predicted, as depicted in Figure S4 (Supporting Information). Similar to the process of End biosynthesis, the production of Len also requires a series of complex post‐modification processes. Bioinformatics analyses revealed seven candidate tailoring genes, including two cytochrome monooxygenase P450 genes (*lenP1* and *lenP2*), one methyltransferase gene (*lenM1*), and four glycosyltransferase genes (*lenG5*, *lenG6*, *lenG7*, and *lenG8*) (Figure 3A; Table S2, Supporting Information).

To experimentally identify the glycosyltransferase responsible for C11‐glycosylation and elucidate the tailoring steps in Len biosynthesis, we individually knocked out seven candidate tailoring genes in the parent strain (Figures S15–S21, Supporting Information). Metabolite analyses revealed that the deletion of *lenP2*, *lenG6*, *lenG7*, and *lenG8* did not result in the abolish of Len, indicating that these four genes are not essential for Len biosynthesis. Notably, the Δ*lenP1*, Δ*lenM1*, and Δ*lenG5* mutants lost the ability to produce Len (Figure 3B). In addition, NMR analyses revealed that the Δ*lenP1* mutant produced a new compound (Len‐10), which is a C30‐dehydroxylated derivative of Len. Deletion of *lenM1* led to the production of another new compound (Len‐11), a C4′‐demethylated derivative of Len (Figure 3B,C; Figure S26 and Table S8, Supporting Information). These results underscored the pivotal roles of *lenP1*, *lenM1*, and *lenG5* in Len biosynthesis. As shown in Figure 3C, we postulated that the initial compound of the tailoring process is Len‐12. LenP1 catalyzed the C30‐hydroxylation of the intermediates. LenG5 catalyzed the C11‐glycosylation of Len‐12 and Len‐13 to afford Len‐14 and Len‐11. Subsequently, LenM1 was responsible for methylating the C4′‐hydroxyl group to generate Len‐10 and Len. Notably, the glycosyltransferase encoded by *lenG5* was responsible for catalyzing C11‐glycosylation and exhibited a certain degree of substrate promiscuity.

### 2.3 Generation of Novel Aglycone Polyether by Combinatorial Biosynthesis

**Figure 4:**
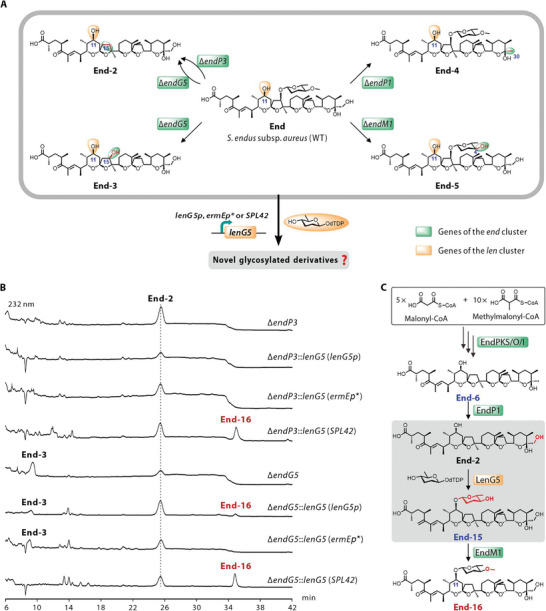
Generation of novel aglycone polyether analogue by combinatorial biosynthesis. A) Redesigned the biosynthetic pathways of End and Len to afford novel C11‐glycosylated derivatives. WT, wild‐type. B) HPLC profiles of extracts from reprogrammed mutants. C) Proposed the tailoring steps in End‐16 biosynthesis. New compounds, known compounds and proposed compounds are indicated by red, black, and blue numbers, respectively.

In the above work, we successfully identified the glycosyltransferase LenG5, which is responsible for C11‐glycosylation, demonstrating a certain degree of substrate promiscuity. In addition, a series of End analogues (End‐2, End‐3, End‐4, and End‐5) with hydroxyl groups at the C11 position were obtained through gene knockout strategy to potentially serve as substrates for LenG5 to produce C11‐glycosylated derivatives (**Figure** **4**A).

To test the above hypothesis and create novel derivatives, we conducted combinatorial expression of *lenG5* with genes that were responsible for the biosynthesis of End and its analogues in *S. endus* subsp. *aureus*. First, to precisely control the heterologous expression of *lenG5*, we used β‐glucuronidase as a readout to assess the strength of nine promoters and selected the strongest promoter *SPL42*, the moderately strong promoter ermEp^*^, and the weak promoter *lenG5p* to regulate *lenG5* expression, respectively (Figures S22 and S23, Supporting Information). Subsequently, *lenG5* was heterologously expressed in all strains that accumulated potential substrates for LenG5, including the wild‐type strain of *S. endus* subsp. *aureus*, as well as the Δ*endP1*, Δ*endP3*, Δ*endM1*, and Δ*endG5* mutants. Metabolite analyses revealed that the Δ*endP3*::*lenG5* (*SPL42*), Δ*endG5*::*lenG5* (*lenG5p*), and Δ*endG5*::*lenG5* (*SPL42*) mutants produced a novel derivative, End‐16, a C11‐glycosylated derivative of End‐2, whose structure was determined through NMR analyses (Figure 4B; Figure S26 and Table S9, Supporting Information). As illustrated in Figure 4C, the formation of End‐16 was catalyzed by enzymes from both the End and Len biosynthesis. Additionally, no new C11‐glycosylated products were detected in other reprogrammed mutants (Figure S24, Supporting Information). This indicated that LenG5 selectively recognized End‐2 as a substrate for catalyzing C11‐glycosylation, while other compounds (End‐3, End‐4, End‐5, and End) were not recognized, most likely due to steric hindrance resulting from the presence of additional hydroxyl or glycosyl groups at the C15 position. In addition, the Δ*endP3*::*lenG5* (*SPL42*), Δ*endG5*::*lenG5* (*lenG5p*), and Δ*endG5*::*lenG5* (*SPL42*) mutants produced End‐16 at titers of 4.1, 2.3, and 7.8 mg L^−1^, respectively. These titers represent a significant reduction compared to the production of End (610.4 mg L^−1^) in the wild‐type strain of *S. endus* subsp. *aureus* (Figure S25, Supporting Information). The accumulation of the intermediate End‐2, the substrate for LenG5, in the three End‐16‐producing mutants indicated that the low titer of End‐16 may be due to insufficient expression level of the LenG5 protein or the low catalytic efficiency of LenG5, likely caused by structural differences between End‐2 and LenG5's natural substrates, Len‐12 and Len‐13.

### 2.4 Antiproliferative Effects of Novel Polyether Analogues on BLCA cells

To assess the anti‐BLCA potential of all the polyether analogues obtained above (End‐2, End‐3, End‐4, End‐5, Len‐10, Len‐11, and End‐16), we examined their impact on the proliferation of various human BLCA cell lines, including four MIBC cell lines (5637, UM‐UC‐3, T24, and SCaBER) and one NMIBC cell line (RT4), with cisplatin used as a positive control. The tested compounds showed inhibitory activity against all five BLCA cell lines with IC_50_ values at the micromolar level (**Table** **1**). Moreover, the comparison between Len and Len‐11 revealed that the C4′‐methylation of D‐amicetose is critical for the activity. This conclusion was further supported by the comparison of the activities between End and End‐5. In addition, the importance of the C30‐hydroxyl group for anti‐proliferative efficacy was demonstrated by comparing Len and Len‐10, as well as End and End‐4. Notably, Len and End‐16, both of which contain complete D‐amicetose at the C11 position, demonstrated remarkable efficacy in inhibiting cell proliferation, which was consistent with our predictions. Overall, End‐16, End, and Len exhibited the most prominent inhibitory efficacy, surpassing that of cisplatin (Table 1). Considering the high toxicity of Len reported in previous studies,^[^ ^3^ ^]^ End‐16 and End were selected for further studies.

**Table 1:** IC_50_ values (µm) of polyether analogues against BLCA cells.

IC_50_ (µM)^a)^	5637	UM‐UC‐3	T24	SCaBER	RT4
End	1.24 ± 0.50	0.28 ± 0.09	0.44 ± 0.09	0.98 ± 1.19	1.07 ± 0.23
End‐5	4.30 ± 4.04	0.44 ± 0.14	2.02 ± 1.01	10.17 ± 11.79	3.72 ± 1.38
End‐2	4.02 ± 0.08	0.27 ± 0.08	0.79 ± 0.14	0.91 ± 0.69	1.27 ± 0.45
End‐3	64.74 ± 68.18	8.55 ± 6.65	18.30 ± 5.00	20.68 ± 16.03	17.58 ± 1.65
End‐4	5.06 ± 1.06	1.55 ± 0.17	1.49 ± 0.08	4.98 ± 2.98	1.89 ± 0.26
End‐16	0.89 ± 0.26	1.51 ± 0.12	0.86 ± 0.16	1.11 ± 0.56	1.27 ± 1.18
Len	0.55 ± 0.12	0.29 ± 0.03	0.43 ± 0.34	0.74 ± 0.82	0.51 ± 0.26
Len‐10	46.98 ± 41.80	12.79 ± 1.25	9.57 ± 2.33	12.78 ± 2.67	6.62 ± 2.96
Len‐11	8.49 ± 3.93	8.28 ± 6.34	3.18 ± 0.35	10.87 ± 4.91	3.51 ± 2.48
Cisplatin	2.92 ± 0.67	4.66 ± 0.74	2.48 ± 0.55	3.13 ± 0.11	3.02 ± 0.59

^a)^ IC_50_ values were compared to control and were determined after exposure of the cells to different drug concentrations for 72 h. Experiments were conducted using MTT reagent. Data presented as means ± SEMs from three independent experiments.

### 2.5 End‐16 Suppresses the Cell Cycle Progression and Motility of BLCA Cells In Vitro

**Figure 5:**
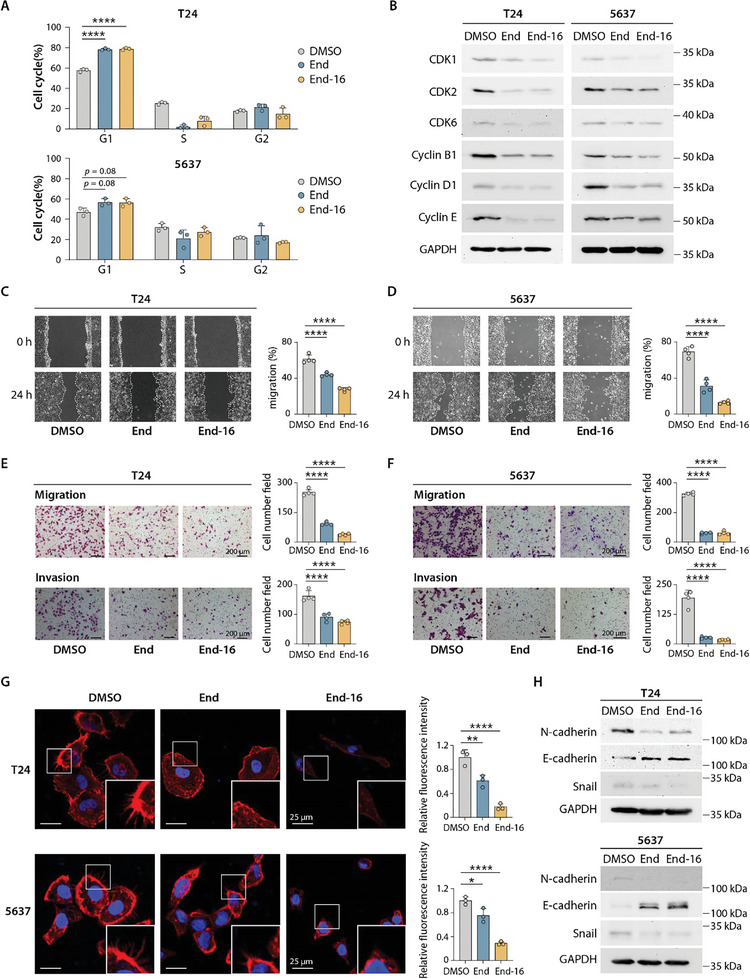
End‐16 suppresses the cell cycle progression and motility of BLCA cells. A) Cell cycle distribution based on flow cytometry analyses. Data presented as means ± SDs (n  =  3). B) Western blot analyses showing the expression of key regulators of the cell cycle control. C,D) Wound healing assay for assessing the migration ability of T24 and 5637 cells treated with End and End‐16. Data presented as means ± SDs (n  =  4). E,F) Transwell chamber migration and invasion assays for T24 and 5637 cells treated with End and End‐16. Data presented as means ± SDs (n  =  4). Scale bars, 200 µm. G) Fluorescence imaging of actin staining in T24 and 5637 cells with the indicated treatments. Scale bars, 25 µm. Data presented as means ± SDs (n  =  3). H) Expression levels of EMT markers, including N‐cadherin, E‐cadherin, and Snail in T24 and 5637 cells with the indicated treatments. Statistical analysis was carried out using one‐way analysis of variance (ANOVA) (^*^ *p* < 0.05; ^**^ *p* < 0.01; ^***^ *p* < 0.001; ^****^ *p* < 0.0001).

The progression of the cell cycle is strongly linked to the proliferative capacity of tumor cells. The impacts of End‐16 and End on the distribution of BLCA cells in the cell cycle were examined by flow cytometry, with the more aggressive MIBC cell lines T24 and 5637 selected as representative examples. As shown in **Figure** **5**A and Figure S27 (Supporting Information), the proportion of T24 and 5637 cells in the G1 phase increased noticeably after treatment with End‐16 or End compared to the control group, indicating that End‐16 and End can induce G1 phase cell cycle arrest in BLCA cells. Additionally, we analyzed the changes in the expression of cell cycle‐related proteins, such as cyclins and cyclin‐dependent kinases (CDKs), which are key regulators of cell cycle control and primary drivers of cell proliferation.^[^ ^14^ ^]^ Western blot analyses revealed that End‐16 and End decreased the expression of CDK2, CDK6, Cyclin D1, and Cyclin E, which are important regulators of G1 to S phase progression, in T24 and 5637 cells (Figure 5B). In addition, the expression of Cyclin B1 and CDK1, which regulate cell cycle from G2 to M phase, was also decreased (Figure 5B). However, we did not observe G2 phase cell cycle arrest (Figure 5A), which might be related to the drug's effect on the expression of proteins that are widely involved in multiple stages of cell cycle regulation. Similar phenomena have been reported in previous studies.^[^ ^15^ ^]^

Subsequently, to evaluate the impacts of End‐16 and End on the metastatic ability of BLCA cells, we conducted wound healing assay and transwell chamber migration and invasion assays. As shown in Figure 5C–F, the migration and invasion abilities of T24 and 5637 cells were decreased in the End‐16 and End treated groups compared to the control group, with End‐16 exhibiting superior inhibitory effects compared to End. The migration and invasion abilities of tumor cells are closely related to changes in cell morphology and the formation of local membrane protrusions.^[^ ^16^ ^]^ Actin staining revealed a reduction in membrane protrusions, changes in cell morphology, and decreased expression of actin in T24 and 5637 cells after treatment with End‐16 and End. These changes were more pronounced in End‐16‐treated cells (Figure 5G), further supporting the results of the wound healing assay and transwell chamber migration and invasion assays (Figure 5C–F). Epithelial‐mesenchymal transition (EMT) is the initial stage of tumor cells metastasis and plays a crucial role in promoting tumor invasion and metastasis.^[^ ^17^ ^]^ So we analyzed the changes in the expression of EMT‐associated protein markers, including the epithelial cell marker E‐cadherin, the mesenchymal cell marker N‐cadherin, and the EMT transcription factor Snail, which suppresses the expression of E‐cadherin, in BLCA cells treated with End‐16 and End, respectively.^[^ ^18^ ^]^ Western blot analyses revealed that the expression of E‐cadherin was noticeably upregulated, while the expression of N‐cadherin and Snail was noticeably downregulated in BLCA cells (T24 and 5637) treated with End‐16 and End, respectively, indicating that End‐16 and End have the ability to inhibit the EMT programs (Figure 5H). The above results suggest that both End‐16 and End can inhibit the metastatic ability of BLCA cells by inhibiting the formation of cellular protrusions and EMT programs, with End‐16 exhibiting more pronounced inhibitory effects.

### 2.6 End‐16 Exhibits High Potency and Low Side Effects Against BLCA In Vivo

**Figure 6:**
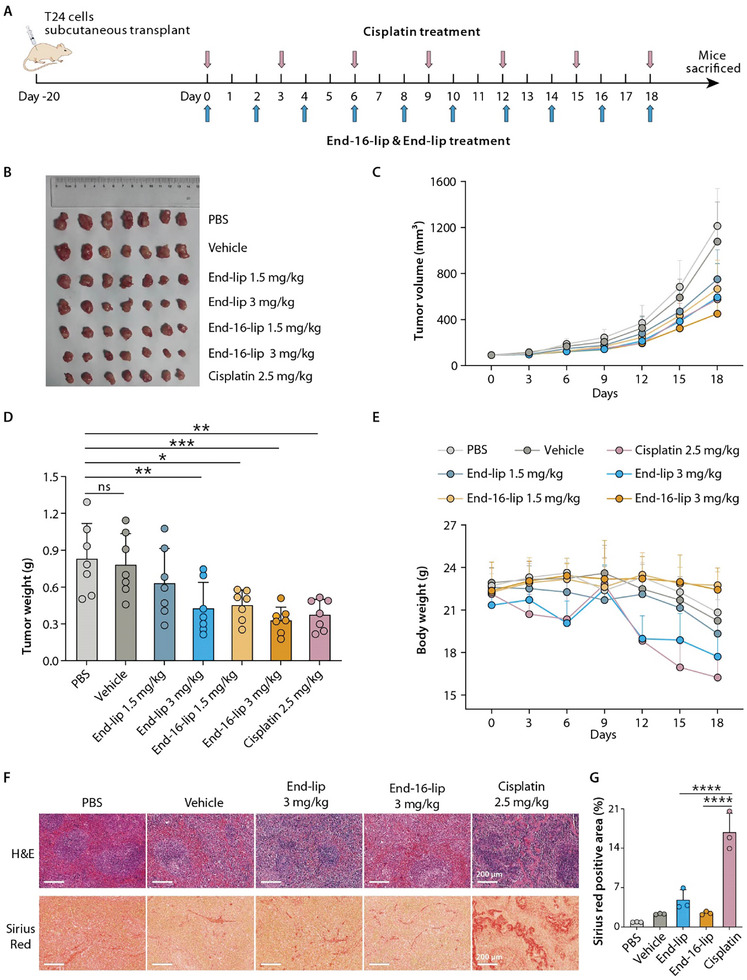
End‐16‐lip and End‐lip inhibit BLCA in vivo. A) Schematic showing the treatment for the subcutaneous xenograft model with T24 cells. B) Image of tumor tissues from mice in each group (n  =  7). C) Time‐course tumor volume curves of the mice (n  =  7). D) Tumor weight in each group (n  =  7). E) Time‐course body weight curves of the mice (n  =  7). F) Representative images of hematoxylin and eosin (H&E) and Sirius Red‐stained spleen sections. Scale bars, 200 µm. G) Statistical analyses of Sirius Red‐stained spleen sections (n  =  3). End‐lip, 3 mg kg^−1^; End‐16‐lip, 3 mg kg^−1^; Cisplatin, 2.5 mg kg^−1^. Data presented as means ± SDs. Statistical analysis was carried out using one‐way analysis of variance (ANOVA) (^*^ *p* < 0.05; ^**^ *p* < 0.01; ^***^ *p* < 0.001; ^****^ *p* < 0.0001; and ns, not significant).

We next examined the in vivo antitumor effects of End‐16 and End using a subcutaneous xenograft model. Considering the poor water solubility of these compounds, liposomes were opted as the delivery vehicle for End‐16 and End due to their ability to encapsulate hydrophobic drugs within their lipid bilayer.^[^ ^19^ ^]^ For administration, End‐16 and End were first prepared as liposomal formulations (End‐16‐lip and End‐lip). After establishing subcutaneous xenograft models using T24 cells, which are more sensitive to cisplatin compared to other BLCA cell lines (Table 1), we assessed the effects of End‐16‐lip and End‐lip on BLCA growth in vivo, with cisplatin used as a positive control, and PBS and drug free liposomes (vehicle) used as negative controls (**Figure** **6**A). As shown in Figure 6B–D, compared to the PBS and vehicle groups, both End‐16‐lip and End‐lip effectively suppressed tumor growth in a concentration‐dependent manner. In particular, the End‐16‐lip (3 mg kg^−1^) group exhibited the most significant inhibition in BLCA growth, surpassing both the End‐lip and cisplatin groups.

Regarding the safety profile of the drugs, from the perspective of mouse weight changes, it was obsserved that mice in the End‐16‐lip groups maintained a consistent weight throughout the entire treatment period, while the End‐lip and cisplatin groups exhibited obvious weight loss. The factors contributing to weight changes during treatment may include drug toxicity, tumor burden, and the effects of the drug on the mice's diet (Figure 6E). Compared to mice in the End‐16‐lip, PBS and vehicle groups, the spleen size of mice in the End‐lip and cisplatin groups was obviously smaller (Figure S28, Supporting Information). Furthermore, H&E and Sirius Red staining revealed numerous histopathological changes and the presence of a large number of collagen deposits in the spleens of mice in the cisplatin group (Figure 6F,G), as well as histopathological changes in the livers, including hepatocyte necrosis and inflammatory cells infiltration (Figure S29, Supporting Information). Notably, compared to the PBS groups, mice in the End‐16‐lip and End‐lip groups also displayed histopathological changes in spleen and liver tissues, but the extent of damage was obviously less severe than the cisplatin group (Figure 6F,G; Figure S29, Supporting Information). Some abnormalities in the splenic architecture were also observed in the vehicle group (Figure 6F,G), which may be due to the accumulation of liposomes in the spleen, triggering an immune response and causing some tissue damage. Previous study has reported that liposomes primarily accumulate in organs (e.g., the spleen) that contain the mononuclear phagocyte system, which is responsible for removing antigens from the body, and may trigger an immune response.^[^ ^20^ ^]^

In our animal experiments, we found that 1) End‐16 exhibits superior inhibitory activity against BLCA compared to End and cisplatin in vivo; 2) unlike End and cisplatin, End‐16 did not cause weight loss of the mice; and 3) the liver and spleen damage in End‐16 groups was relatively less severe than that in the cisplatin group. Based on the above results, we concluded that End‐16 exhibits higher potency and fewer side effects than End and cisplatin, the current first‐line chemotherapy drug for BLCA treatment. The results indicate that exploring C11‐glycosylated derivatives based on End and its analogues can effectively enhance the anticancer efficacy and decrease the toxicity of aglycone polyethers.

## 3 Conclusion

In summary, guided by the structure‐activity relationships of aglycone polyethers and based on biosynthetic mechanisms of End and Len, we successfully create a novel aglycone polyether End‐16, with potential clinical applications in the treatment of BLCA, using combinatorial biosynthesis strategies. End‐16 demonstrated potent inhibitory effects on the proliferation and motility of BLCA cells in vitro. Importantly, End‐16 exhibited superior inhibitory activity and fewer side effects against BLCA compared to the frontline clinical drug cisplatin in vivo. These findings highlight the importance of conducting additional preclinical studies on End‐16, encompassing pharmacology, toxicology, and pharmacokinetic investigations, to further explore its therapeutic potential in the treatment of BLCA. Moreover, the anti‐cancer efficacy of End‐16 may extend beyond BLCA, warranting studies involving other types of cancer that have high rates of occurrence and mortality, such as breast cancer, liver cancer, colorectal cancer, and prostate cancer.^[^ ^5^ ^]^ In addition, the current study offers crucial insight into the biosynthesis of polyethers and serves as a compelling example of combinatorial biosynthesis for generating unnatural products with improved pharmacological properties.

## 4 Experimental Section

### General Experimental Procedures

General reagents, media, and enzymes were sourced from commercial suppliers. The synthesis of oligonucleotide primers was completed by Wuhan GeneCreate Biological Engineering Co., Ltd. DNA sequencing was performed by Tsingke Biotechnology Co., Ltd. Details of specific bacterial strains and plasmids employed in this research were listed in Tables S3 and S4 (Supporting Information), and the PCR primers were listed in Table S5 (Supporting Information). HPLC analysis was conducted using a Thermo Ultimate 3000 HPLC system equipped with C18 columns (4.6 mm × 150 mm, 5 µm, Agilent Technologies, Inc.). The preparative HPLC was performed using SEP LC‐52 with an MWD UV detector with Waters Xterra RP C18 (3.9 mm × 150 mm, 5 µm) column. HR‐ESI‐LCMS analysis was performed using Thermo Fisher Scientific Ultra High Resolution Linear Ion Trap Orbitrap Mass Spectrometer (LTQ Orbitrap Elite) in positive ESI mode, equipped with Thermo Scientific Hypersil GOLD C18 AQ (2.1 mm × 150 nm, 3 µm). The UV spectra were recorded using a Shimadzu UV‐2401 PC spectrophotometer. IR data were obtained using a JASCO FT/IR‐480 plus spectrometer. Optical rotations were determined via a JASCO P‐1020 polarimeter. ECD spectra were acquired using a JASCO J‐810 spectrophotometer. 1D and 2D NMR data were recorded on the Bruker Avance NEO 600 MHz NMR spectrometer, with CDCl_3_ used as the solvent for NMR measurements.

### Strains and Culture Conditions

*S. endus* subsp. *aureus* and *S. hygroscopicus* A‐130 were purchased from the American Type Culture Collection. The strains were cultivated on soybean flour‐mannitol (SFM) agar plates (2% g L^−1^ soybean flour, 2% g L^−1^ mannitol, and 1.6% g L^−1^ agar, pH 7.2) at 30 °C for spore collection. The strains were grown in shaking flasks in TSB medium (3% g L^−1^ tryptone soya broth) at 30 °C, 220 rpm, for 2 days to serve as the source for whole‐genome sequencing. *S. albus* J1074 was used as the host for heterologous expression of the *end* gene cluster. Conjugation experiments were conducted on SFM plates. For the analysis and isolation of the derivatives, the *Streptomyces* strains were cultivated at 30 °C, 220 rpm for 2 days in TSB medium (the seed culture). The cells were then inoculated into 250 mL conical flask containing 50 mL NO.3 fermentation broth (1% g L^−1^ soybean flour, 3% g L^−1^ soluble starch, 0.25 g L^−1^ yeast extract, 0.3% g L^−1^ CaCO_3_, pH 7.2) or 1 L conical flasks containing 900 mL NO.3 fermentation broth, and the culture was continued at 30 °C on a rotary shaker at 220 rpm for 8 days.

*E. coli* DH10B was used for cloning. *E. coli* ET12567/pUZ8002 and *E. coli* ET12567/pUB307 were used for conjugation. These strains were cultured in LB broth (10 g L^−1^ tryptone, 5 g L^−1^ yeast extract, 10 g L^−1^ NaCl) supplemented with appropriate antibiotics (100 mg L^−1^ ampicillin or 50 mg L^−1^ apramycin). *S. cerevisiae* CEN.PK2‐1D was also utilized for cloning and was cultured in YPD medium (2% g L^−1^ tryptone, 1% g L^−1^ yeast extract, and 2% g L^−1^ glucose).

### Direct Cloning and Heterologous Expression of the end Gene Cluster

Genomic DNA of *S. endus* subsp. *aureus*, recovered from lysate using phenol‐chloroform‐isoamyl alcohol (25:24:1, pH 8.0) extraction and ethanol precipitation method, was digested with NheI for subsequent ExoCET cloning.^[^ ^21^ ^]^ The p15A vector used to clone the 52 kb fragment released via NheI digestion and the pBeloBAC11 vector used to clone the 72 kb fragment released via NheI digestion were amplified via PCR. After these two rounds of PCR, the homology arms for ExoCET cloning and the 50 bp overlap with the NheI terminus of the 52 and the 72 kb fragment were attached to the p15A vector and pBeloBAC11 vector, respectively. The mixture of 10 µg digested genomic DNA and linear cloning vector (200 ng p15A linear vector or 600 ng pBeloBAC11 linear vector) was treated with 0.02 U/µL T4 pol. The in vitro assembly products were desalted for 30 min and electroporated into *E. coli* GB05‐dir harboring pSC101‐BAD‐ETgA‐tet. The recombinant pBeloBAC11‐72‐kb plasmid containing the 72 kb fragment and the recombinant p15A‐52‐kb plasmid containing the 52 kb fragment were identified via restriction enzyme analysis. Then, the apra‐oriT‐attP‐int cassette was inserted into p15A‐52‐kb by Redαβ recombineering for intergeneric conjugation between *E. coli* and *Streptomyces*.^[^ ^22^ ^]^ The 52 kb fragment and the apra‐oriT‐attP‐int cassette in p15A vector were cloned to pBeloBAC11‐72‐kb to produce pBAC*endBGC* via the Gibson assembly method. The correct recombinant plasmid pBAC*endBGC* was identified via AgeI, EcoNI, PvuII, and PstI restriction analysis.

The resulting plasmid pBAC*endBGC* was transferred into *S. albus* J1074 through conjugation. The exconjugants were selected based on cell phenotype showing resistance to apramycin (50 mg L^−1^) and then confirmed by PCR, generating the mutant *S. albus* J1074 pBAC*endBGC*, wherein the plasmid pBAC*endBGC* was integrated into the chromosome of *S. albus* J1074.

### Construction of Gene Mutant Strains

In‐frame deletion of genes in *S. endus* subsp. *aureus* and *S. hygroscopicus* A‐130 relied on homologous recombination. Ten genes (*endP1*, *endP2*, *endP3*, *endM1*, *endM2*, *endG5*, *endG6*, *endG7*, *endG8*, and *endG9*) in *S. endus* subsp. *aureus* and seven genes (*lenP1*, *lenP2*, *lenM1*, *lendG5*, *lenG6*, *lenG7*, and *lenG8*) in *S. hygroscopicus* A‐130 were deleted, respectively. As an illustration, *endM1* was employed to introduce the inactivation of genes. For *endM1* inactivation, a 2.0 kb XbaI / HindIII fragment and a 2.1 kb NdeI / XbaI fragment were cloned into the NdeI / HindIII sites of the plasmid pYH7 to generate the *endM1* deletion plasmid, pYH7‐*endM1*. pYH7‐*endM1* was transformed into *E. coli* ET12567/pUZ8002 and then transferred into *S. endus* subsp. *aureus* by conjugation. The exconjugants were initially cultured on SFM plates with apramycin (50 mg L^−1^) and trimethoprim (50 mg L^−1^), at 30 °C to obtain single‐crossover mutants. Subsequently, these mutants were further cultured at 30 °C on SFM plates devoid of apramycin to yield apramycin‐sensitive clones. The double‐crossover mutant *S. endus* subsp. *aureus* Δ*endM1* was confirmed by PCR analysis.

### Construction of Gene Complementation Strains

*endM1* was used as an example to introduce the complementation of specific genes. For gene *endM1* complementation, a 1.5 kb NdeI / XbaI fragment was cloned into the NdeI / XbaI sites of pIB139 with the ermEp* promoter to generate the plasmid pIB139‐*endM1*. pIB139‐*endM1* was transformed into *E. coli* ET12567/pUZ8002 and transferred into *S. endus* subsp. *aureus* Δ*endM1* mutant strain through conjugation. The exconjugants were selected based on cell phenotype exhibiting resistance to apramycin (50 mg L^−1^) and subsequently verified via PCR analysis, generating the complementation strain *S. endus* subsp. *aureus* Δ*endM1*::*endM1* mutant.

### Test of Promoter Strength Based on GusA Activity

The promoter strength was evaluated based on the enzymatic activity exhibited by the reporter protein GUS. A total of 9 promoters were selected for evaluation. Three promoters, *endP3p*, *endM1p* (from *S. endus* subsp. *aureus*), and *lenG5p* (from *S. hygroscopicus* A‐130), were cloned into pSET152 upstream of the *gusA* reporter gene. The resulting constructs, pGUS‐*lenG5p*, pGUS‐*endP3p*, pGUS*‐endM1p*, and six plasmids from previous reports,^[^ ^23^ ^]^ including pLH2 (containing promoter *SPL42*), pLH5 (containing promoter *SRL37*), pLH8 (containing promoter *rpsLp‐cf*), pLH9 (containing promoter *kasOp‐rpsL‐CF*), pLH10 (containing promoter *kasOp* ^*^), and pLH18 (containing promoter ermEp^*^), were transformed into *E. coli* ET12567/pUZ8002 and transferred into *S. endus* subsp. *aureus* wild‐type strain through conjugation. The strength of the promoter harbored by each mutant was evaluated according to a previous report.^[^ ^24^ ^]^

### Construction of Hybrid Strains

The *lenG5* was heterologously expressed, under the control of the natural promoter (*lenG5p*), ermEp* and *SPL42* in wild‐type *S. endus* subsp. *aureus*, Δ*endP1*, Δ*endP3*, Δ*endM1*, and Δ*endG5* mutants. For the expression of *lenG5*, a 1.4 kb NdeI / KpnI fragment was cloned into the NdeI / KpnI sites of pSET152 with the natural promoter (*lenG5p*), ermEp* or *SPL42*, to generate pYL1, pYL2, and pYL3, respectively. The resulting plasmids were, respectively, transformed into *E. coli* ET12567/pUZ8002 and transferred into wild‐type *S. endus* subsp. *aureus*, Δ*endP1*, Δ*endP3*, Δ*endM1*, and Δ*endG5* mutants through conjugation. After sporulation on SFM medium with apramycin (50 mg L^−1^), the hybrid mutants were selected based on apramycin‐resistant and verified by PCR analysis.

### Metabolite Analyses of Wild‐Type Strains and Related Derivative Strains

After small‐scale fermentation of the strains, the fermentation broth was centrifuged at 5000 rpm for 6 min to collect the mycelia, which were then subjected to methanol extraction twice. To ensure chemical stability, a certain amount of ammonium acetate was added during the extraction process. The dried methanol‐extracted layer was dissolved in 1.5 mL methanol, and 10 µL of each sample was subsequently injected into HPLC for analysis. The extracts of *S. endus* subsp. *aureus* and related derivative strains were analyzed by HPLC, eluted with acetonitrile‐H_2_O (0.1% FA) system (70:30 for 5 min, a linear gradient from 70:30 to 100:0 within the following 30 min, and 100:0 for 15 additional min) at a flow rate of 1 mL min^−1^ and with UV detection at 232 nm. The extracts of *S. hygroscopicus* A‐130 and related derivative strains were detected by HPLC, eluted with acetonitrile‐H_2_O (0.1% FA) system (80:20 for 5 min, a linear gradient from 80:20 to 100:0 within the following 30 min, and 100:0 for 15 additional min).

HR‐ESI‐LC‐MS analysis was carried out using Thermo Fisher Scientific Ultra High Resolution Linear Ion Trap Orbitrap Mass Spectrometer (LTQ Orbitrap Elite) in positive ESI mode. The mobile phase of LC‐MS comprises solvents A (ddH_2_O supplemented with 0.1% FA) and B (acetonitrile). The elution was carried out using a linear gradient from 20% to 100% solvent B over 20 min, followed by 100% solvent B for 6 min, all at a flow rate of 0.15 mL mi^−1^n.

### Isolation and Structure Elucidation of Intermediates and Derivatives

The mutants were fermented on a larger scale and the resulting fermentation broth was centrifuged at 5500 rpm for 15 min. The mycelia were harvested and subjected to thrice methanol extractions. The methanol‐extracted layer was distilled under reduced pressure, followed by EtOAc/H_2_O extraction to acquire the EtOAc fraction. The crude extracts were subjected to silica gel (100–200 mesh) column chromatography using a gradient of petroleum ether/ethyl acetate gradient (10:0, 9:1, 8:2, 7:3, 5:5, 0:10, v/v, 1.5‐2 L volume for each fraction). The resulting fractions were analyzed using HPLC for identification. The fractions containing the target compounds were then combined and subjected to reversed‐phase liquid chromatography filled with LiCroprepR RP‐18 (40–63 mm) eluted in a methanol/H_2_O gradient (70:30, 75:25, 80:20, 85:15, 90:10, 100:0, v/v, 1–1.5 L volume for each fraction). Further purification was conducted using preparative HPLC. The elution was constant at 80% acetonitrile to afford the target compounds. The fragments were detected using UV light at 232 nm. The product structure was identified by HR‐ESI‐MS and NMR. The NMR data were summarized in Tables S6–S9 (Supporting Information) and Figure S26 (Supporting Information).

End‐2: white solid, [α]_D_^26^ +49.08 (*c* 0.1, Methanol); NMR spectra data see Table S6; HRESIMS [M+NH_4_]^+^ m/z 740.4957 (calculated for C_40_H_70_O_11_N, 740.4943; Δppm = 1.9).

End‐3: white solid, [α]_D_^25^ +44.72 (*c* 0.1, Methanol); NMR spectra data see Table S6 (Supporting Information); HRESIMS [M+NH_4_]^+^ m/z 756.4906 (calculated for C_40_H_70_O_12_N, 756.4892; Δppm = 1.9).

End‐4: white solid, [α]_D_^25^ +39.84 (*c* 0.1, Methanol); NMR spectra data see Table S7 (Supporting Information); HRESIMS [M+NH_4_]^+^ m/z 868.5808 (calculated for C_47_H_82_O_13_N, 868.5780; Δppm = 3.2).

End‐5: white solid, [α]_D_^26^ +33.72 (*c* 0.1, Methanol); NMR spectra data see Table S7 (Supporting Information); HRESIMS [M+NH_4_]^+^ m/z 870.5594 (calculated for C_46_H_80_O_14_N, 870.5573; Δppm = 2.4).

Len‐10: white solid, [α]_D_^25^ +32.80 (*c* 0.1, Methanol); NMR spectra data see Table S8 (Supporting Information); HRESIMS [M+NH_4_]^+^ m/z 852.5848 (calculated for C_47_H_82_O_12_N, 852.5831; Δppm = 2.0).

Len‐11: white solid, [α]_D_^25^ +34.84 (*c* 0.1, Methanol); NMR spectra data see Table S8 (Supporting Information); HRESIMS [M+NH_4_]^+^ m/z 854.5644 (calculated for C_46_H_80_O_13_N, 854.5624; Δppm = 2.3).

End‐16: white solid, [α]_D_^26^ +63.08 (*c* 0.1, Methanol); NMR spectra data see Table S9 (Supporting Information); HRESIMS [M+NH_4_]^+^ m/z 868.5797 (calculated for C_47_H_82_O_13_N, 868.5780; Δppm = 2.0).

### Cells Culture

Vero (African green monkey epithelial kidney) cells were cultured in Dulbecco's modified Eagle's medium (DMEM; Invitrogen) with 10% fetal bovine serum (FBS), 100 U mL^−1^ of penicillin and 100 µg mL^−1^ of streptomycin at 37°C with 5% CO₂. The human BLCA cell lines 5637 (Cat. #TCHu 1), UM‐UC‐3 (Cat. # TCHu217), RT4 (Cat. # TCHu226), SCaBER (Cat. # TCHu239) and T24 (Cat. # TCHu 55) were obtained from the Chinese Academy of Sciences Cell Bank (Shanghai, China) and were identified by Cell Bank, Chinese Academy of Sciences (Shanghai, China). 5637, SCaBER and T24 cell lines were cultured with RPMI‐1640 medium, containing 10% FBS. UM‐UC‐3 was cultured with DMEM medium (containing 10% FBS). RT4 was cultured with McCOY's 5A medium (containing 10% FBS). All the cells were cultured and maintained in a 37 °C incubator with 5% CO_2_.

### Cytotoxicity Assay

The tumor cells were seeded in 96‐well plates at a density of 3000 cells per well, cultured for 24 h and treated with End, End‐2, End‐3, End‐4, End‐5, End‐16, Len, Len‐10, Len‐11, or an equal amount of DMSO, or cisplatin (0, 0.00128, 0.0064, 0.032, 0.16, 0.8, 4, 20, and 100 µm, diluted in DMSO) for 72 h. After adding 20 µL 5 mg mL^−1^ MTT (Sigma, USA) to each well and incubating for 4 h at 37 °C, removing the medium and dissolving formazan precipitate in 150 µL DMSO. Finally, the absorbance of each well at 570 nm was measured using a microplate reader (Molecular Devices, USA).

### Flow Cytometry Analysis for Cell Cycle

Cell cycle was assessed by cell cycle staining kit (Multi sciences, China) according to the manufacturer's instructions. Drugs treated BLCA cells and control cells were harvested and washed twice with cold PBS. After centrifuged at 200 g for 10 min, the supernatant was removed, and the cells were resuspended and incubated with 1x DNA staining solution and permeabilization solution for 30 min at room temperature in the dark. Finally, flow cytometry (Beckman Cytoflex) was used to detect the samples and FlowJo software (version 10) was used to analyze the results.

### Wound Healing Assay

BLCA cells were seeded in 6‐well plates, scratched with a 200 µL pipette tip, and washed with PBS, after which 0.5% FBS medium (containing 2 µm End or End‐16) was added. The equal amount of DMSO was added to the control group. The results were photographed by phase contrast microscope at 0 and 24 h in several pre‐labeled points. Then, the gap closure was statistically analyzed.

### Transwell Chamber Migration and Invasion Assays

For migration assay, BLCA cells were treated with End, End‐16, or an equal amount of DMSO for 48 h. A total of 8 × 10^4^ 5637 cells or 3 × 10^4^ T24 cells were suspended in 200 µL serum‐free medium in the upper transwell chambers (Corning, USA), and 600 µL medium containing 10% FBS in the lower chambers to induce cell migration. After incubation for 24 h, the cells were fixed with 4% paraformaldehyde (PFA) and stained by 0.1% crystal violet. The number of migrated cells was counted by phase contrast microscope and statistically analysed. For invasion assay, transwell chambers were precoated with Matrigel (Sigma–Aldrich, USA). All other steps were the same as in the transwell migration assay.

### The Aglycone Polyethers Liposome Preparation

The liposome was charged with a bilayer composed of Soybean Lecithin, cholesterol, DSPE‐PEG‐COOH‐3400 and compound at 4:1:0.5:1 weight ratio, then mixed in organic solvent, evaporated using a dry nitrogen or rotary evaporation, and dissolved with PBS. The crude liposome disrupts using sonic energy (sonication), and then liposome extrusion was forced through a polycarbonate filter with a defined pore size to yield particles, and average size of particles was measured by Malvern DSL with ≈100 nm.

### Actin Staining

Drugs treated BLCA cells were plated on 12 mm coverslips and incubated for 12 h. After the cells adhered, the coverslips were fixed with 4% PFA for 30 min and washed 2–4 times with PBS containing 0.1% Triton X‐100. Next, the coverslips were incubated with the Actin‐Tracker Red‐594 (Beyotime, China) for 30–60 min. Finally, cells were incubated with DAPI for 5 min to label the nuclei. The actin staining was observed and photographed by confocal microscopy (Nikon, Japan). The fluorescence intensity of actin was evaluated by ImageJ software.

### Western Blot Analysis

BLCA cells were collected and lysed in RIPA buffer (containing protease inhibitor and phosphatase inhibitor) on ice for 30 min. The cell lysates were centrifuged at 12000 g for 15 min, and the supernatant was collected. For western blot analysis, total protein was separated using 12.5% SDS‐PAGE gels, then transferred to PVDF membrane (Millipore, USA). Membranes were blocked in 5% TBST fat‐free milk for 2 h at room temperature. The membranes were cut horizontally according to the protein marker (Shanghai Epizyme, China) on the membrane and incubated separately with the appropriate primary antibody (Table S10, Supporting Information) for overnight at 4 °C. After washing three times with TBST, membranes were incubated with secondary antibody (Table S10, Supporting Information) for 2 h at room temperature. Bands were detected using an enhanced chemiluminescence kit (Bio‐Rad, USA) and blots were exposured to the BioSpectrum Gel Doc‐IT2 315 167 Imaging System (UVP, USA).

### Animal Experiments

This study was approved by the Experimental Animal Welfare and Ethics Committee of Zhongnan Hospital (approval number: ZN2023159). 4‐week‐old male BALB/c‐nude mice were purchased from Beijing Vital River Laboratory Animal Technology Co. Ltd (Beijing, China). All mice were housed under specific‐pathogen‐free conditions in a controlled environment room (temperature 20–24 °C, relative humidity 30–70% and 12 h light‐dark cycle) and allowed unrestricted access to food and water in the animal experiment center of Zhongnan Hospital of Wuhan University.

### Subcutaneous Tumor Model

The subcutaneous tumor model was established by subcutaneous injection of 150 µL PBS solution, containing 4 × 10^7^ T24 cells, in the dorsal region near the forelimb of 4‐week‐old BALB/c‐nude mice. Twenty days later, mice were randomly divided into seven groups (n = 7). End and End‐16 were administered intraperitoneally at a dose of 1.5 or 3 mg kg^−1^ every other day, cisplatin (MCE, USA) was administered intraperitoneally at a dose of 2.5 mg kg^−1^ every 3 days, and the control group received an equal amount of PBS or liposomes via intraperitoneal injection. The tumor size was measured with a vernier caliper and calculated according to the formula (tumor size = length × width^2^ × 0.5 mm^3^) at regular intervals. When the drug injection experiments were completed, mice were sacrificed by cervical dislocation, and the xenograft tumors, livers, and spleens were collected and fixed in 4% PFA in preparation for subsequent staining.

### Hematoxylin and Eosin (H&E) Staining

The collected fresh tissue samples were fixed in 4% PFA, embedded in paraffin, and cut into 5 µm sections. Then the samples were sequentially processed with xylene, graded alcohol (100%, 96%, 80%, 70% ethanol) and H_2_O. Then 10% hematoxylin (Sigma–Aldrich, USA) was used for variegation for 5–10 min and washed with water. 1% eosin (Sigma‐‐Aldrich, USA) and 0.2% glacial acetic acid were applied to highlight the cytoplasm for only seconds and washed with water again. Then samples were dehydrated in graded alcohol and xylene in turn. Finally, an inverted phase contrast microscope (Leica, Germany) was used for observation and photography.

### Sirius Red Staining

The collected fresh tissue samples were fixed in 4% PFA, embedded in paraffin and sectioned. Subsequently, sections were dewaxed, hydrated, stained with sirius red (0.1% sirius red in saturated picric acid), dehydrated, mounted, and observed and photographed using a microscope. The sirius red positive area (%) was evaluated by ImageJ software.

### Statistical Analysis

Data were presented as the mean ± SEM or mean ± SD. Statistical analysis was carried out using one‐way analysis of variance (ANOVA). Prism 9.0.0 (GraphPad Software) was used for data analysis and graph plotting. In the figures, asterisks represent the following *p* values: ^*^ *p* < 0.05, ^**^ *p* < 0.01, ^***^ *p* < 0.001, and ^****^ *p* < 0.0001.

## Conflict of Interest

The authors declare the following competing financial interest(s): Wuhan Hesheng Technology Co., Ltd has applied for several patents based on this work.

## Supporting information

Supporting Information

## Data Availability

The data that support the findings of this study are available from the corresponding author upon reasonable request.
