# Supplementary material for: Combinatorial Biosynthesis Creates a Novel Aglycone Polyether with High Potency and Low Side Effects Against Bladder Cancer
Source: Adv Sci (Weinh). 2024 Jun 27;11(32):2404668. doi: 10.1002/advs.202404668 (PMC11348059; doi:10.1002/advs.202404668)
Supplement: Supplementary file 1 — Supporting Information [file ADVS-11-2404668-s001.pdf]

ADVANCED SCIENCE
Open Access

Supporting Information

for *Adv. Sci.*, DOI 10.1002/advs.202404668

Combinatorial Biosynthesis Creates a Novel Aglycone Polyether with High Potency and Low Side Effects Against Bladder Cancer

Pan Yan, Gang Wang, Minjian Huang, Zhen Liu, Chong Dai, Ben Hu, Meijia Gu, Zixin Deng, Ran Liu, Xinghuan Wang* and Tiangang Liu**

Supplementary Information

Combinatorial biosynthesis creates a novel aglycone polyether with high potency and low side effects against bladder cancer

Pan Yan,[+] Gang Wang,[+] Minjian Huang,[+] Zhen Liu, Chong Dai, Ben Hu, Meijia Gu, Zixin Deng, Ran Liu, Xinghuan Wang,* and Tiangang Liu**

[+] These authors contributed equally to this work.

* Corresponding authors: l_ran@sjtu.edu.cn; wangxinghuan@whu.edu.cn; liutg@whu.edu.cn

Table of contents

Supplementary Tables 4

Supplementary Table 1. Deduced functions of ORFs in the *end* gene cluster. 4

Supplementary Table 2. Deduced functions of ORFs in the *len* gene cluster. 6

Supplementary Table 3. Strains used in this study. 8

Supplementary Table 4. Plasmids used in this study. 10

Supplementary Table 5. Primers used in this study. 12

Supplementary Table 6. The ^{1}H (600 MHz) and ^{13}C NMR (150 MHz) data for End-2 and End-3 in $CDCl_3$ (δ in ppm, J in Hz). 17

Supplementary Table 7. The ^{1}H (600 MHz) and ^{13}C NMR (150 MHz) data for End-4 and End-5 in $CDCl_3$ (δ in ppm, J in Hz). 18

Supplementary Table 8. The ^{1}H (600 MHz) and ^{13}C NMR (150 MHz) data for Len-10 and Len-11 in $CDCl_3$ (δ in ppm, J in Hz). 20

Supplementary Table 9. The ^{1}H (600 MHz) and ^{13}C NMR (150 MHz) data for End-16 in $CDCl_3$ (δ in ppm, J in Hz). 21

Supplementary Table 10. Primary and secondary antibodies. 23

Supplementary Figures 24

Supplementary Figure 1. Cloning of full set of *end* BGC. 24

Supplementary Figure 2. Heterologous expression of the *end* BGC. 25

Supplementary Figure 3. Proposed biosynthetic pathway to endusamycin in *S. endus* subsp. *aureus*. 26

Supplementary Figure 4. Proposed biosynthetic pathway to lenoremycin in *S. hygroscopicus* A-130. 27

Supplementary Figure 5. Disruption of *endP1* in *S. endus* subsp. *aureus* via homologous recombination. 28

Supplementary Figure 6. Disruption of *endP2* in *S. endus* subsp. *aureus* via homologous recombination. 28

Supplementary Figure 7. Disruption of *endP3* in *S. endus* subsp. *aureus* via homologous recombination. 29

Supplementary Figure 8. Disruption of *endM1* in *S. endus* subsp. *aureus* via homologous recombination. 29

Supplementary Figure 9. Disruption of *endM2* in *S. endus* subsp. *aureus* via homologous recombination. 30

Supplementary Figure 10. Disruption of *endG5* in *S. endus* subsp. *aureus* via homologous recombination. 30

Supplementary Figure 11. Disruption of *endG6* in *S. endus* subsp. *aureus* via homologous recombination. 31

Supplementary Figure 12. Disruption of *endG7* in *S. endus* subsp. *aureus* via homologous recombination. 31

Supplementary Figure 13. Disruption of *endG8* in *S. endus* subsp. *aureus* via homologous recombination. 32

Supplementary Figure 14. Disruption of *endG9* in *S. endus* subsp. *aureus* via homologous recombination. 32

Supplementary Figure 15. Disruption of *lenP1* in *S. hygroscopicus* A-130 via homologous recombination. 33

Supplementary Figure 16. Disruption of *lenP2* in *S. hygroscopicus* A-130 via homologous recombination. 33

Supplementary Figure 17. Disruption of *lenM1* in *S. hygroscopicus* A-130 via homologous recombination. 34

Supplementary Figure 18. Disruption of *lenG5* in *S. hygroscopicus* A-130 via homologous recombination. 34

Supplementary Figure 19. Disruption of *lenG6* in *S. hygroscopicus* A-130 via homologous recombination. 35

Supplementary Figure 20. Disruption of *lenG7* in *S. hygroscopicus* A-130 via homologous recombination. 35

Supplementary Figure 21. Disruption of *lenG8* in *S. hygroscopicus* A-130 via homologous recombination. 36

Supplementary Figure 22. Strength evaluation of selected promoters in *S. endus* subsp. *aureus*. . 37

Supplementary Figure 23. HPLC profiles of extracts from the *S. hygroscopicus* A-130 wild-type, Δ*lenG5* mutant and gene complementation strains. 37

Supplementary Figure 24. HPLC profiles of extracts from *S. endus* subsp. *aureus* wild-type (WT), Δ*endP1*, Δ*endM1* and hybrid strains. 38

Supplementary Figure 25. Comparison of the titers of End produced by wild-type strain of *S. endus* subsp. *aureus* and End-16 produced by Δ*endP3*::*lenG5* (*SPL42*), Δ*endG5*::*lenG5* (*lenG5p*), and Δ*endG5*::*lenG5* (*SPL42*) mutants. 38

Supplementary Figure 26. Key ^{1}H–^{1}H COSY, HMBC, and NOESY correlations of End-2, End-3, End-4, End-5, Len-10. Len-11, End-16. 39

Supplementary Figure 27. Gating strategy for flow cytometry. 40

Supplementary Figure 28. Image of spleen tissues from mice in each group. 40

Supplementary Figure 29. H&E staining and Sirius Red staining of liver tissues. 41

NMR spectrum, HRMS spectrum, Optical rotation value, UV spectrum, IR spectrum and ECD spectra 42

Supplementary References 81

Supplementary Tables

Supplementary Table 1. Deduced functions of ORFs in the *end* gene cluster.

ORF	Size[a]	Proposed function	ID/SI (%)	Protein homologue and origin
endR1	202	Transcriptional regulator	98/99	Response regulator transcription factor (WP_089509403.1); *Streptomyces* sp. NBS 14/10
endT1	399	ABC transporter ATP-binding protein	56/64	ATP-binding protein (WP_107485799.1); *Streptomyces marincola*
endT2	398	ABC transporter ATP-binding protein	75/80	ATP-binding protein (WP_065968266.1); *Streptomyces sparsogenes*
endR2	246	Transcriptional activator	95/96	NanR1 (AAP42853.1); *Streptomyces nanchangensis*
endR3	253	Transcriptional activator	97/98	NanR2 (AAP42854.1); *Streptomyces nanchangensis*
endA1	2886	Type I PKS (loading module: KS-AT-ACP; module 1: KS-AT-DH-KR*-ACP)	88/90	NanA1 (AAP42855.1); *Streptomyces nanchangensis*
endA2	2227	Type I PKS (module 2: KS-AT-DH-ER-KR-ACP)	91/93	NanA2 (AAP42856.1); *Streptomyces nanchangensis*
endA3	4041	Type I PKS (module 3: KS-AT-DH-KR-ACP; module 4: KS-AT-DH-ER-KR-ACP)	92/93	NanA3 (AAP42857.1); *Streptomyces nanchangensis*
endA4	3927	Type I PKS (module 5: KS-AT-DH-KR*-ACP; module 6: KS-AT-DH-ER-KR-ACP)	85/89	NanA4 (AAP42858.1); *Streptomyces nanchangensis*
endA5	3977	Type I PKS (module 7: KS-AT-DH-KR-ACP; module 8: KS-AT-DH-ER-KR-ACP)	82/87	NanA5 (AAP42859.1); *Streptomyces nanchangensis*
endA6	1673	Type I PKS (module 9: KS-AT-KR*-ACP)	84/88	NanA6 (AAP42860.1); *Streptomyces nanchangensis*
endP3	399	Cytochrome P450	71/82	Cytochrome P450 (WP_089509390.1); *Streptomyces* sp. NBS 14/10
endG5	434	Glycosyltransferase	65/79	NanG5 (AAP42861.1); *Streptomyces nanchangensis*
endM1	310	Methyltransferase	88/95	NanM (AAP42862.1); *Streptomyces nanchangensis*
endG4	351	NAD(P)-dependent dehydratase/epimerase/reductase	85/87	NAD(P)-dependent oxidoreductase

ORF	Size[a]	Proposed function	ID/SI (%)	Protein homologue and origin
				(WP_089509388.1); *Streptomyces* sp. NBS 14/10
endG3	434	Dehydratase	96/97	Lipopolysaccharide biosynthesis protein RfbH (WP_089509387.1); *Streptomyces* sp. NBS 14/10
endG2	330	dTDP-glucose-4,6-dehydratase	90/92	dTDP-glucose 4,6-dehydratase (WP_089509386.1); *Streptomyces* sp. NBS 14/10
endG1	270	Glucose-1-phosphate thymidylyltransferase	95/96	Glucose-1-phosphate thymidylyltransferase RfbA (WP_065957879.1); *Streptomyces sparsogenes*
endG9	341	Glycosyltransferase	45/56	Glycosyltransferase (PJN03927.1); *Streptomyces* sp. CB01201
endA7	1663	Type I PKS (module 10: KS-AT-KR-ACP)	84/89	NanA7 (AAP42867.1); *Streptomyces nanchangensis*
endE	281	Thioesterase	68/78	NanE (AAP42868.1); *Streptomyces nanchangensis*
endA10	104	ACP (acyl carrier protein) domain	75/84	Acyl carrier protein (WP_014180973.1); *Streptomyces*
endO	474	Epoxidase	87/92	NanO (AAP42870.1); *Streptomyces nanchangensis*
endI	313	Epoxide hydrolase	73/80	NanI (AAP42871.1); *Streptomyces nanchangensis*
endM2	183	Methyltransferase	64/72	Methyltransferase (GGV44293.1); *Actinomadura cremea*
endP2	429	Cytochrome P450	72/83	Cytochrome P450 (WP_089509381.1); *Streptomyces* sp. NBS 14/10
endA9	796	Type I PKS (module 13: KS)	81/86	NanA9 (AAP42872.1); *Streptomyces nanchangensis*
endA11	2167	Type I PKS (module 14: KS-AT-DH-ER-KR-ACP)	84/89	NanA11 (AAP42873.1); *Streptomyces nanchangensis*
endA8	3448	Type I PKS (module 11: KS-AT-KR-ACP; module 12: KS-AT-DH-KR-ACP)	85/89	NanA8 (AAP42874.1); *Streptomyces nanchangensis*
endP1	398	Cytochrome P450	90/93	NanP (AAP42875.1); *Streptomyces nanchangensis*
endR4	316	Transcriptional suppressor	93/95	NanR4 (AAP42877.1);

ORF	Size[a]	Proposed function	ID/SI (%)	Protein homologue and origin
				Streptomyces nanchangensis
endG6	392	Glycosyltransferase	95/97	Glycosyltransferase (WP_089513423.1); *Streptomyces* sp. NBS 14/10
endG7	581	Glycosyltransferase	97/97	NanG7 (AAP42879.1); *Streptomyces nanchangensis*
endG8	309	Glycosyltransferase	92/97	NanG8 (AAP42880.1); *Streptomyces nanchangensis*

[a] Size in units of amino acids (aa); ID/SI: identity/similarity.

Supplementary Table 2. Deduced functions of ORFs in the *len* gene cluster.

ORF	Size[a]	Proposed function	ID/SI (%)	Protein homologue and origin
lenR1	214	Transcriptional regulator	93/97	Response regulator transcription factor (WP_089509403.1); *Streptomyces* sp. NBS 14/10
lenT1	399	ABC transporter ATP-binding protein	57/64	ATP-binding protein (WP_107485799.1); *Streptomyces marincola*
lenT2	367	ABC transporter ATP-binding protein	78/82	ATP-binding protein (WP_065968266.1); *Streptomyces sparsogenes*
lenR2	206	Transcriptional activator	91/94	NanR1 (AAP42853.1); *Streptomyces nanchangensis*
lenR3	254	Transcriptional activator	90/94	NanR2 (AAP42854.1); *Streptomyces nanchangensis*
lenA1	2878	Type I PKS (loading module: KS-AT-ACP; module 1: KS-AT-DH-KR*-ACP)	80/84	NanA1 (AAP42855.1); *Streptomyces nanchangensis*
lenA2	2237	Type I PKS (module 2: KS-AT-DH-ER-KR-ACP)	86/90	NanA2 (AAP42856.1); *Streptomyces nanchangensis*
lenA3	4001	Type I PKS (module 3: KS-AT-DH-KR-ACP; module 4: KS-AT-DH-ER-KR-ACP)	86/89	NanA3 (AAP42857.1); *Streptomyces nanchangensis*
lenA4	3892	Type I PKS (module 5: KS-AT-DH*-KR*-ACP; module 6: KS-AT-DH-ER-KR-ACP)	78/85	NanA4 (AAP42858.1); *Streptomyces nanchangensis*
lenA5	3950	Type I PKS (module 7: KS-AT-DH-KR-ACP; module 8: KS-AT-DH-ER-KR-ACP)	82/87	NanA5 (AAP42859.1); *Streptomyces nanchangensis*

ORF	Size[a]	Proposed function	ID/SI (%)	Protein homologue and origin
lenA6	1648	Type I PKS (module 9: KS-AT-KR*-ACP)	70/77	NanA6 (AAP42860.1); *Streptomyces nanchangensis*
lenM1	308	Methyltransferase	85/92	NanM (AAP42862.1); *Streptomyces nanchangensis*
lenG4	332	NAD(P)-dependent dehydratase/epimerase/reductase	82/85	NAD(P)-dependent oxidoreductase (WP_089509388.1); *Streptomyces* sp. NBS 14/10
lenG3	434	Dehydratase	94/98	Lipopolysaccharide biosynthesis protein RfbH (WP_014180978.1); *Streptomyces*
lenG2	330	dTDP-glucose-4,6-dehydratase	92/94	dTDP-glucose 4,6-dehydratase (WP_014180977.1); *Streptomyces*
lenG1	270	Glucose-1-phosphate thymidylyltransferase	86/93	Glucose-1-phosphate thymidylyltransferase RfbA (WP_043492200.1); *Streptomyces*
lenG5	423	Glycosyltransferase	59/73	NanG5 (AAP42861.1); *Streptomyces nanchangensis*
lenA7	1658	Type I PKS (module 10: KS-AT-KR-ACP)	70/77	NanA7 (AAP42867.1); *Streptomyces nanchangensis*
lenE	293	Thioesterase	72/80	NanE (AAP42868.1); *Streptomyces nanchangensis*
lenA10	104	ACP (acyl carrier protein) domain	72/87	Acyl carrier protein (WP_065957886.1); *Streptomyces sparsogenes*
lenO	478	Epoxidase	83/89	NanO (AAP42870.1); *Streptomyces nanchangensis*
lenI	302	Epoxide hydrolase	72/83	NanI (AAP42871.1); *Streptomyces nanchangensis*
lenP2	426	Cytochrome P450	72/82	Cytochrome P450 (WP_089509381.1); *Streptomyces* sp. NBS 14/10
lenA9	785	Type I PKS (module 13: KS)	81/85	NanA9 (AAP42872.1); *Streptomyces nanchangensis*
lenA11	2167	Type I PKS (module 14: KS-AT-DH-ER-KR-ACP)	83/87	NanA11 (AAP42873.1); *Streptomyces nanchangensis*
lenA8	3742	Type I PKS (module 11: KS-AT-KR-ACP; module 12: KS-AT-DH-ER*-KR-ACP)	85/89	NanA8 (AAP42874.1); *Streptomyces nanchangensis*
lenP1	383	Cytochrome P450	87/92	NanP (AAP42875.1); *Streptomyces nanchangensis*

ORF	Size[a]	Proposed function	ID/SI (%)	Protein homologue and origin
lenR4	336	LacI family transcriptional regulator	93/95	LacI family DNA-binding transcriptional regulator (WP_014180966.1); *Streptomyces*
lenR5	316	AraC family transcriptional regulator	90/93	AraC family transcriptional regulator (WP_014180965.1); *Streptomyces*
lenG6	394	Glycosyltransferase	92/95	Glycosyltransferase (WP_014180964.1); *Streptomyces*
lenG7	559	Glycosyltransferase	95/97	Glycosyltransferase (NUS85990.1); *Streptomyces* sp.
lenG8	371	Glycosyltransferase	74/83	NanG8 (AAP42880.1); *Streptomyces nanchangensis*

[a] Size in units of amino acids (aa); ID/SI: identity/similarity.

Supplementary Table 3. Strains used in this study.

Strains	Characteristics	Reference
S. cerevisiae CEN.PK2-1D	Host for general cloning, *MATα, ura3–52, trp1–289, leu2-3,112, his3Δ1, MAL2–8C, SUC2*	Euroscarf
E. coli DH10B	Host for general cloning, *E. coli D F⁻mcrA Δ(mrr-hsdRMS-mcrBC) Φ80dlacZ ΔM15 ΔlacX74 endA1 recA1 deoR Δ(ara,leu)7697 araD139 galU galK nupG rpsL λ⁻*	Invitrogen
E. coli ET12567/pUZ8002	Donor strain for conjugation between *E. coli* and *Streptomyces*, *dam-13::Tn9 dcm-6 hsdM Cml*^{R}, carrying helper plasmid pUZ8002	[1]
E. coli ET12567/pUB307	Donor strain for conjugation between *E. coli* and *Streptomyces*, *dam-13::Tn9 dcm-6 hsdM Cml*^{R}, carrying helper plasmid pUB307	[2]
E. coli GB2005	DH10B, *fhuA::IS2, ΔybcC, ΔrecET*	[3]
E. coli GB05-red	DH10B, *fhuA::IS2, ΔybcC, ΔrecET, araC-BAD-redγβα-recA*	[3]
E. coli GB05-dir	DH10B, *fhuA::IS2, ΔybcC, ΔrecET, araC-BAD-redγ-recETA*	[3]
Streptomyces albus J1074	Isoleucin and valine auxotrophic derivative of *Streptomyce albus* G laking SalI-restriction activity	[4]
S. albus J1074 pBAC*endBGC*	*S. albus* J1074 transfromed with pBAC*endBGC*	This work
Streptomyces endus subsp. *aureus*	Endusamycin producing strain, wild type	ATCC
Δ*endP1*	*endP1* gene deletion mutant derived from *S. endus* subsp. *aureus*	This work

Strains	Characteristics	Reference
Δ*endP2*	*endP2* gene deletion mutant derived from *S. endus* subsp. *aureus*	This work
Δ*endP3*	*endP3* gene deletion mutant derived from *S. endus* subsp. *aureus*	This work
Δ*endM1*	*endM1* gene deletion mutant derived from *S. endus* subsp. *aureus*	This work
Δ*endM2*	*endM2* gene deletion mutant derived from *S. endus* subsp. *aureus*	This work
Δ*endG5*	*endG5* gene deletion mutant derived from *S. endus* subsp. *aureus*	This work
Δ*endG6*	*endG6* gene deletion mutant derived from *S. endus* subsp. *aureus*	This work
Δ*endG7*	*endG7* gene deletion mutant derived from *S. endus* subsp. *aureus*	This work
Δ*endG8*	*endG8* gene deletion mutant derived from *S. endus* subsp. *aureus*	This work
Δ*endG9*	*endG9* gene deletion mutant derived from *S. endus* subsp. *aureus*	This work
Δ*endP1*::*endP1*	Δ*endP1* mutant complemented with the *endP1* gene under the control of *ermEp** promoter	This work
Δ*endP3*::*endP3*	Δ*endP3* mutant complemented with the *endP3* gene under the control of *ermEp** promoter	This work
Δ*endM1*::*endM1*	Δ*endM1* mutant complemented with the *endM1* gene under the control of *ermEp** promoter	This work
Δ*endG5*::*endG5*	Δ*endG5* mutant complemented with the *endG5* gene under the control of *ermEp** promoter	This work
Streptomyces hygroscopicus A-130	Lenoremycin producing strain, wild type	ATCC
Δ*lenP1*	*lenP1* gene deletion mutant derived from *S. hygroscopicus* A-130	This work
Δ*lenP2*	*lenP2* gene deletion mutant derived from *S. hygroscopicus* A-130	This work
Δ*lenM1*	*lenM1* gene deletion mutant derived from *S. hygroscopicus* A-130	This work
Δ*lenG5*	*lenG5* gene deletion mutant derived from *S. hygroscopicus* A-130	This work
Δ*lenG6*	*lenG6* gene deletion mutant derived from *S. hygroscopicus* A-130	This work
Δ*lenG7*	*lenG7* gene deletion mutant derived from *S. hygroscopicus* A-130	This work
Δ*lenG8*	*lenG8* gene deletion mutant derived from *S. hygroscopicus* A-130	This work

Strains	Characteristics	Reference
Δ*lenG5*::*lenG5* (*lenG5p*)	Δ*lenG5* mutant transfromed with pYL1	This work
Δ*lenG5*::*lenG5* (*ermEp**)	Δ*lenG5* mutant transfromed with pYL2	This work
Δ*lenG5*::*lenG5* (*SPL42*)	Δ*lenG5* mutant transfromed with pYL3	This work
S. endus subsp. *aureus* pGUS	*S. endus* subsp. *aureus* transfromed with pGUS	This work
S. endus subsp. *aureus* pGUS-*lenG5p*	*S. endus* subsp. *aureus* transfromed with pGUS-*lenG5p*	This work
S. endus subsp. *aureus* pGUS-*endP3p*	*S. endus* subsp. *aureus* transfromed with pGUS-*endP3p*	This work
S. endus subsp. *aureus* pGUS-*endM1p*	*S. endus* subsp. *aureus* transfromed with pGUS-*endM1p*	This work
S. endus subsp. *aureus* pLH2	*S. endus* subsp. *aureus* transfromed with pLH2	This work
S. endus subsp. *aureus* pLH5	*S. endus* subsp. *aureus* transfromed with pLH5	This work
S. endus subsp. *aureus* pLH8	*S. endus* subsp. *aureus* transfromed with pLH8	This work
S. endus subsp. *aureus* pLH9	*S. endus* subsp. *aureus* transfromed with pLH9	This work
S. endus subsp. *aureus* pLH10	*S. endus* subsp. *aureus* transfromed with pLH10	This work
S. endus subsp. *aureus* pLH18	*S. endus* subsp. *aureus* transfromed with pLH18	This work
S. endus subsp. *aureus* WT::*lenG5* (*lenG5p*)	*S. endus* subsp. *aureus* wild-type transfromed with pYL1	This work
S. endus subsp. *aureus* WT::*lenG5* (*ermEp**)	*S. endus* subsp. *aureus* wild-type transfromed with pYL2	This work
S. endus subsp. *aureus* WT::*lenG5* (*SPL42*)	*S. endus* subsp. *aureus* wild-type transfromed with pYL3	This work
Δ*endP1*::*lenG5* (*lenG5p*)	Δ*endP1* mutant transfromed with pYL1	This work
Δ*endP1*::*lenG5* (*ermEp**)	Δ*endP1* mutant transfromed with pYL2	This work
Δ*endP1*::*lenG5* (*SPL42*)	Δ*endP1* mutant transfromed with pYL3	This work
Δ*endP3*::*lenG5* (*lenG5p*)	Δ*endP3* mutant transfromed with pYL1	This work
Δ*endP3*::*lenG5* (*ermEp**)	Δ*endP3* mutant transfromed with pYL2	This work
Δ*endP3*::*lenG5* (*SPL42*)	Δ*endP3* mutant transfromed with pYL3	This work
Δ*endM1*::*lenG5* (*lenG5p*)	Δ*endM1* mutant transfromed with pYL1	This work
Δ*endM1*::*lenG5* (*ermEp**)	Δ*endM1* mutant transfromed with pYL2	This work
Δ*endM1*::*lenG5* (*SPL42*)	Δ*endM1* mutant transfromed with pYL3	This work
Δ*endG5*::*lenG5* (*lenG5p*)	Δ*endG5* mutant transfromed with pYL1	This work
Δ*endG5*::*lenG5* (*ermEp**)	Δ*endG5* mutant transfromed with pYL2	This work
Δ*endG5*::*lenG5* (*SPL42*)	Δ*endG5* mutant transfromed with pYL3	This work

Supplementary Table 4. Plasmids used in this study.

Plasmids	Characteristics	Reference
pYH7	*Apr*^{R}, *Amp*^{R}, *E. coli-Streptomyces* shuttle vector for gene inactivation	[5]
pIB139	*Apr*^{R}, *E. coli-Streptomyces* shuttle vector for gene complementation and heterologous expression	[6]
p15A-cm-ccdB	*Cml*^{R}, PCR template to generate a linear vector for direct cloning	[7]

Plasmids	Characteristics	Reference
pBeloBAC11	*Cml*^{R}, PCR template to generate a linear vector for direct cloning	[8]
pR6K-oriT-phiC31	*Apr*^{R}, Suicide plasmid to release a conjugation-site-specific-integration cassette to modify a cloning vector (PCR free recombineering); this contains *oriT*, the phiC31 integrase gene (*int*) and its attachment site	[8]
pSC101-BAD-ETgA-tet	*Tet*^{R}, RecET expression plasmid for linear plus linear homologous recombination; this encodes the arabinose-inducible *ETγA* operon (full-length *recE*, *recT*, *redγ* and *recA*)	[3]
pBAC*endBGC*	*Apr*^{R}, *Cml*^{R}, a BAC plasmid containing the *end* biosynthetic cluster	This work
pYH7-*endP1*	pYH7 derivative for deletion of *endP1*	This work
pYH7-*endP2*	pYH7 derivative for deletion of *endP2*	This work
pYH7-*endP3*	pYH7 derivative for deletion of *endP3*	This work
pYH7-*endM1*	pYH7 derivative for deletion of *endM1*	This work
pYH7-*endM2*	pYH7 derivative for deletion of *endM2*	This work
pYH7-*endG5*	pYH7 derivative for deletion of *endG5*	This work
pYH7-*endG6*	pYH7 derivative for deletion of *endG6*	This work
pYH7-*endG7*	pYH7 derivative for deletion of *endG7*	This work
pYH7-*endG8*	pYH7 derivative for deletion of *endG8*	This work
pYH7-*endG9*	pYH7 derivative for deletion of *endG9*	This work
pYH7-*lenP1*	pYH7 derivative for deletion of *lenP1*	This work
pYH7-*lenP2*	pYH7 derivative for deletion of *lenP2*	This work
pYH7-*lenM1*	pYH7 derivative for deletion of *lenM1*	This work
pYH7-*lenG5*	pYH7 derivative for deletion of *lenG5*	This work
pYH7-*lenG6*	pYH7 derivative for deletion of *lenG6*	This work
pYH7-*lenG7*	pYH7 derivative for deletion of *lenG7*	This work
pYH7-*lenG8*	pYH7 derivative for deletion of *lenG8*	This work
pIB139-*endP1*	pIB139 derivative for *S. endus* subsp. *aureus* Δ*endP1* complementation	This work
pIB139-*endP3*	pIB139 derivative for *S. endus* subsp. *aureus* Δ*endP3* complementation	This work
pIB139-*endM1*	pIB139 derivative for *S. endus* subsp. *aureus* Δ*endM1* complementation	This work
pIB139-*endG5*	pIB139 derivative for *S. endus* subsp. *aureus* Δ*endG5* complementation	This work
pGUS	*Apr*^{R}, Promoter probe vector, pSET152 derivative containing *gusA*	[9]
pGUS-*lenG5p*	pGUS derivative containing promoter *lenG5p* for test of promoter strength	This work
pGUS-*endP3p*	pGUS derivative containing promoter *endP3p* for test of promoter strength	This work
pGUS-*endM1p*	pGUS derivative containing promoter *endM1p* for test of promoter strength	This work

Plasmids	Characteristics	Reference
pLH2	pGUS derivative containing promoter *SPL42* for test of promoter strength	[10]
pLH5	pGUS derivative containing promoter *SRL37* for test of promoter strength	[10]
pLH8	pGUS derivative containing promoter *rpsLp-cf* for test of promoter strength	[10]
pLH9	pGUS derivative containing promoter *KasOp-rpsL-CF* for test of promoter strength	[10]
pLH10	pGUS derivative containing promoter *KasOp** for test of promoter strength	[10]
pLH18	pGUS derivative containing promoter *ermEp** for test of promoter strength	[10]
pYL1	pSET152 derivative for heterologous expression of *lenG5* under the control of *lenG5p* promoter	This work
pYL2	pSET152 derivative for heterologous expression of *lenG5* under the control of *ermEp** promoter	This work
pYL3	pSET152 derivative for heterologous expression of *lenG5* under the control of *SPL42* promoter	This work

Supplementary Table 5. Primers used in this study.

**ExoCET cloning**	
p15A-1	GTGGGCGTTTCCCAGCCCGCCCACCGTCACCCCACCTTCTCACCCCCGAGGATGCTCATGCTCCGCCAACCAGGAAGGGCGACGTCGATATCTGGCGAAAATGA
p15A-2	CACCGCTAGCCCCGGCAAACACGCCGCGCTGGAGGCCATGGGCATCGACGAGACCCACCGTCTAGATTAATTAATTTATACCTAGGGATATATTCCGCTT
p15A-3	TCATCCACGCGGCGGCCGGCGGTGTGGGCATGGCCGCCGTGCAGATCGCCCGCCACCTGGGCGCCGAGGTGTACGGCACCGCTAGCCCCGGCAAACA
oriT-phiC31-1	ACGTAGTGCCGGCACGTTAACCGGGCTGCATCCGATGCAAGTGTGTCGCTGTCGATGCAAGTGTGTCGCTGTCGACCTA
oriT-phiC31-2	CCCACCTTCTCACCCCCGAGGATGCTCATGCTCCGCCAACCAGGAAGGGCTTTTACAACGTCGTGACTGGGAAAGGAT
oriT-phiC31-3	GAACCTCTTACGTGCCGATCAACGTCTCATTTTCGCCAGATATCGACGTCACTAGTTACGTAGTGCCGGCACGTTAA
pBeloBAC11-1	GTCTCGAAGTCGAGGTCGCGGGAGGAGGCGCGGTGGGTCTCGTCGATGCCCATGGCCTCCAGCGCGGCGTGTTTGCCGGGGCTAGCTTAATTAATCGACAGCGACACACTTGCAT

pBeloBAC11-2		GCCGGACGGCGGGCGCGGCGGGGGCCTGCGCTCCCGTCTTCGACGCTCCCGCGCCCGCCGACCCGGCGCAAACCGGGCGGCGTGCCAGCTGCATTAATGAAT
**Gene inactivation**		
**Gene**	**Primer**	**Sequence (5' to 3')**
endP1	endP1-LHA-F	CGTTCCGCCACCACCGCCGCCACGTGTCCCGACTTCCCACACTCTGACCACGTTCTGAC
	endP1-LHA-R	GGCGAAAGGGGGATGTGCTGCAAGGCGATTAAGTTGGGGCACCATCCTCCAGCGACAAC
	endP1-RHA-F	GCTCCGGCGGGCCGCTGGCGCGTCCACCGGGACTGATCAAGGCGAATACTTCATATGTGATCGCCACCGAGCACAGCCTG
	endP1-RHA-R	CGTCAGAACGTGGTCAGAGTGTGGGAAGTCGGGACACGTGGCGGCGGTGGTGGCGGAACGGGAC
	endP1-SC-F	GTGGTGGCGGGGGCGTTGTCGCTGGAGGATGGTGCCCCAACTTAATCGCCTTGCAGCAC
	endP1-SC-R	TGACCCCGAAGCAGGGTTATGCAGCGGAAAAGATCCGTCGACCTGCAGGCATGCAAGCTTTGTATTTAGAAAAATAAACA
endP2	endP2-LHA-F	CGGGATACCGGGTCTAGACACGCCTCACCGGGCTTCCGCGCCGCCGCGATGATGTGCTC
	endP2-LHA-R	GATCCGTCGACCTGCAGGCATGCAAGCTTAGGGTCACCAGGTCGCTGTGGAAG
	endP2-RHA-F	GCGTCCACCGGGACTGATCAAGGCGAATACTTCATATGCTACGGCGTGTTCATGGGCAC
	endP2-RHA-R	CGCGGAAGCCCGGTGAGGCGTGTCTAGACCCGGTATCCCGGGGCGCACAC
endP3	endP3-LHA-F	GGCGCCGCCCGCCCGCTCTAGAGACTCCTCCTCAGAATTGTCGCGCGGTC
	endP3-LHA-R	GGCATGCAAGCTTCGCTGCGGGTGCTGTGCGACGAG
	endP3-RHA-F	TGATCAAGGCGAATACTTCATATGGCGCGAGCGTGTTCTTCAGATCGATGATGGTGTAG
	endP3-RHA-R	CAATTCTGAGGAGGAGTCTCTAGAGCGGGCGGGCGGCGCCGCGACGGTGACAC
endM1	endM1-LHA-F	CTCCCGGGAGGACGGGGTCTAGACTCGCTCTCCCACTCGTCGATGTTG
	endM1-LHA-R	GGCATGCAAGCTTGGTGGAAGAGCTCCTGCGCTATG
	endM1-RHA-F	GGCGAATACTTCATATGGTGCCGCCGTGCCGAGCAGAATG
	endM1-RHA-R	ACGAGTGGGAGAGCGAGTCTAGACCCCGTCCTCCCGGGAGGGAAC
endM2	endM2-LHA-F	CATCACGCCTCTAGACTTCGGGCCTTCCGTCGTCGGCCATCCGCACCGGTCTCAG
	endM2-LHA-R	CATGCAAGCTTCCCCCGAGTGCGACTGGAAGATCTGGCTGATGGGTTC

	endM2-RHA-F	GTCCACCGGGACTGATCAAGGCGAATACTTCATATGCCGGCACCGGCAAGCAGCTCTAC
	endM2-RHA-R	ATGGCCGACGACGGAAGGCCCGAAGTCTAGAGGCGTGATGCCCACAGGCGAGAC
endG5	endG5-LHA-F	CTCCCATGAGAGAGAGGGAATTCTCCCCCCTTGAACACCCCTGGTC
	endG5-LHA-R	ATGCAAGCTTTGGCGCTGGTGCGCTCCCATGTG
	endG5-RHA-F	GGCGAATACTTCATATGAGCCCGCTGAGCGAGATCATCAG
	endG5-RHA-R	GGGTGTTCAAGGGGGGAGAATTCCCTCTCTCTCATGGGAGGGAC
endG6	endG6-LHA-F	GGAAGATGGGGCCCGGTCGCCCGATGAGATCACCCTGCGCTGGCCGCGGAGCGGTGCTC
	endG6-LHA-R	CCAGCTGGCGAAAGGGGGATGTGCTGCAAGGCGATTAAGTTGCTCGAAGTCCGAGAGCAGGAGTTC
	endG6-RHA-F	GTCGCTCCGGCGGGCCGCTGGCGCGTCCACCGGGACTGATCAAGGCGAATACTTCATATGCCACGGCCCACACCAGATGGATAC
	endG6-RHA-R	GCGGGAGCACCGCTCCGCGGCCAGCGCAGGGTGATCTCATCGGGCGACCGGGCCCCATC
	endG6-SC-F	CCCCGACCAGCGGGAACTCCTGCTCTCGGACTTCGAGCAACTTAATCGCCTTGCAGCAC
	endG6-SC-R	TGACCCCGAAGCAGGGTTATGCAGCGGAAAAGATCCGTCGACCTGCAGGCATGCAAGCTTTGTATTTAGAAAAATAAACA
endG7	endG7-LHA-F	CGGGCGCGCTCCGTCTAGACTCGTGGTGCGCGGGCCGCGCGGGCGGGGGAGCGAC
	endG7-LHA-R	GGAAAAGATCCGTCGACCTGCAGGCATGCAAGCTTAGGCGGGCCAGACGGCGGTCCTTG
	endG7-RHA-F	CGTCCACCGGGACTGATCAAGGCGAATACTTCATATGTGTGCTGGTCGCCCTGCGGATG
	endG7-RHA-R	GCGCGGCCCGCGCACCACGAGTCTAGACGGAGCGCGCCCGCCCCGTCCTCATC
endG8	endG8-LHA-F	TGGGAAAGGTGTGCTCGTCTAGAGGACGGGGCGGGCGCGCTCCGTCAGTG
	endG8-LHA-R	CGGAAAAGATCCGTCGACCTGCAGGCATGCAAGCTTAAATCCGATTTGCACAGCCATTC
	endG8-RHA-F	CGGGACTGATCAAGGCGAATACTTCATATGCTGATCGCCGTGCGCCTCAACATC
	endG8-RHA-R	GCGCGCCCGCCCCGTCCTCTAGACGAGCACACCTTTCCCACAGTTTC
endG9	endG9-LHA-F	AAAACAGCGTCGATGCGTCTAGAGCCGCATCTCTCACCCGCTATC
	endG9-LHA-R	CATGCAAGCTTCTGGGCGGAGTGCTTGAGCGGTTC
	endG9-RHA-F	CGAATACTTCATATGAGCACGGCGCCGACGCCACGGCATG
	endG9-RHA-R	CGGGTGAGAGATGCGGCTCTAGACGCATCGACGCTGTTTCCCCGAATTC
lenP1	lenP1-LHA-F	GAATACTTCATATGCCGTCAGCGACCAGTCCACAAAC

	lenP1-LHA-R	CGAATCTAGAGAACCACGACGGACACGTCTTCGAG
	lenP1-RHA-F	GTTCTCTAGATTCGCCAGCGCGTAATAGCCAGATG
	lenP1-RHA-R	ATGCAAGCTTGGATGGGCCGGATGGTTCTGAAG
lenP2	lenP2-LHA-F	GAATACTTCATATGCGACCATGACCGCGGGGAAAGTG
	lenP2-LHA-R	GCACTCTAGAGGCGTGGTGATCCGTCGTACATC
	lenP2-RHA-F	CGCCTCTAGAGTGCCGGAGAAGGAGCTGACGTG
	lenP2-RHA-R	ATGCAAGCTTGGCACCACCGGACAGGACTACAC
lenM1	lenM1-LHA-F	GAATACTTCATATGCATGGGCCGACGCCTGTGAGCAG
	lenM1-LHA-R	CCTGTCTAGACTCAGTGACCCAGAGCTCGTTGATG
	lenM1-RHA-F	TGAGTCTAGACAGGTGCTCGACATCCACGACAG
	lenM1-RHA-R	ATGCAAGCTTAGCCGATGTGGGAGAAGATGTAC
lenG5	lenG5-LHA-F	GAATACTTCATATGATCGGTGTGTCGGTTGAGGTCAG
	lenG5-LHA-R	AACCTCTAGAGGCTACCGTCTCTCCTCACCAGTC
	lenG5-RHA-F	AGCCTCTAGAGTGATCTCCTCATGTTCCGCTGTG
	lenG5-RHA-R	ATGCAAGCTTCCACCACCTGTCCCGTCGATAC
lenG6	lenG6-LHA-F	GAATACTTCATATGTCGGGGCGTGCAGGTGACGATTC
	lenG6-LHA-R	GCCATCTAGACGCTGCTGTACGAACGCTCCGTC
	lenG6-RHA-F	AGCGTCTAGATGGCGCGGGCTTCCTCACACATG
	lenG6-RHA-R	ATGCAAGCTTCGCGGCCCACACCAGATGGATAC
lenG7	lenG7-LHA-F	GAATACTTCATATGGAGTGCGGCTCACCGTTGTTGTC
	lenG7-LHA-R	GGTGTCTAGACGGGTCGCCTGGGACAAAATATC
	lenG7-RHA-F	CCCGTCTAGACACCTTCCGTATCCATCTGGTGT
	lenG7-RHA-R	ATGCAAGCTTTGAGCGCGGCCTGCCTGTATCTG
lenG8	lenG8-LHA-F	GAATACTTCATATGAGTGCCCAGATGTCTGCACCAAC
	lenG8-LHA-R	GAGGTCTAGAATCGACGCCTATCTGTGGATCAAG
	lenG8-RHA-F	CGATTCTAGACCTCGGAATTGACGGTGTGTGTC
	lenG8-RHA-R	ATGCAAGCTTTACACCAAGGACCTCCAGATCAC
**Verification of gene inactivation**		
**Gene**	**Primer**	**Sequence (5' to 3')**
endP1	endP1-test-F	GCGGTGAAATACCGAAGAATTC
	endP1-test-R	GAGGCATTGTGTCTCCTTCGGA
endP2	endP2-test-F	CGGAACGGTCCACCTCGAACTG
	endP2-test-R	CTTGGAGCGGTTCGGGTATGAA
endP3	endP3-test-F	TGGCGGCCGTCGAGTTCAAGAAC
	endP3-test-R	GCAAGGAAATCGGGAAATAC
endM1	endM1-test-F	CCGCCTCCACTAAAGCGGATCT
	endM1-test-R	TAGGCGCTGCCCAGATGGATCA
endM2	endM2-test-F	ATCGGCGATCCTCCGCTGAGAC
	endM2-test-R	GGCTGGGCGAAGTGTTCGACCA
endG5	endG5-test-F	TGACCGACCAGGGGTGTTCAAG
	endG5-test-R	CTGGAGATAGTCCAGACAGATG
endG6	endG6-test-F	GGGCCAGACGGCGGTCCTTGAC

	endG6-test-R	GGCGTGCGACATACGGAAGATG
endG7	endG7-test-F	TCCGTATGTCGCACGCCTTATG
	endG7-test-R	CGGCAGGGTCCACTTCATCATC
endG8	endG8-test-F	GGAACCGGCGGCACAACGTCTC
	endG8-test-R	CGGCCCATGAACACCCGCTGTG
endG9	endG9-test-F	GGACGATAGCGGGTGAGAGATG
	endG9-test-R	GCCGCTTCCTGGCGAGCTTCAC
lenP1	lenP1-test-F	CCGCCACCAGATTCCACAAGTC
	lenP1-test-R	TCGTACCTGTCCACGCTTGAATC
lenP2	lenP2-test-F	CGAAGATGACACCAGCAGGTGAC
	lenP2-test-R	AGGGCACGGCCTATCTCGAACTG
lenM1	lenM1-test-F	TACAGGTGCTGCTGTGGCGTTAC
	lenM1-test-R	GAGCAGATGTGCCGTCCCAGATAC
lenG5	lenG5-test-F	GAACGGACAGCGGATAGTAGATC
	lenG5-test-R	AACAGCGGGTCATGTCGGAACTC
lenG6	lenG6-test-F	CCTGCGGCTGCTTCAGAACCATC
	lenG6-test-R	CGGCTTACATTCGTCAGGTCATC
lenG7	lenG7-test-F	CGCGATGACCTGACGAATGTAAG
	lenG7-test-R	TCGACGCCTATCTGTGGATCAAG
lenG8	lenG8-test-F	TGTCCACCTTCCGTATCCATCTG
	lenG8-test-R	CGCGAGGACACAAGCGTGAATTG
**Gene complementation**		
**Gene**	**Primer**	**Sequence (5' to 3')**
endP1	endP1-hb-F	AGGATCCACATATGGTGTCTCCTTCGGAAGCCACGTC
	endP1-hb-R	ATCCTCTAGAGTCAGAATGTGCAGCGCAGTTCTTTG
endP3	endP3-hb-F	AGGATCCACATATGATGCAGAACACCCCGATCAACT
	endP3-hb-R	ATCCTCTAGACTCACCACGCGACCGGCAGCTGATG
endM1	endM1-hb-F	AGGATCCACATATGATGGTGCAGGGTTTCCAGGCCAGT
	endM1-hb-R	ATCCTCTAGACTACGGAAGGTCGCTGCCGGTCTTC
endG5	endG5-hb-F	AGGATCCACATATGATGGAGCGCCGGCCCGGAAAAC
	endG5-hb-R	ATCCTCTAGAGGATCAAGCGGAGCGGTATTC
**Test of promoter strength**		
**promoter**	**Primer**	**Sequence (5' to 3')**
*lenG5*p	lenG5p-gus-F	CGACTCTAGAAGAACGGACAGCGGATAGTAGAT
	lenG5p-gus-R	CAGTACAGCATATGGGCTACCGTCTCTCCTCACCAGTC
endP3p	endP3p-gus-F	CGACTCTAGATTCAAGGAGCTGGGTTTCGACTC
	endP3p-gus-R	CTGAGCAGCATATGGACTCCTCCTCAGAATTGTC
endM1p	endM1p-gus-F	CGACTCTAGATTCAGATAGCTCCACGGAAGCAG
	endM1p-gus-R	CTAGCTAGCATATGCTCGCTCTCCCACTCGTCGATGTT
**heterologous expression of *lenG5***		
**Plasmid**	**Primer**	**Sequence (5' to 3')**
pYL1	lenG5-XbaI-F	CGACTCTAGAAGAACGGACAGCGGATAGTAGAT

	lenG5-KpnI-R	TATCGGTACCCGGAAGAGATCTTCGACCTGAT
pYL2	lenG5-NdeI-F	AGGATCCACATATGCGCGTCCTGTTCGTCACATTC
	lenG5-KpnI-R	TATCGGTACCCGGAAGAGATCTTCGACCTGAT
pYL3	lenG5-NdeI-F	AGGATCCACATATGCGCGTCCTGTTCGTCACATTC
	lenG5-KpnI-R	TATCGGTACCCGGAAGAGATCTTCGACCTGAT

Supplementary Table 6. The ^{1}H (600 MHz) and ^{13}C NMR (150 MHz) data for End-2 and End-3 in $CDCl_3$ (δ in ppm, J in Hz).

Position	End-2		Position	End-3	
	δ_C	δ_H		δ_C	δ_H
1	183.6	-	1	183.2	-
2	40.1	2.54, m	2	39.6	2.55, m
3	41.7	1.06, m	3	41.4	1.08, m
		1.76, m			1.75, m
4	37.7	3.35, m	4	37.5	3.37, m
5	205.9	-	5	206.0	-
6	133.8	-	6	134.2	-
7	145.0	6.54, d (10.3)	7	144.5	6.48, d (10.1)
8	36.3	2.71, tq (10.3, 6.7)	8	36.2	2.68, m
9	69.9	4.16, dd (10.3, 1.8)	9	70.2	4.13, dd (10.2, 1.8)
10	36.9	1.72, m	10	36.7	1.72, m
11	70.7	3.85, m	11	70.5	3.86, m
12	34.1	1.68, m	12	34.0	1.77, m
		1.91, m			1.89, m
13	107.0	-	13	104.3	-
14	39.9	1.66, m	14	47.6	1.88, m
		1.98, m			2.25, dd (12.3, 7.4)
15	32.8	1.71, m	15	78.6	4.61, t (7.4)
		1.97, m			
16	87.3	-	16	86.4	-
17	81.0	3.23, dd (11.6, 2.7)	17	78.9	3.44, m
18	18.0	1.49, m	18	19.6	1.61, m
19	27.4	1.64, m	19	27.4	1.63, m
20	30.1	1.95, m	20	30.3	1.97, m
21	110.8	-	21	111.1	-
22	35.3	2.58, m	22	35.2	2.63, m
23	29.8	1.34, m	23	30.1	1.36, m
		2.43, m			2.38, m
24	79.2	4.36, dd (11.2, 5.1, 2.0)	24	79.2	4.37, dd (11.3, 5.0, 2.0)
25	73.8	3.87, m	25	73.9	3.88, m

26	33.0	1.28, m	26	33.1	1.29, m
27	37.2	1.41, m	27	37.1	1.39, m
					1.47, m
28	35.8	1.47, m	28	35.7	1.49, m
29	98.8	-	29	98.8	-
30	66.5	3.39, d (12.3)	30	66.5	3.39, d (12.0)
		3.96, d (12.3)			3.93, d (12.0)
31	19.4	1.03, d (6.8)	31	19.3	1.04, m
32	14.6	1.10, d (6.3)	32	14.8	1.09, m
33	11.5	1.76, s	33	11.5	1.77, s
34	17.1	1.12, d (6.6)	34	17.2	1.08, m
35	10.5	0.80, d (7.0)	35	10.4	0.79, d (6.4)
36	27.1	1.50, s	36	25.5	1.50, s
37	13.3	1.00, d (6.9)	37	13.3	1.03, m
38	15.1	0.96, d (7.1)	38	15.1	0.97, d (7.1)
39	18.2	0.86, d (6.5)	39	18.2	0.87, d (6.5)
40	17.0	0.90, d (6.1)	40	17.0	0.89, d (6.1)

"m" shows multiplet or overlapped with other signals.

Supplementary Table 7. The ^{1}H (600 MHz) and ^{13}C NMR (150 MHz) data for End-4 and End-5 in $CDCl_3$ (δ in ppm, J in Hz).

Position	End-4		Position	End-5	
	δ_C	δ_H		δ_C	δ_H
1	179.1	-	1	183.5	-
2	40.3	2.45, m	2	39.8	2.54, m
3	41.1	1.09, m	3	41.6	1.08, m
		1.78, m			1.74, m
4	37.0	3.32, dq (8.8, 6.3)	4	37.6	3.37, m
5	205.6	-	5	205.9	-
6	134.9	-	6	134.0	-
7	145.1	6.43, d (9.8)	7	144.6	6.49, d (10.2)
8	35.5	2.78, m	8	36.2	2.69, tq (10.2, 6.6)
9	70.4	4.13, dd (11.0, 1.7)	9	70.1	4.14, dd (10.2, 1.9)
10	36.3	1.78, m	10	36.7	1.71, m
11	70.1	3.89, m	11	70.4	3.85, m
12	34.2	1.78, m	12	33.9	1.75, m
		1.89, m			1.88, dd (14.8, 3.8)
13	104.7	-	13	104.0	-
14	45.8	1.80, m	14	45.8	1.80, dd (12.4, 10.8)
		2.32, dd (12.4, 7.6)			2.28, dd (12.4, 7.2)
15	84.8	4.43, dd (10.6, 7.6)	15	85.0	4.46, dd (10.8, 7.2)

16	86.6	-	16	86.5	-
17	78.6	3.49, dd (9.0, 1.8)	17	78.9	3.53, dd (11.0, 2.5)
18	19.2	1.55, m	18	19.2	1.52, m
					1.56, m
19	27.9	1.58, m	19	27.5	1.56, m
20	30.3	1.93, m	20	30.3	1.94, hd (6.9, 3.1)
21	110.5	-	21	110.9	-
22	35.5	2.61, m	22	35.2	2.60, h (7.0)
23	29.7	1.29, m	23	29.7	1.29, m
		2.40, m			2.37, m
24	78.6	4.36, dd (11.4, 5.0, 2.0)	24	79.1	4.34, dd (11.7, 5.2, 1.9)
25	74.3	3.93, m	25	73.8	3.87, dd (10.6, 2.0)
26	33.1	1.31, m	26	33.0	1.29, m
27	37.3	1.38, m	27	37.1	1.38, m
					1.48, m
28	39.7	1.57, m	28	35.7	1.49, m
29	98.5	-	29	98.8	-
30	27.6	1.49, s	30	66.4	3.38, d (12.3)
					3.94, d (12.3)
31	19.3	1.05, d (6.8)	31	19.3	1.03, d (6.8)
32	15.0	1.11, d (6.3)	32	14.7	1.09, d (6.4)
33	11.5	1.77, s	33	11.5	1.76, s
34	17.4	1.01, d (6.7)	34	17.2	1.12, d (6.6)
35	10.6	0.82, d (6.9)	35	10.4	0.78, d (7.0)
36	26.1	1.57, s	36	25.2	1.53, s
37	14.0	1.06, s	37	13.4	1.00, d (6.9)
38	15.5	0.95, d (7.0)	38	15.1	0.95, d (7.1)
39	18.1	0.87, d (6.4)	39	18.2	0.85, d (6.5)
40	16.8	0.91, d (6.6)	40	17.0	0.89, d (5.9)
1′	101.7	4.36, m	1′	101.8	4.37, dd (9.2, 2.1)
2′	30.7	1.47, m	2′	31.1	1.53, m
		1.84, m			1.79, m
3′	27.0	1.31, m	3′	31.2	1.45, m
		2.40, m			2.05, m
4′	80.2	2.82, dd (10.5, 8.8)	4′	71.4	3.26, m
5′	74.8	3.30, dq (8.8, 6.1)	5′	76.0	3.26, m
6′	18.5	1.27, d (6.1)	6′	18.2	1.28, s
4′-OCH_3	57.0	3.36, s			

“m” shows multiplet or overlapped with other signals.

Supplementary Table 8. The ^{1}H (600 MHz) and ^{13}C NMR (150 MHz) data for Len-10 and Len-11 in $CDCl_3$ (δ in ppm, *J* in Hz).

Position	Len-10		Position	Len-11	
	δ_C	δ_H		δ_C	δ_H
1	178.4	-	1	182.6	-
2	38.2	2.55, m	2	38.5	2.53, m
3	40.0	1.12, m	3	40.8	1.02, m
		1.91, m			1.83, m
4	37.3	3.22, m	4	37.6	3.48, m
5	205.5	-	5	207.0	-
6	134.7	-	6	134.8	-
7	145.5	6.73, d (10.1)	7	145.6	7.05, d (10.3)
8	41.4	2.56, m	8	41.5	2.55, m
9	67.9	3.90, m	9	67.6	3.89, t (10.7)
10	34.5	1.19, m	10	35.9	1.25, m
		1.99, m			1.87, m
11	71.2	3.98, m	11	74.4	3.78, s
12	39.6	1.86, m	12	39.1	1.90, m
13	109.4	-	13	109.0	-
14	36.7	1.88, m	14	36.5	1.80, m
		2.04, m			1.98, m
15	32.9	1.71, m	15	32.6	1.71, m
		1.84, m			1.95, m
16	87.3	-	16	87.5	-
17	80.9	3.25, dd (11.8, 2.8)	17	81.4	3.28, dd (11.9, 2.7)
18	17.4	1.54, m	18	17.1	1.74, m
		1.84, m			
19	28.1	1.60, m	19	27.5	1.63, m
20	30.4	1.94, m	20	30.2	1.97, m
21	110.5	-	21	111.2	-
22	35.4	2.62, m	22	35.0	2.60, m
23	29.7	1.28, m	23	29.7	1.33, m
		2.45, dt (11.5, 6.6)			2.43, dt (11.7, 6.5)
24	78.5	4.35, m	24	79.4	4.37, m
25	74.3	3.94, m	25	73.8	3.85, m
26	33.3	1.30, m	26	33.0	1.26, m
27	37.8	1.39, m	27	37.0	1.38, m
					1.45, m
28	39.4	1.56, m	28	35.8	1.45, m
29	98.5	-	29	98.8	-
30	27.4	1.51, s	30	65.9	3.34, d (12.4)
					3.99, d (12.4)

31	19.5	1.12, d (6.8)	31	19.9	1.08, d (6.9)
32	14.9	1.08, m	32	14.9	1.04, d (6.6)
33	11.6	1.76, s	33	11.6	1.74, s
34	17.3	1.06, m	34	17.3	1.14, d (6.5)
35	13.8	1.06, m	35	13.6	1.10, d (7.4)
36	26.8	1.53, s	36	27.3	1.52, s
37	13.4	1.11, d (6.8)	37	13.4	1.03, d (6.6)
38	14.9	0.95, d (7.1)	38	15.1	0.96, d (7.1)
39	18.1	0.87, d (6.6)	39	18.3	0.86, d (6.4)
40	16.9	0.92, d (6.8)	40	17.0	0.89, d (5.5)
1′	99.4	4.33, dd (9.2, 1.8)	1′	102.2	4.34, m
2′	29.9	1.50, m	2′	31.1	1.48, m
		1.94, m			1.85, m
3′	27.3	1.14, m	3′	31.1	1.35, m
		2.14, m			
4′	80.7	2.76, dd (9.9, 9.2)	4′	71.0	3.25, m
5′	74.7	3.18, m	5′	76.8	3.20, dq (7.2, 6.7)
6′	18.4	1.21, d (6.3)	6′	18.1	1.23, d (6.7)
4′-OCH_3	57.0	3.32, s			

“m” shows multiplet or overlapped with other signals.

Supplementary Table 9. The ^{1}H (600 MHz) and ^{13}C NMR (150 MHz) data for End-16 in $CDCl_3$ (δ in ppm, J in Hz).

Position	**End-16**	
	δ_C	δ_H
1	182.2	-
2	38.8	2.53, m
3	41.1	1.10, m
		1.82, m
4	38.3	3.44, m
5	206.9	-
6	134.4	-
7	145.1	6.98, d (10.2)
8	37.1	2.70, m
9	70.5	3.96, m
10	34.1	1.64, m
11	77.1	3.72, s
12	34.3	1.85, d (14.0)
		2.00, m
13	106.7	-
14	39.8	1.66, m

		1.97, m
15	33.1	1.69, m
		1.96, m
16	87.8	-
17	80.8	3.23, m
18	17.8	1.55, m
19	27.5	1.62, m
20	30.3	1.95, m
21	110.8	-
22	35.1	2.58, h (7.0)
23	29.7	1.31, dd (11.8, 5.1)
		2.38, td (11.8, 7.0)
24	79.1	4.36, dd (11.8, 5.1, 2.0)
25	73.6	3.92, m
26	33.0	1.29, m
27	37.3	1.42, m
28	35.6	1.48, m
29	98.5	-
30	66.0	3.40, d (12.4)
		3.97, d (12.4)
31	19.7	1.10, d (7.1)
32	15.0	1.05, d (6.6)
33	11.7	1.76, s
34	17.7	1.14, d (6.6)
35	11.6	0.81, d (7.2)
36	26.8	1.49, s
37	13.5	1.05, d (6.7)
38	15.1	0.95, d (7.0)
39	18.1	0.85, d (6.5)
40	17.1	0.91, d (5.5)
1′	102.0	4.41, dd (11.8, 5.1)
2′	30.3	1.45, m
		1.51, m
3′	27.0	1.19, m
		2.17, m
4′	80.2	2.71, m
5′	75.0	3.22, m
6′	18.5	1.20, d (6.1)
4′-OCH_3	56.9	3.30, s

“m” shows multiplet or overlapped with other signals.

Supplementary Table 10. Primary and secondary antibodies.

Antigen	Species source	Dilution (WB)	Dilution (IHC)	Supplier
N-cadherin	Rabbit	1:1000	1:200	CST, USA, Cat. #13116
E-cadherin	Rabbit	1:1000	/	CST, USA, Cat. #3195
Snail	Rabbit	1:1000	/	CST, USA, Cat. #3879
CDK1	Mouse	1:1000	/	Abcam, UK, Cat. #ab18
CDK2	Rabbit	1:1000	/	Proteintech Inc, China, Cat. #10122-1-AP
CDK6	Rabbit	1:1000	/	CST, USA, Cat. #13331
Cyclin B1	Rabbit	1:1000	/	Abcam, UK, Cat. #ab32053
Cyclin D1	Rabbit	1:1000	/	CST, USA, Cat. #2922
Cyclin E	Rabbit	1:1000	/	Abcam, UK, Cat. #ab33911
GAPDH	Mouse	1:5000	/	Proteintech Inc, China, Cat. #0004-1-Ig
Goat Anti-mouse IgG (H+L) HRP	Goat	1:5000		Sungene Biotech, China, Cat. #LK2003
Goat Anti-rabbit IgG (H+L) HRP	Goat	1:5000		Sungene Biotech, China, Cat. #LK2001

Supplementary Figures

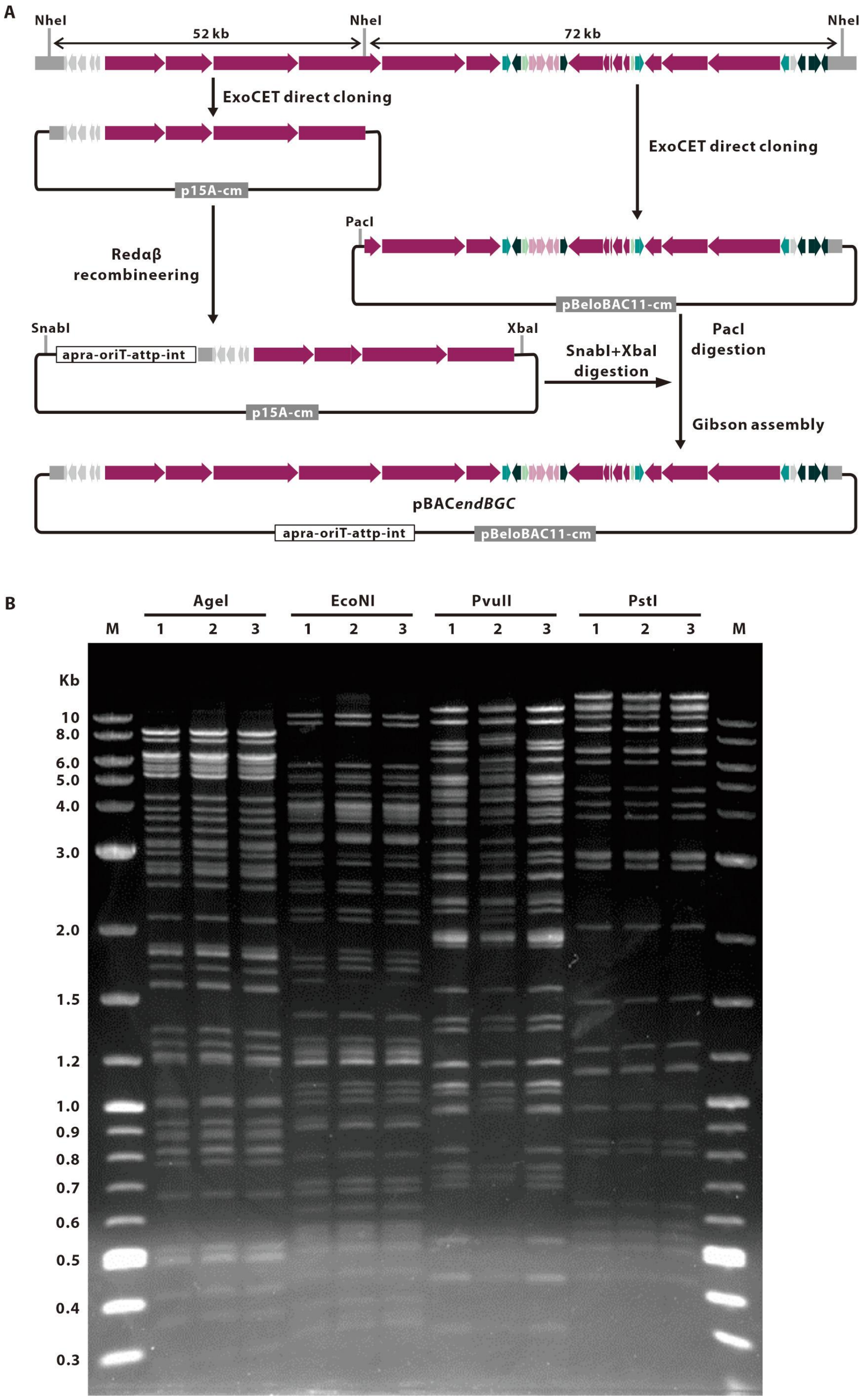

Supplementary Figure 1. Cloning of full set of *end* BGC.

(A) Construction of the *E. coli–Streptomyces* shuttle vector carrying all *end* BGC. (B) Restriction analysis of pBAC*endBGC*.

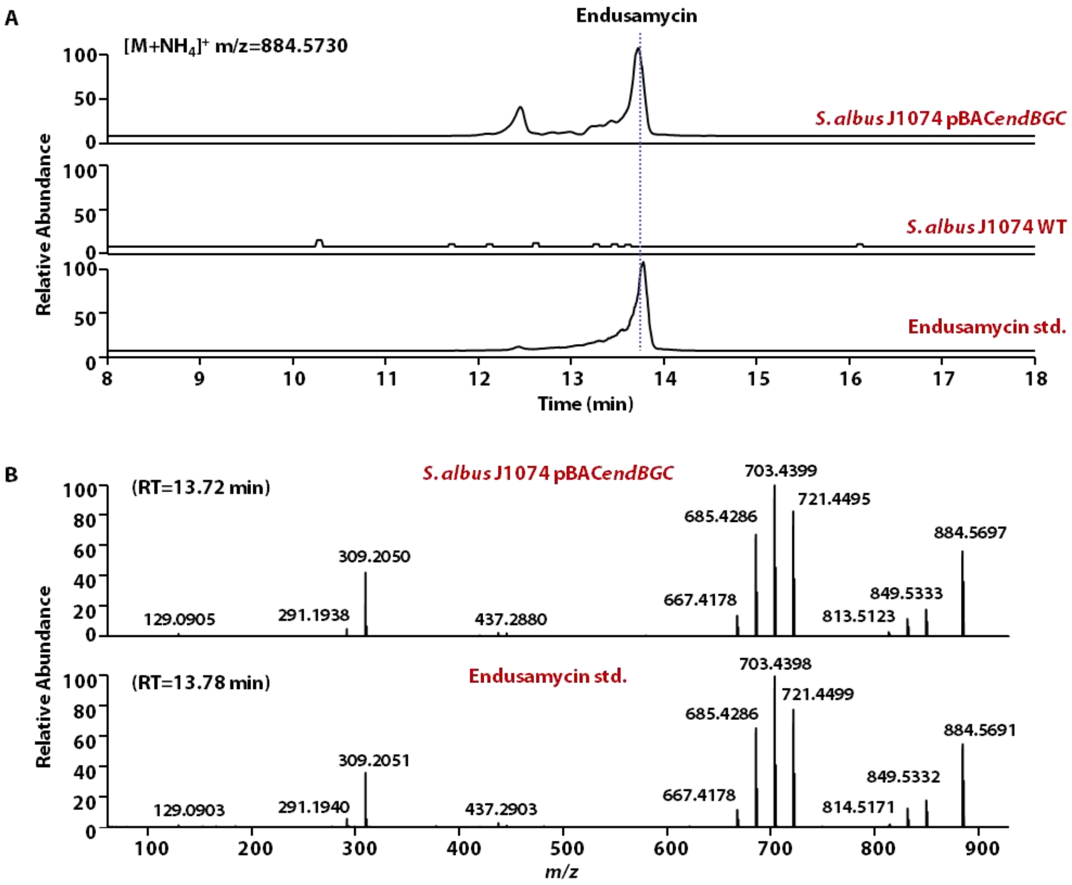

Supplementary Figure 2. Heterologous expression of the *end* BGC.

(A) HR-ESI-LCMS analysis (extracted ion chromatogram) of endusamycin production in *S. albus* J1074 pBAC*endBGC* harboring the *end* cluster. (B) Comparison of MS^2 fragmentation spectra of endusamycin produced in *S. albus* J1074 pBAC*endBGC* and the endusamycin standard.

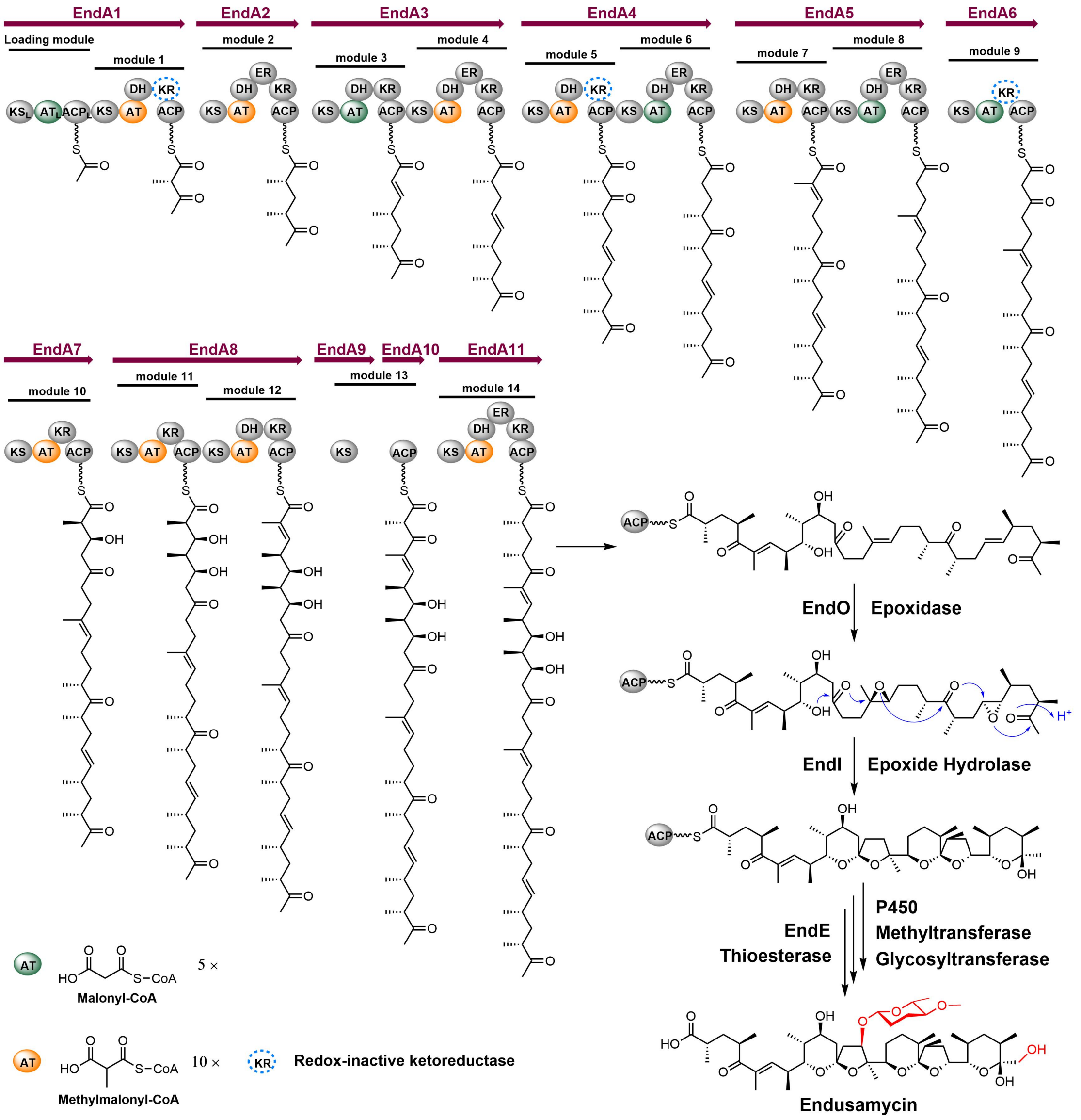

Supplementary Figure 3. Proposed biosynthetic pathway to endusamycin in *S. endus* subsp. *aureus*.

Polyketide synthase domain abbreviations found in EndA1-A11 consist of ketosynthase (KS), acyltransferase (AT), acyl carrier protein (ACP), ketoreductase (KR), dehydratase (DH), enoylreductase (ER).

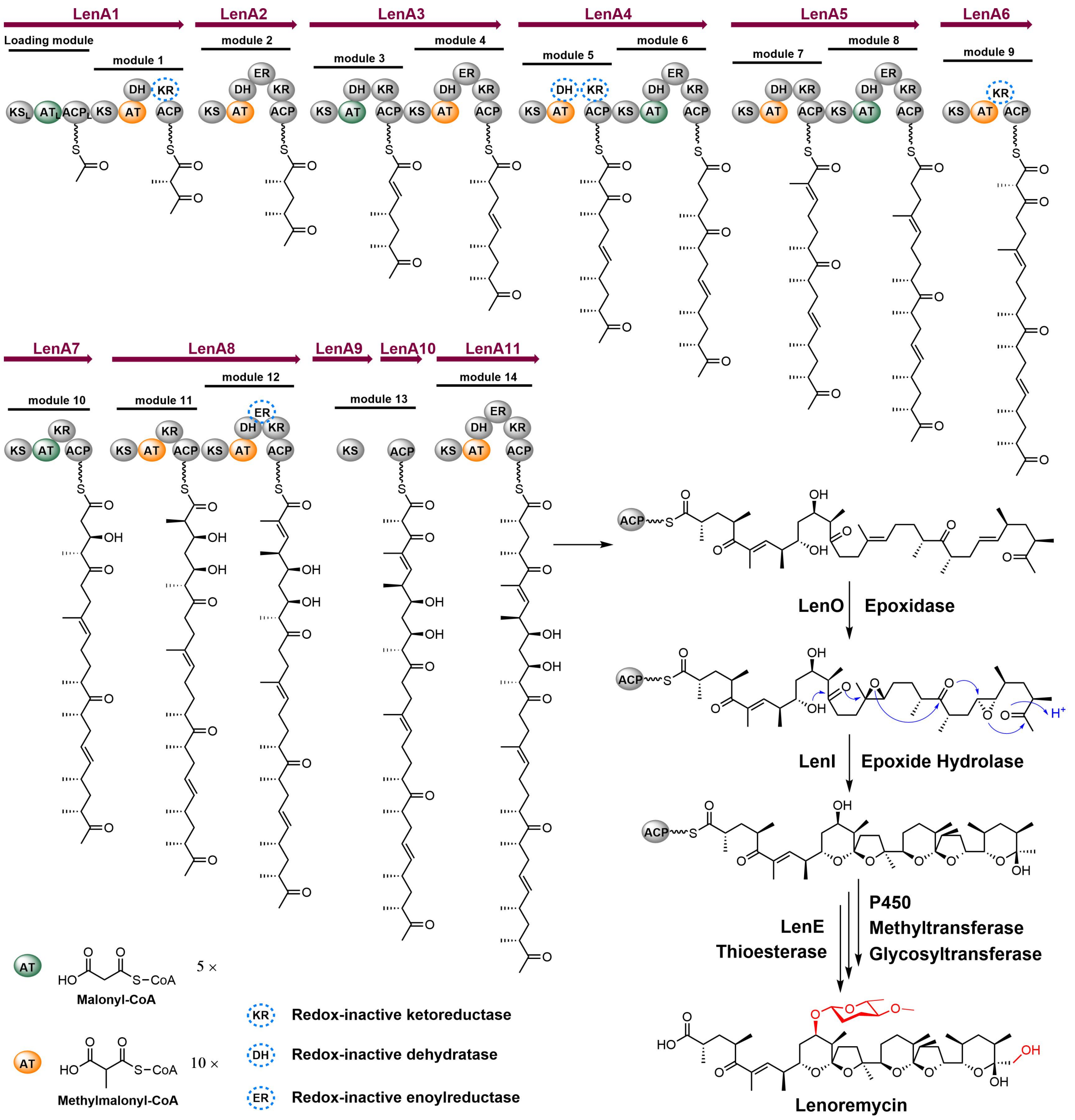

Supplementary Figure 4. Proposed biosynthetic pathway to lenoremycin in *S. hygroscopicus* A-130.

Polyketide synthase domain abbreviations found in LenA1-A11 consist of ketosynthase (KS), acyltransferase (AT), acyl carrier protein (ACP), ketoreductase (KR), dehydratase (DH), enoylreductase (ER).

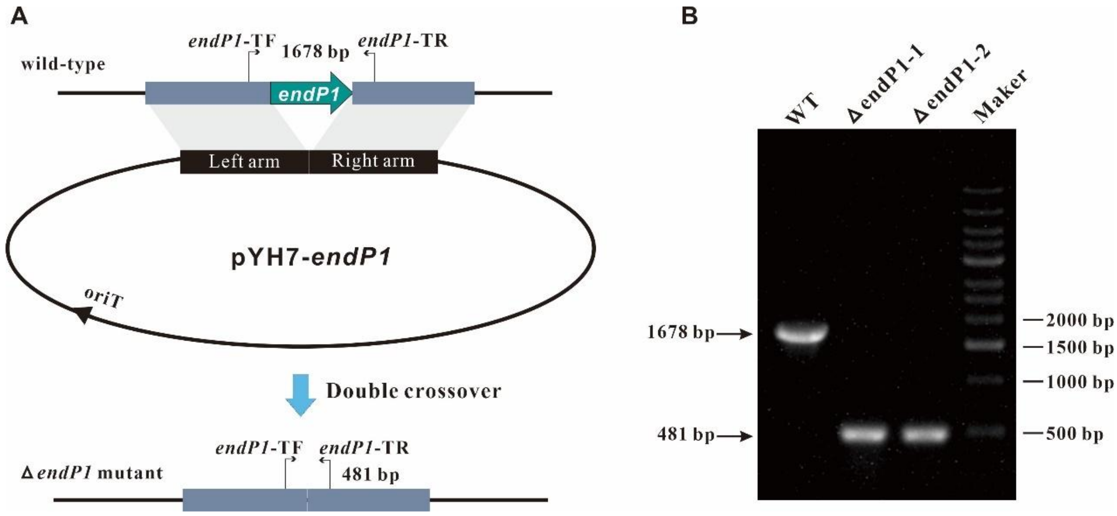

Supplementary Figure 5. Disruption of *endP1* in *S. endus* subsp. *aureus* via homologous recombination.

(A) Schematic representation for disruption of *endP1*. (B) PCR analyses of the wild-type strain and the Δ*endP1* double-cross mutant carried out using the primers listed in Table S5. WT: using the genomic DNA of *S. endus* subsp. *aureus* as template; Δ*endP1*: using the genomic DNA of Δ*endP1* mutant as template; Maker: DNA molecular ladder.

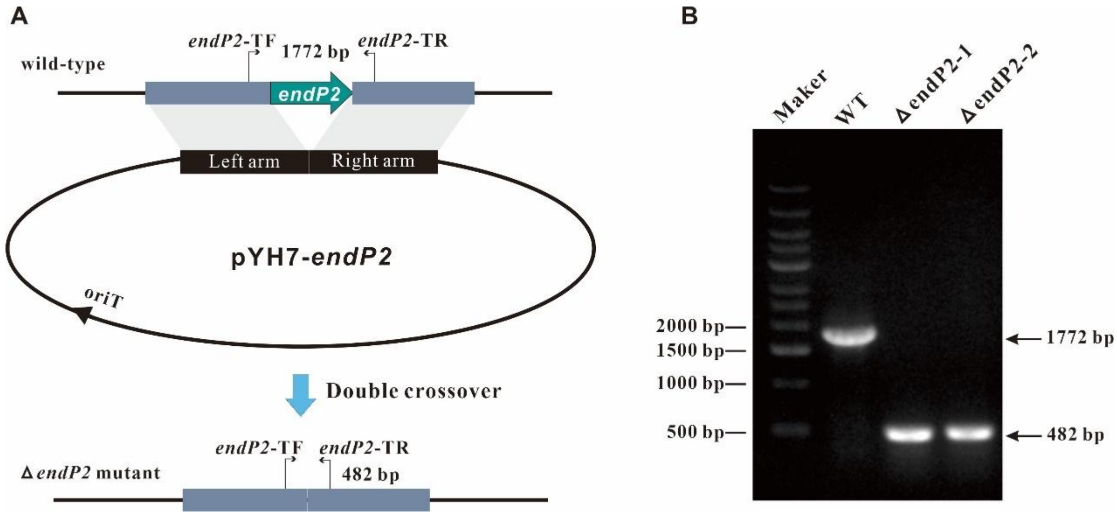

Supplementary Figure 6. Disruption of *endP2* in *S. endus* subsp. *aureus* via homologous recombination.

(A) Schematic representation for disruption of *endP2*. (B) PCR analyses of the wild-type strain and the Δ*endP2* double-cross mutant carried out using the primers listed in Table S5. WT: using the genomic DNA of *S. endus* subsp. *aureus* as template; Δ*endP2*: using the genomic DNA of Δ*endP2* mutant as template; Maker: DNA molecular ladder.

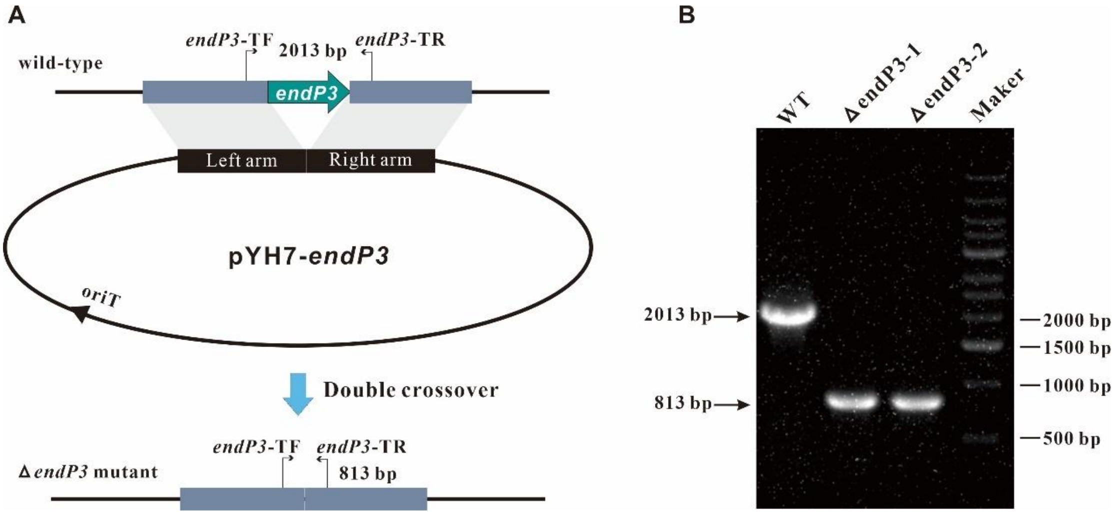

Supplementary Figure 7. Disruption of *endP3* in *S. endus* subsp. *aureus* via homologous recombination.

(A) Schematic representation for disruption of *endP3*. (B) PCR analyses of the wild-type strain and the Δ*endP3* double-cross mutant carried out using the primers listed in Table S5. WT: using the genomic DNA of *S. endus* subsp. *aureus* as template; Δ*endP3*: using the genomic DNA of Δ*endP3* mutant as template; Maker: DNA molecular ladder.

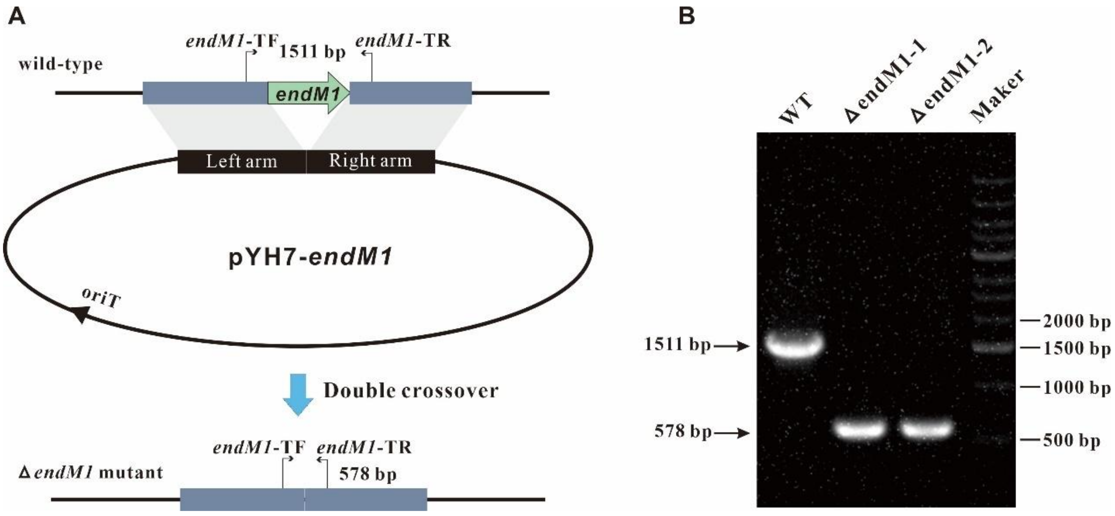

Supplementary Figure 8. Disruption of *endM1* in *S. endus* subsp. *aureus* via homologous recombination.

(A) Schematic representation for disruption of *endM1*. (B) PCR analyses of the wild-type strain and the Δ*endM1* double-cross mutant carried out using the primers listed in Table S5. WT: using the genomic DNA of *S. endus* subsp. *aureus* as template; Δ*endM1*: using the genomic DNA of Δ*endM1* mutant as template; Maker: DNA molecular ladder.

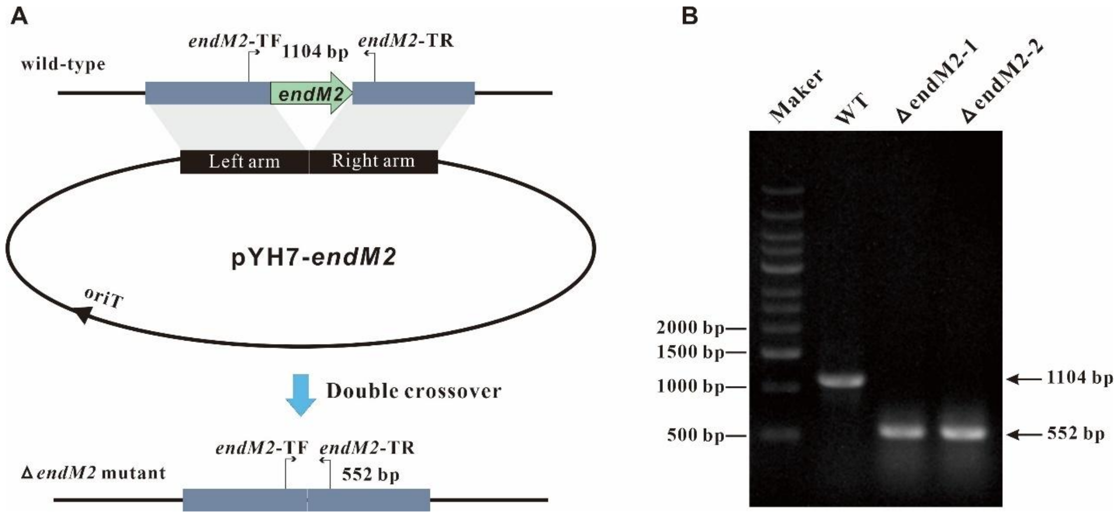

Supplementary Figure 9. Disruption of *endM2* in *S. endus* subsp. *aureus* via homologous recombination.

(A) Schematic representation for disruption of *endM2*. (B) PCR analyses of the wild-type strain and the Δ*endM2* double-cross mutant carried out using the primers listed in Table S5. WT: using the genomic DNA of *S. endus* subsp. *aureus* as template; Δ*endM2*: using the genomic DNA of Δ*endM2* mutant as template; Maker: DNA molecular ladder.

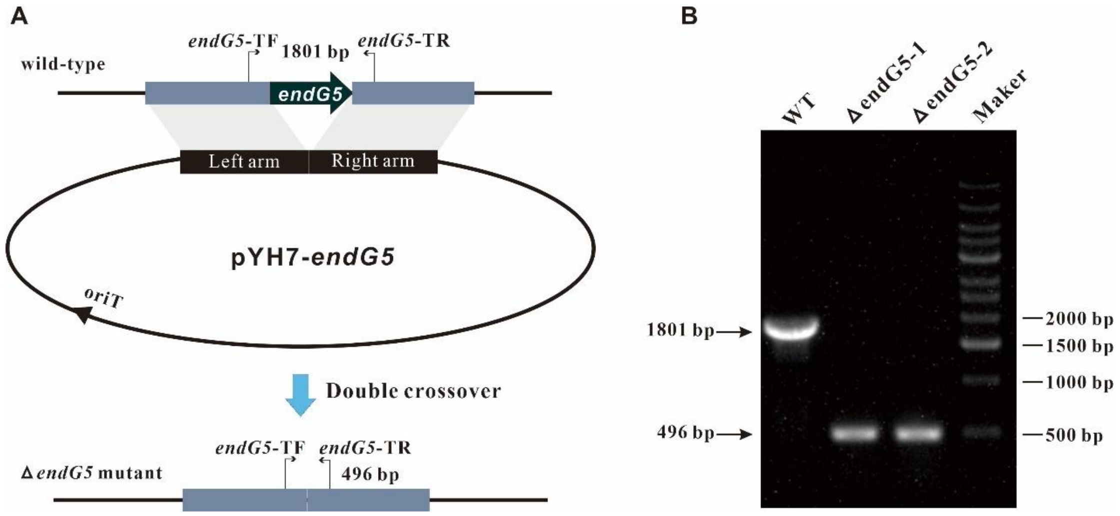

Supplementary Figure 10. Disruption of *endG5* in *S. endus* subsp. *aureus* via homologous recombination.

(A) Schematic representation for disruption of *endG5*. (B) PCR analyses of the wild-type strain and the Δ*endG5* double-cross mutant carried out using the primers listed in Table S5. WT: using the genomic DNA of *S. endus* subsp. *aureus* as template; Δ*endG5*: using the genomic DNA of Δ*endG5* mutant as template; Maker: DNA molecular ladder.

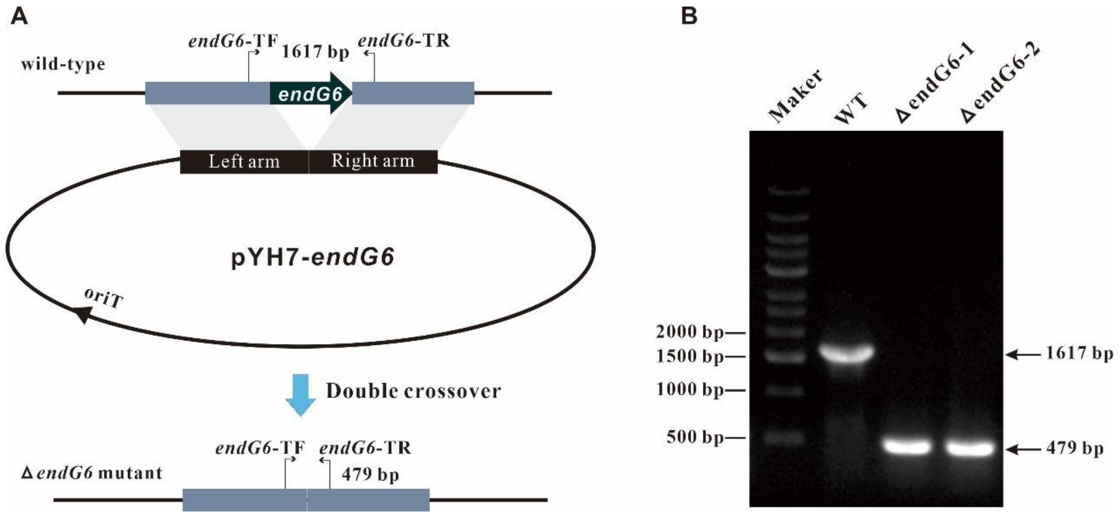

Supplementary Figure 11. Disruption of *endG6* in *S. endus* subsp. *aureus* via homologous recombination.

(A) Schematic representation for disruption of *endG6*. (B) PCR analyses of the wild-type strain and the Δ*endG6* double-cross mutant carried out using the primers listed in Table S5. WT: using the genomic DNA of *S. endus* subsp. *aureus* as template; Δ*endG6*: using the genomic DNA of Δ*endG6* mutant as template; Maker: DNA molecular ladder.

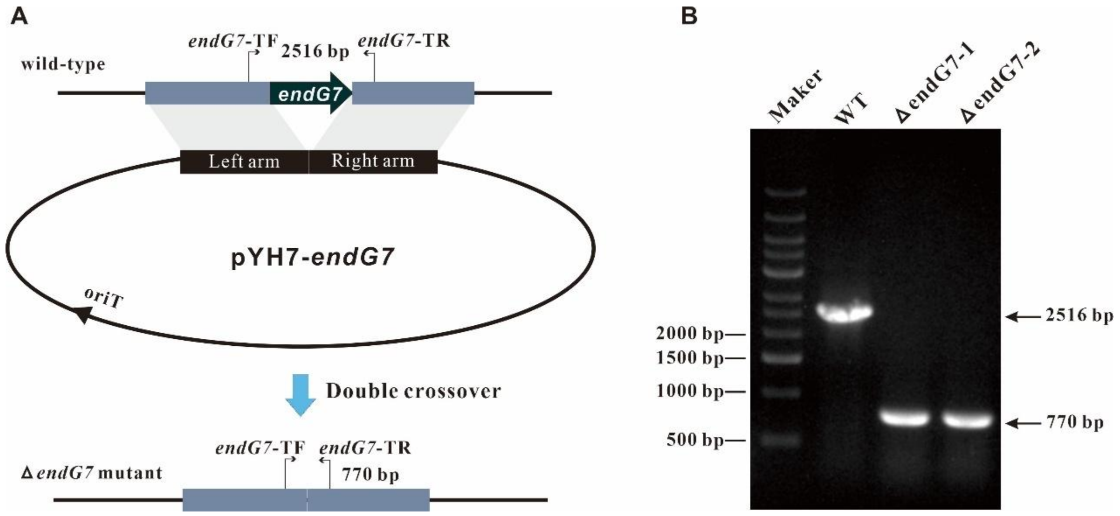

Supplementary Figure 12. Disruption of *endG7* in *S. endus* subsp. *aureus* via homologous recombination.

(A) Schematic representation for disruption of *endG7*. (B) PCR analyses of the wild-type strain and the Δ*endG7* double-cross mutant carried out using the primers listed in Table S5. WT: using the genomic DNA of *S. endus* subsp. *aureus* as template; Δ*endG7*: using the genomic DNA of Δ*endG7* mutant as template; Maker: DNA molecular ladder.

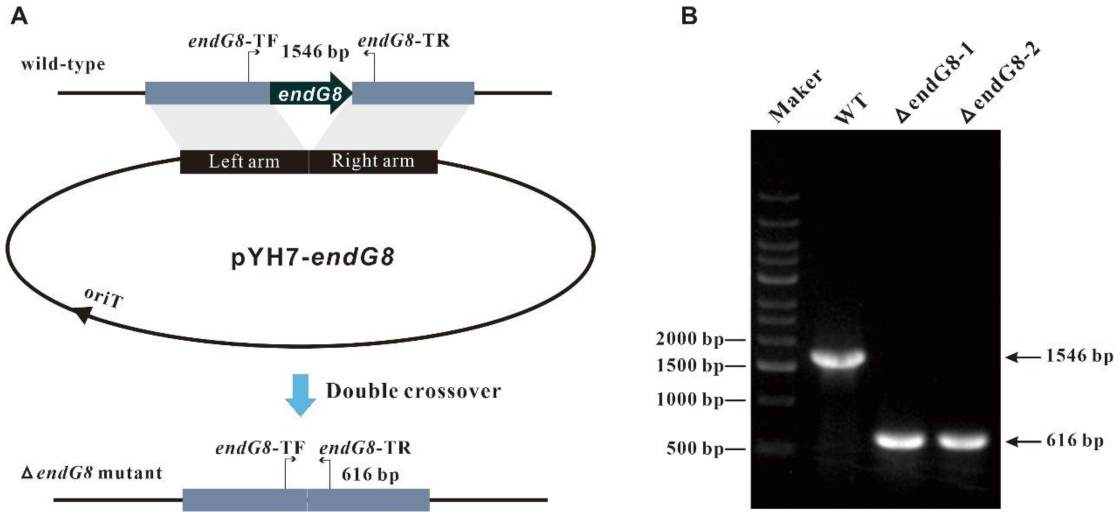

Supplementary Figure 13. Disruption of *endG8* in *S. endus* subsp. *aureus* via homologous recombination.

(A) Schematic representation for disruption of *endG8*. (B) PCR analyses of the wild-type strain and the Δ*endG8* double-cross mutant carried out using the primers listed in Table S5. WT: using the genomic DNA of *S. endus* subsp. *aureus* as template; Δ*endG8*: using the genomic DNA of Δ*endG8* mutant as template; Maker: DNA molecular ladder.

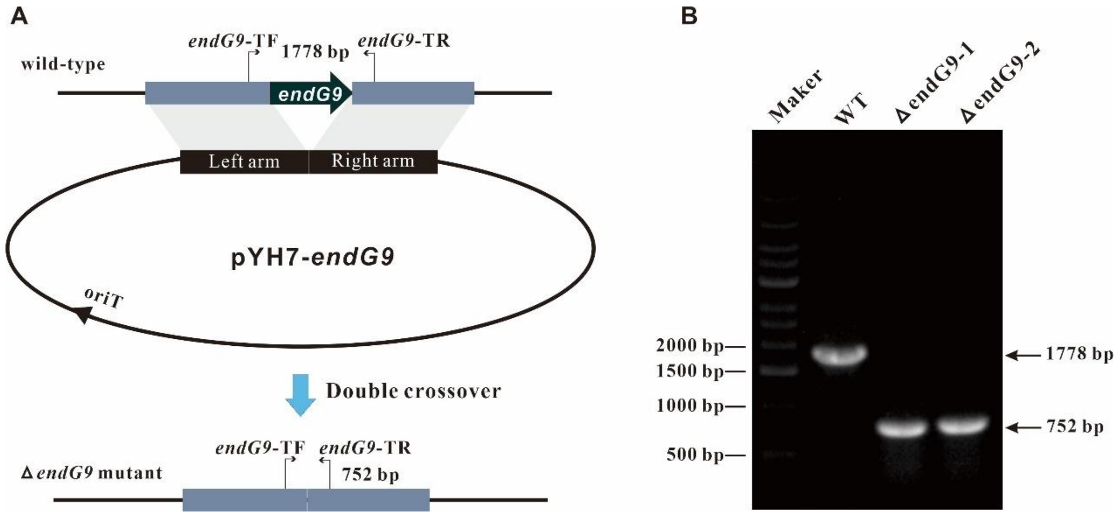

Supplementary Figure 14. Disruption of *endG9* in *S. endus* subsp. *aureus* via homologous recombination.

(A) Schematic representation for disruption of *endG9*. (B) PCR analyses of the wild-type strain and the Δ*endG9* double-cross mutant carried out using the primers listed in Table S5. WT: using the genomic DNA of *S. endus* subsp. *aureus* as template; Δ*endG9*: using the genomic DNA of Δ*endG9* mutant as template; Maker: DNA molecular ladder.

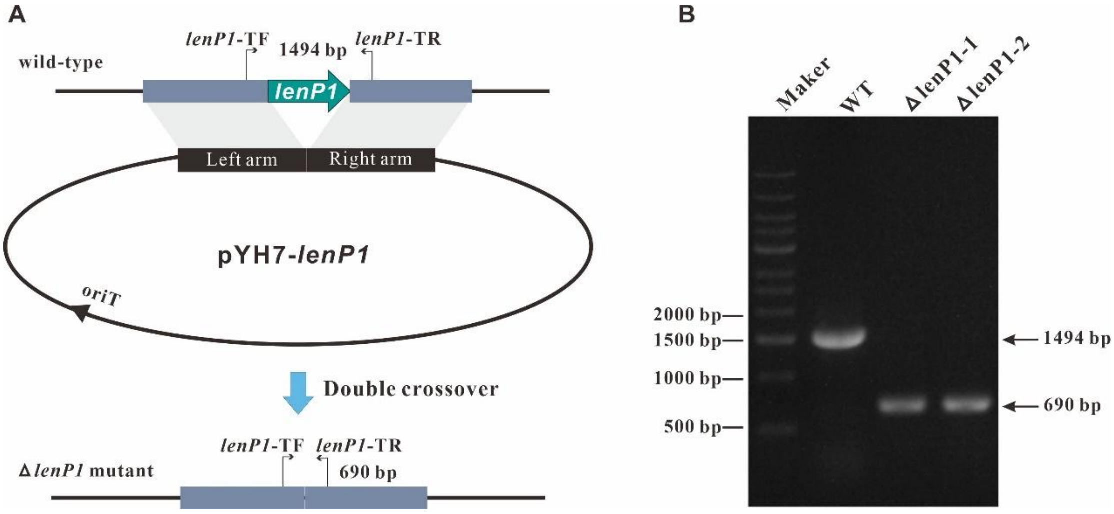

Supplementary Figure 15. Disruption of *lenP1* in *S. hygroscopicus* A-130 via homologous recombination.

(A) Schematic representation for disruption of *lenP1*. (B) PCR analyses of the wild-type strain and the Δ*lenP1* double-cross mutant carried out using the primers listed in Table S5. WT: using the genomic DNA of *S. hygroscopicus* A-130 as template; Δ*lenP1*: using the genomic DNA of Δ*lenP1* mutant as template; Maker: DNA molecular ladder.

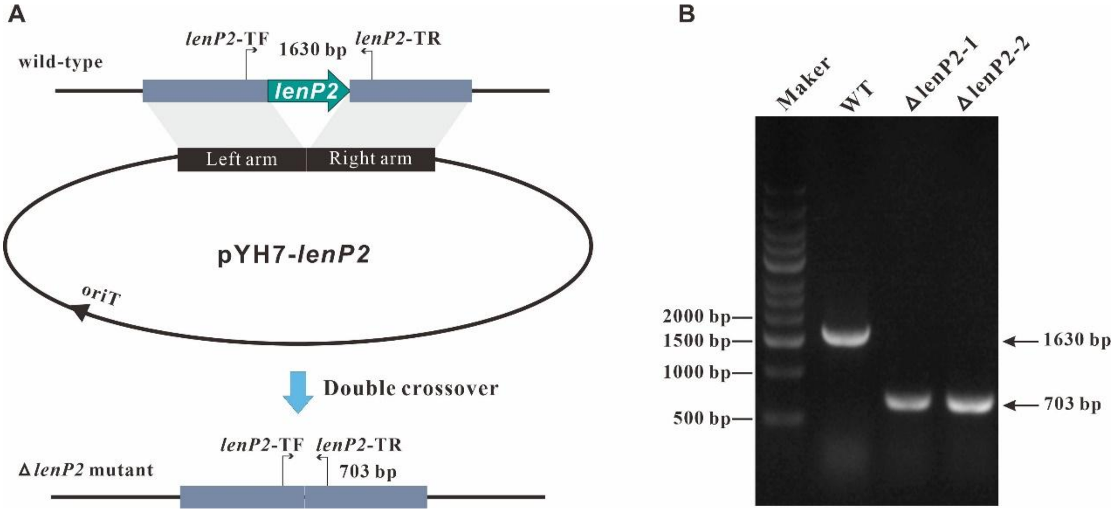

Supplementary Figure 16. Disruption of *lenP2* in *S. hygroscopicus* A-130 via homologous recombination.

(A) Schematic representation for disruption of *lenP2*. (B) PCR analyses of the wild-type strain and the Δ*lenP2* double-cross mutant carried out using the primers listed in Table S5. WT: using the genomic DNA of *S. hygroscopicus* A-130 as template; Δ*lenP2*: using the genomic DNA of Δ*lenP2* mutant as template; Maker: DNA molecular ladder.

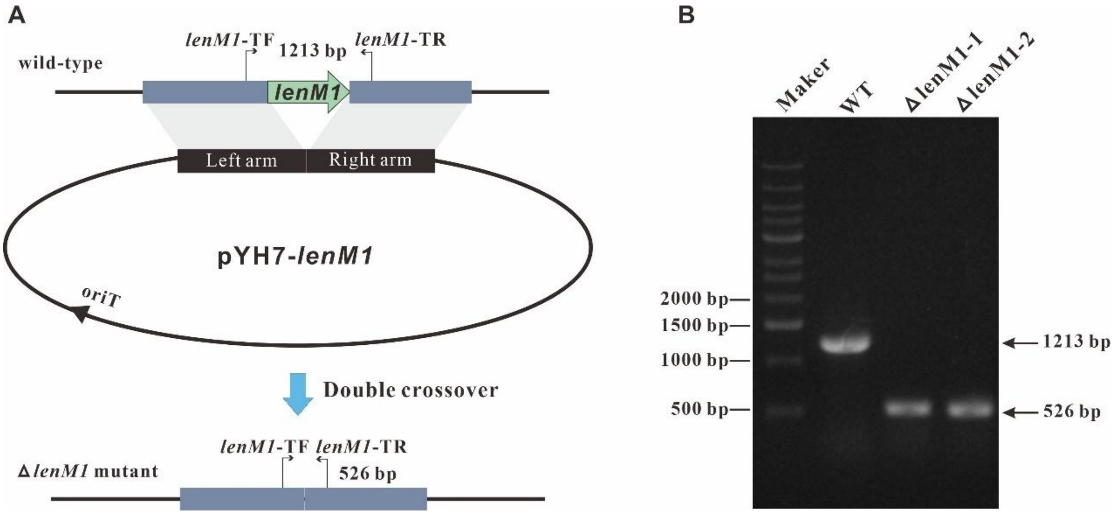

Supplementary Figure 17. Disruption of *lenM1* in *S. hygroscopicus* A-130 via homologous recombination.

(A) Schematic representation for disruption of *lenM1*. (B) PCR analyses of the wild-type strain and the Δ*lenM1* double-cross mutant carried out using the primers listed in Table S5. WT: using the genomic DNA of *S. hygroscopicus* A-130 as template; Δ*lenM1*: using the genomic DNA of Δ*lenM1* mutant as template; Maker: DNA molecular ladder.

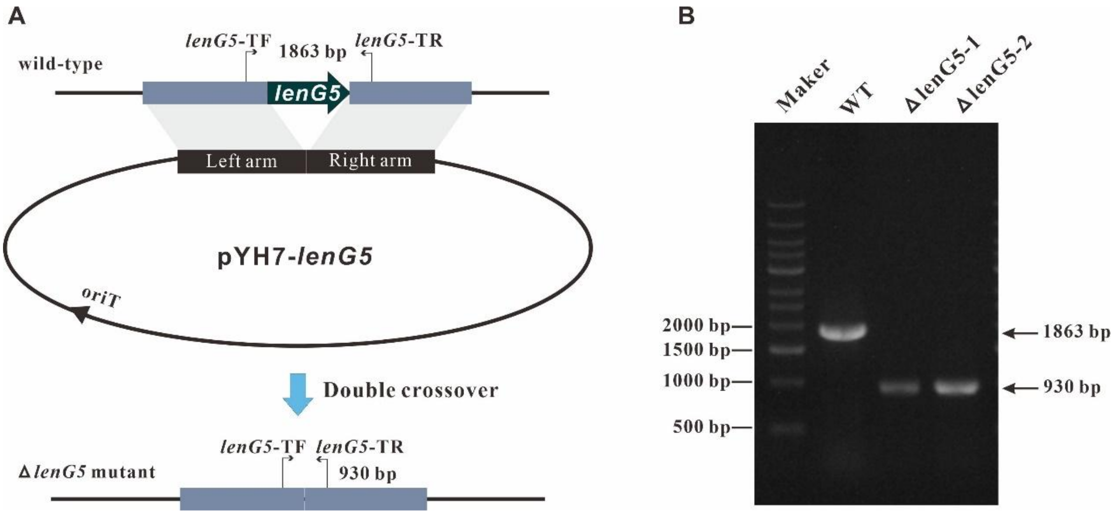

Supplementary Figure 18. Disruption of *lenG5* in *S. hygroscopicus* A-130 via homologous recombination.

(A) Schematic representation for disruption of *lenG5*. (B) PCR analyses of the wild-type strain and the Δ*lenG5* double-cross mutant carried out using the primers listed in Table S5. WT: using the genomic DNA of *S. hygroscopicus* A-130 as template; Δ*lenG5*: using the genomic DNA of Δ*lenG5* mutant as template; Maker: DNA molecular ladder.

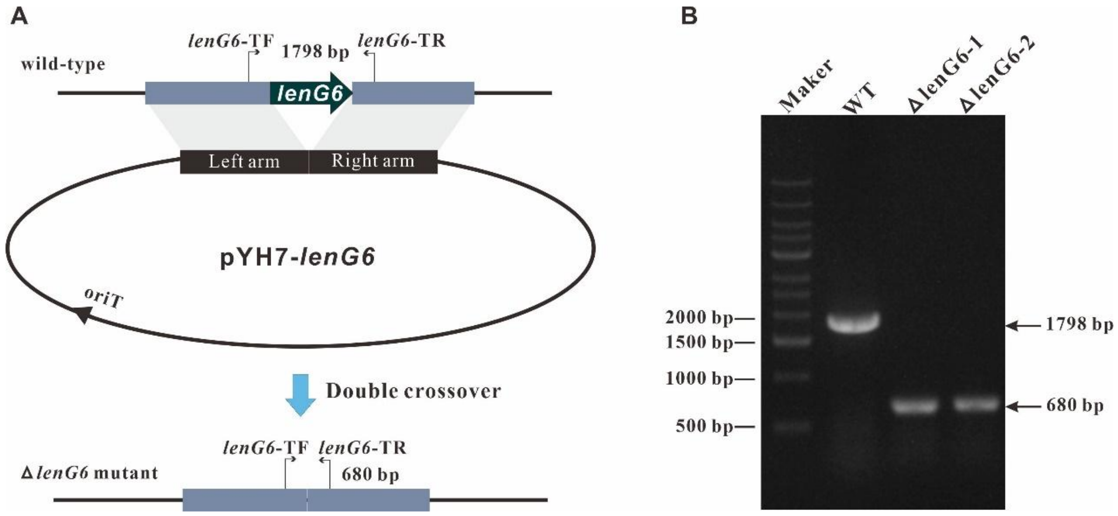

Supplementary Figure 19. Disruption of *lenG6* in *S. hygroscopicus* A-130 via homologous recombination.

(A) Schematic representation for disruption of *lenG6*. (B) PCR analyses of the wild-type strain and the Δ*lenG6* double-cross mutant carried out using the primers listed in Table S5. WT: using the genomic DNA of *S. hygroscopicus* A-130 as template; Δ*lenG6*: using the genomic DNA of Δ*lenG6* mutant as template; Maker: DNA molecular ladder.

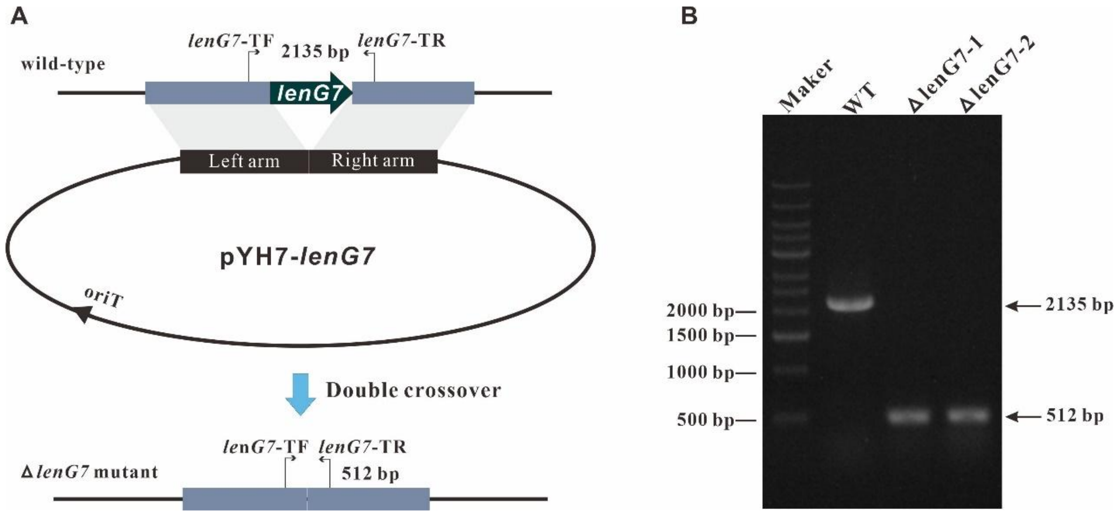

Supplementary Figure 20. Disruption of *lenG7* in *S. hygroscopicus* A-130 via homologous recombination.

(A) Schematic representation for disruption of *lenG7*. (B) PCR analyses of the wild-type strain and the Δ*lenG7* double-cross mutant carried out using the primers listed in Table S5. WT: using the genomic DNA of *S. hygroscopicus* A-130 as template; Δ*lenG7*: using the genomic DNA of Δ*lenG7* mutant as template; Maker: DNA molecular ladder.

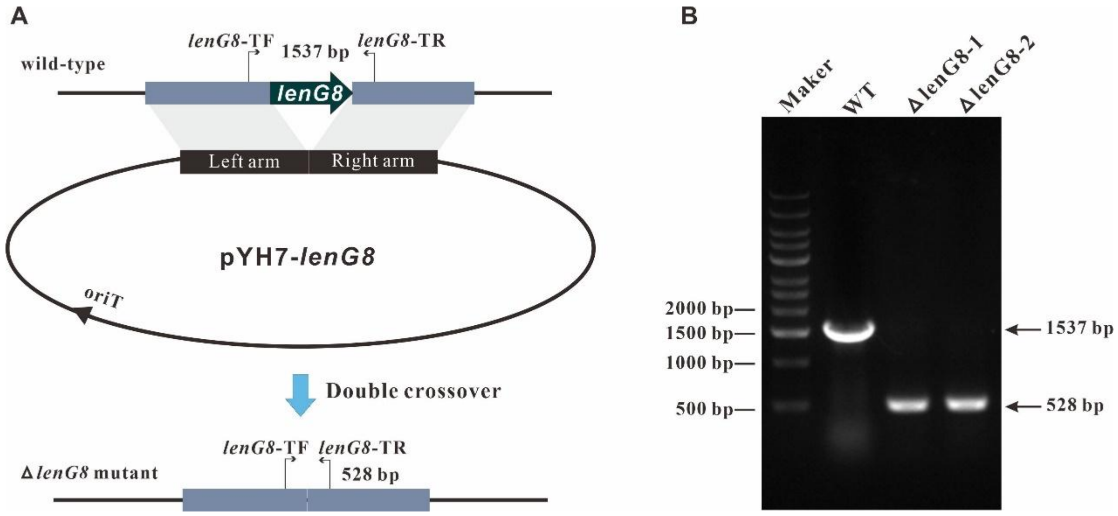

Supplementary Figure 21. Disruption of *lenG8* in *S. hygroscopicus* A-130 via homologous recombination.

(A) Schematic representation for disruption of *lenG8*. (B) PCR analyses of the wild-type strain and the Δ*lenG8* double-cross mutant carried out using the primers listed in Table S5. WT: using the genomic DNA of *S. hygroscopicus* A-130 as template; Δ*lenG8*: using the genomic DNA of Δ*lenG8* mutant as template; Maker: DNA molecular ladder.

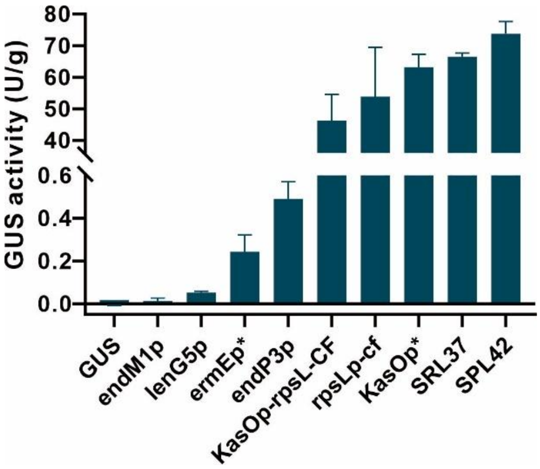

Supplementary Figure 22. Strength evaluation of selected promoters in *S. endus* subsp. *aureus.*

The strength of each promoter was assessed based on β-glucuronidase (GUS) activity. Data is presented as mean values ± SD (n = 3).

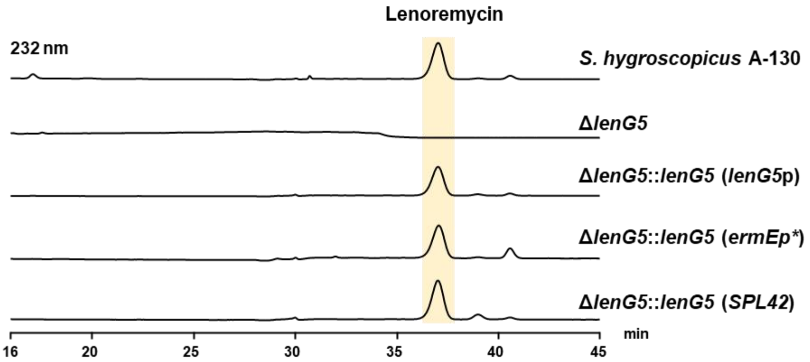

Supplementary Figure 23. HPLC profiles of extracts from the *S. hygroscopicus* A-130 wild-type, Δ*lenG5* mutant and gene complementation strains.

Complementation of the Δ*lenG5* mutant with pYL1 (pSET152 derivative for heterologous expression of *lenG5* under the control of *lenG5p* promoter), pYL2 (pSET152 derivative for heterologous expression of *lenG5* under the control of *ermEp** promoter) and pYL3 (pSET152 derivative for heterologous expression of *lenG5* under the control of *SPL42* promoter), respectively, led to restoration of Lenoremycin production. This result confirms the validity of pYL1, pYL2 and pYL3.

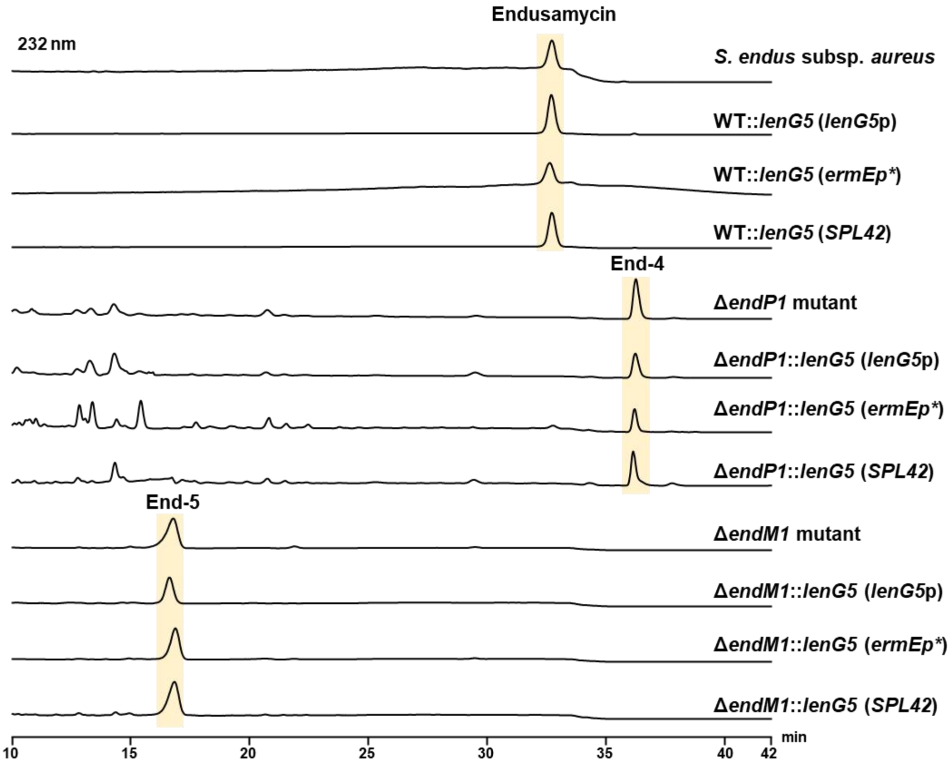

Supplementary Figure 24. HPLC profiles of extracts from *S. endus* subsp. *aureus* wild-type (WT), Δ*endP1*, Δ*endM1* and hybrid strains.

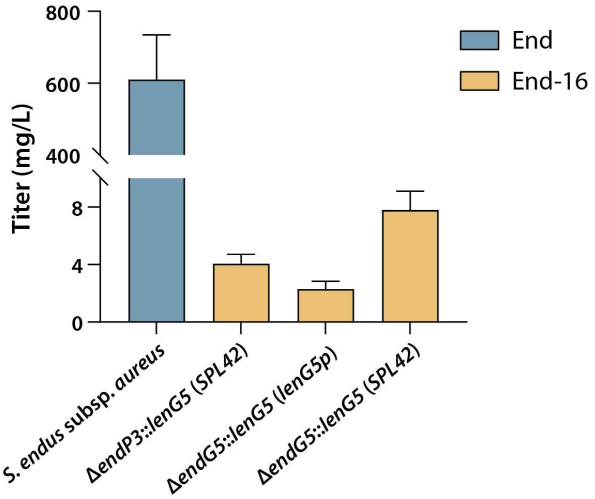

Supplementary Figure 25. Comparison of the titers of End produced by wild-type strain of *S. endus* subsp. *aureus* and End-16 produced by Δ*endP3*::*lenG5* (*SPL42*), Δ*endG5*::*lenG5* (*lenG5p*), and Δ*endG5*::*lenG5* (*SPL42*) mutants.

Data is presented as mean values ± SD (n = 3).

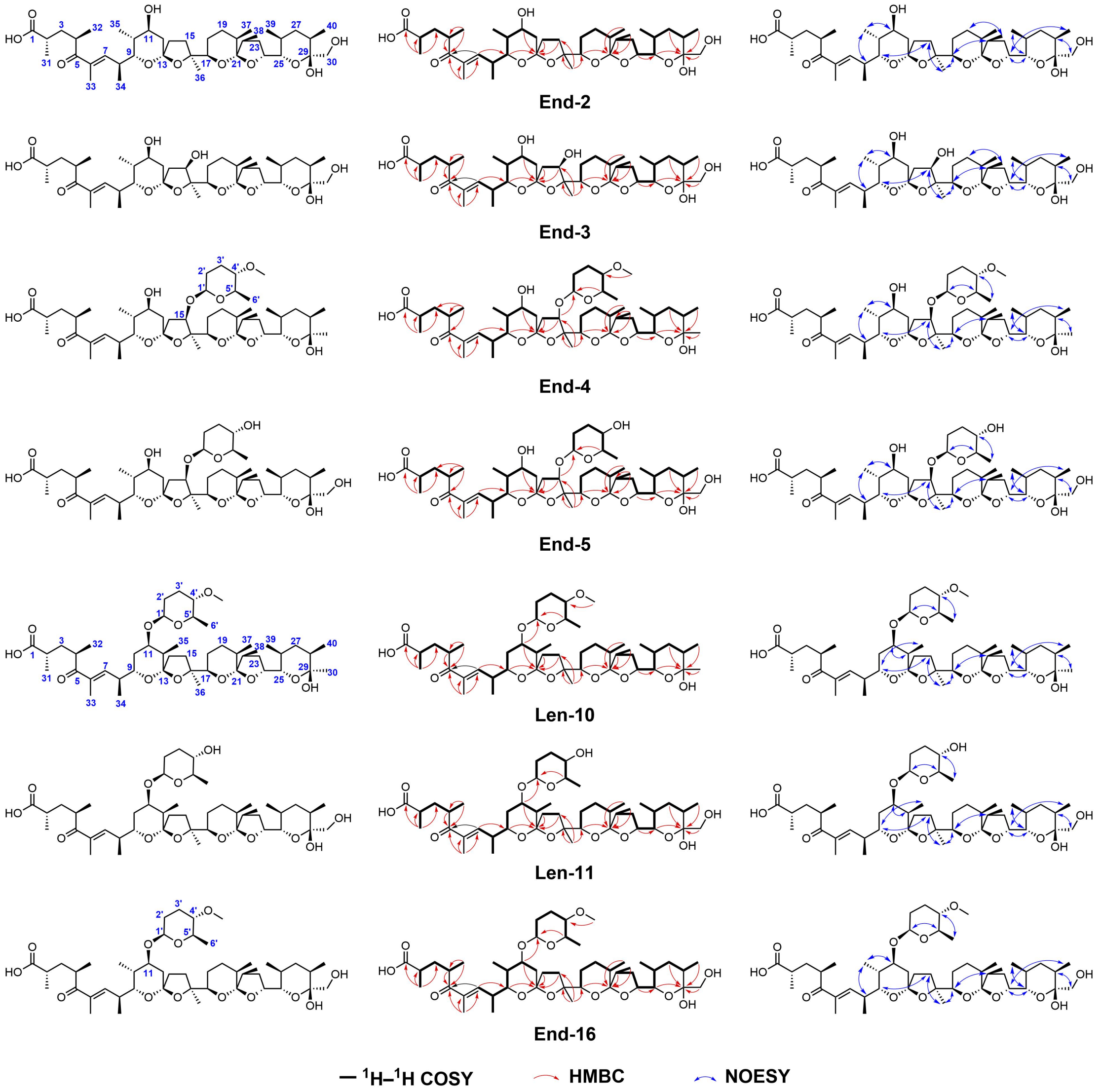

Supplementary Figure 26. Key ^{1}H–^{1}H COSY, HMBC, and NOESY correlations of End-2, End-3, End-4, End-5, Len-10. Len-11, End-16.

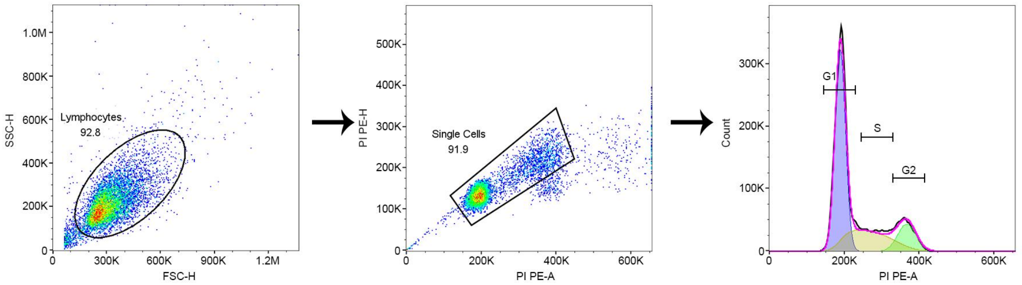

Supplementary Figure 27. Gating strategy for flow cytometry.

The cell population was first gated for whole cells and cell debris (FSC and SSC), then for single cells (PI PE-A and PI PE-H), and lastly for cells in G1, S, or G2 phase according to DNA content. This gated strategy was used in all flow cytometric cell cycle correlation analyses in this study.

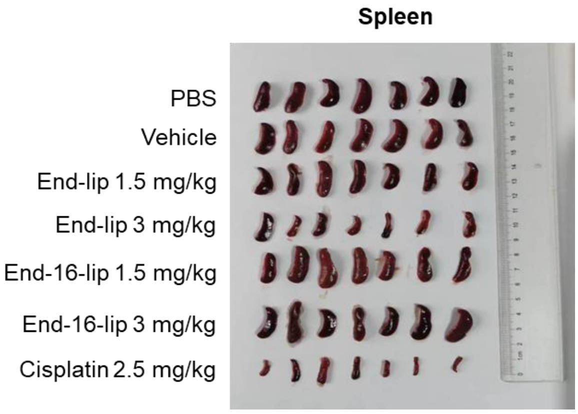

Supplementary Figure 28. Image of spleen tissues from mice in each group.

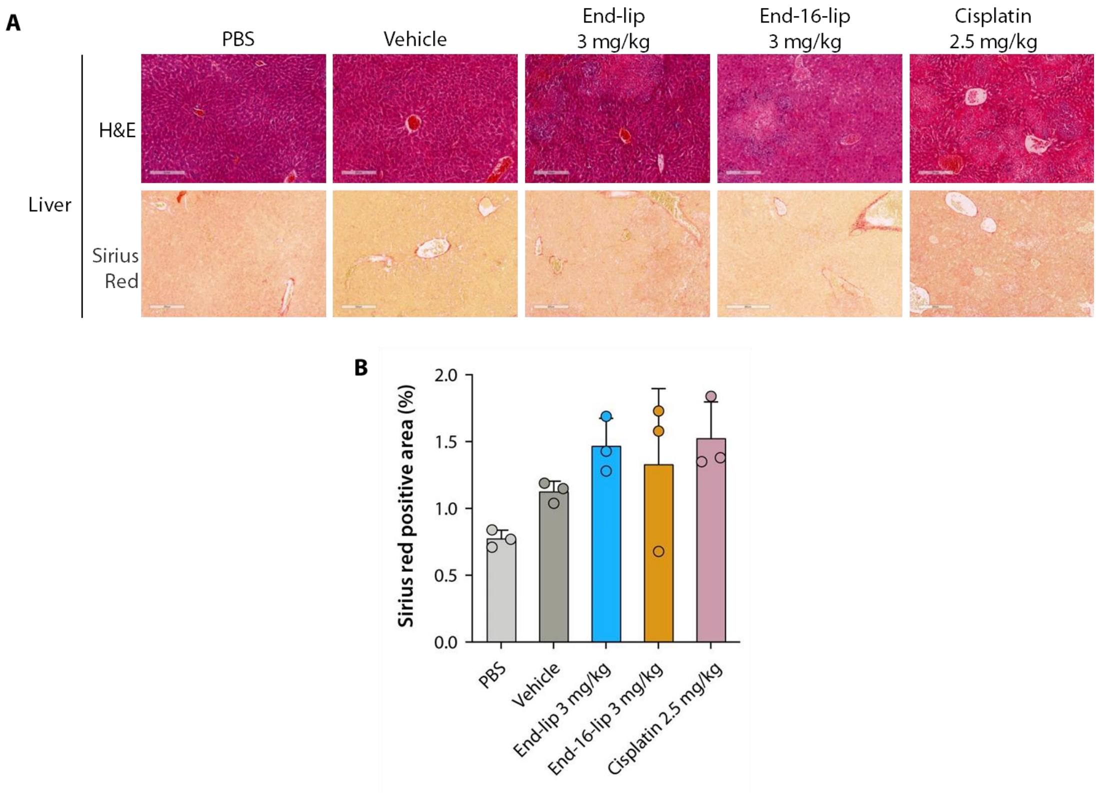

Supplementary Figure 29. H&E staining and Sirius Red staining of liver tissues.

(A) Representative images of haematoxylin and eosin (H&E) and Sirius red stained liver sections. (B) Statistical analyses of Sirius Red-stained liver sections. Data presented as means ± SDs (n = 3).

NMR spectrum, HRMS spectrum, Optical rotation value, UV spectrum, IR spectrum and ECD spectra

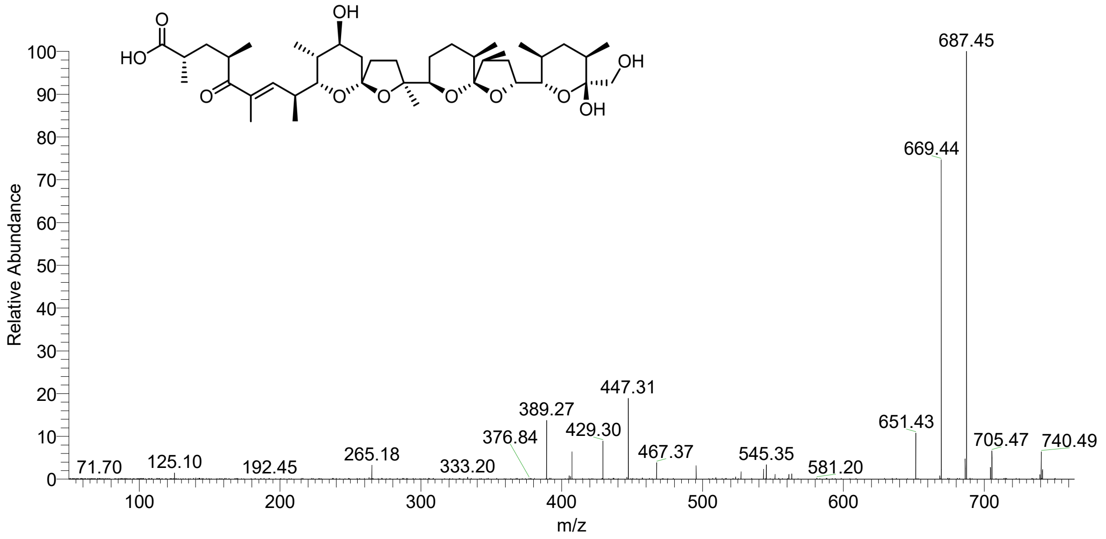

MS^2 fragmentation spectra of End-2.

Rudolph Research Analytical

This sample was measured on an Autopol VI, Serial #91058
Manufactured by Rudolph Research Analytical, Hackettstown, NJ, USA.

Measurement Date : Thursday, 25-AUG-2022

Set Temperature : OFF

Time Delay : Disabled

Delay between Measurement : Disabled

n	Average	Std.Dev.	% RSD	Maximum	Minimum
5	49.08	0.84	1.71	50.30	48.30

S.No	Sample ID	Time	Result	Scale	OR °Arc	WLG.nm	Lg.mm	Conc.g/100ml	Temp.
1	740	12:25:36 PM	50.30	SR	0.0503	589	100.00	0.100	26.6
2	740	12:25:44 PM	49.60	SR	0.0496	589	100.00	0.100	26.6
3	740	12:25:52 PM	48.70	SR	0.0487	589	100.00	0.100	26.6
4	740	12:26:01 PM	48.30	SR	0.0483	589	100.00	0.100	26.5
5	740	12:26:09 PM	48.50	SR	0.0485	589	100.00	0.100	26.5

Optical rotation value of End-2.

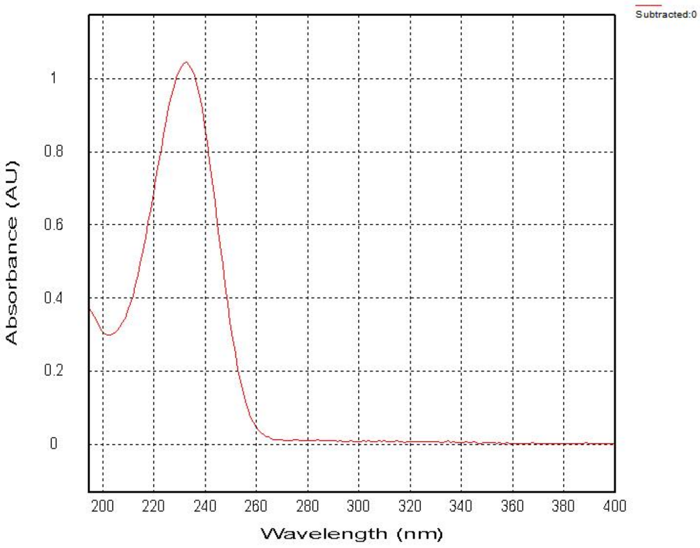

UV spectrum of End-2.

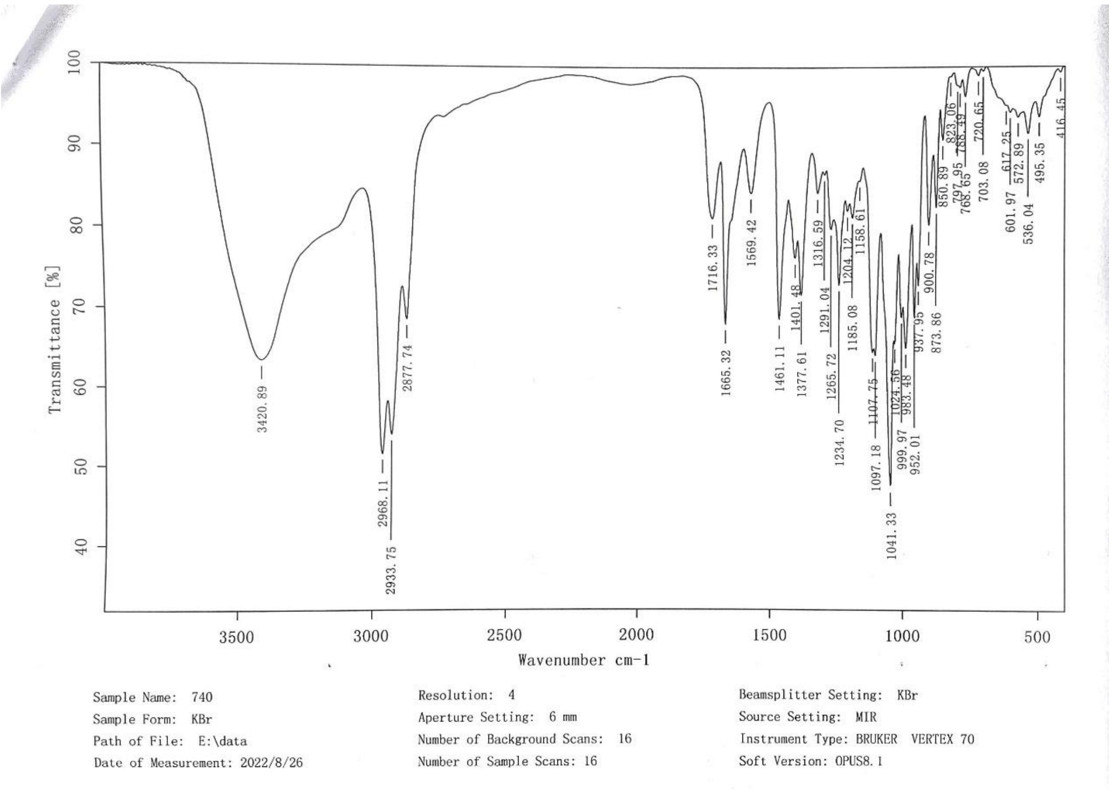

IR spectrum of End-2.

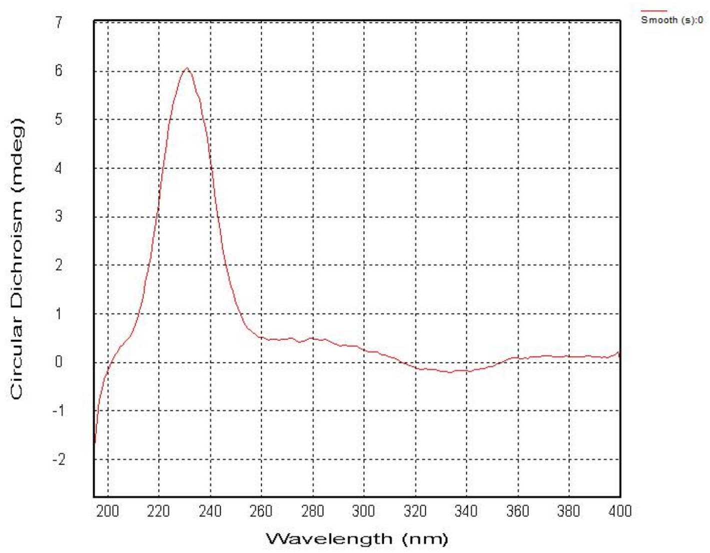

Experimental ECD spectra of End-2.

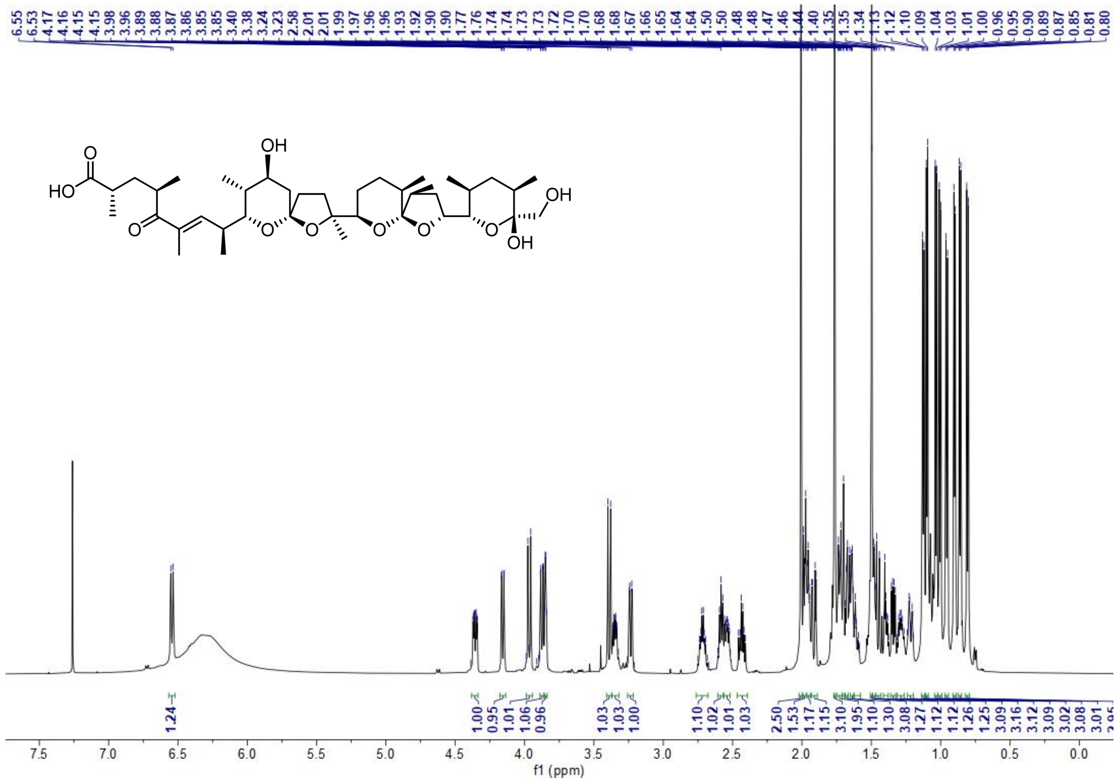

^{1}H NMR spectrum of End-2 in $CDCl_3$.

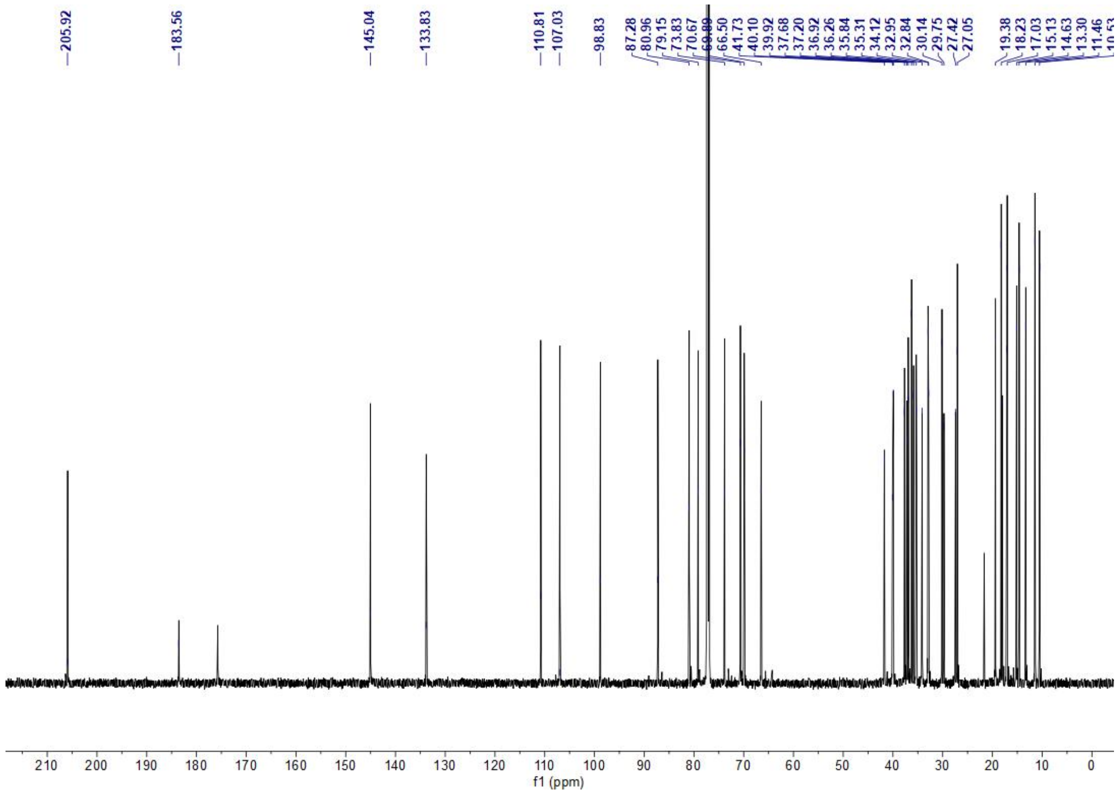

^{13}C NMR spectrum of End-2 in $CDCl_3$.

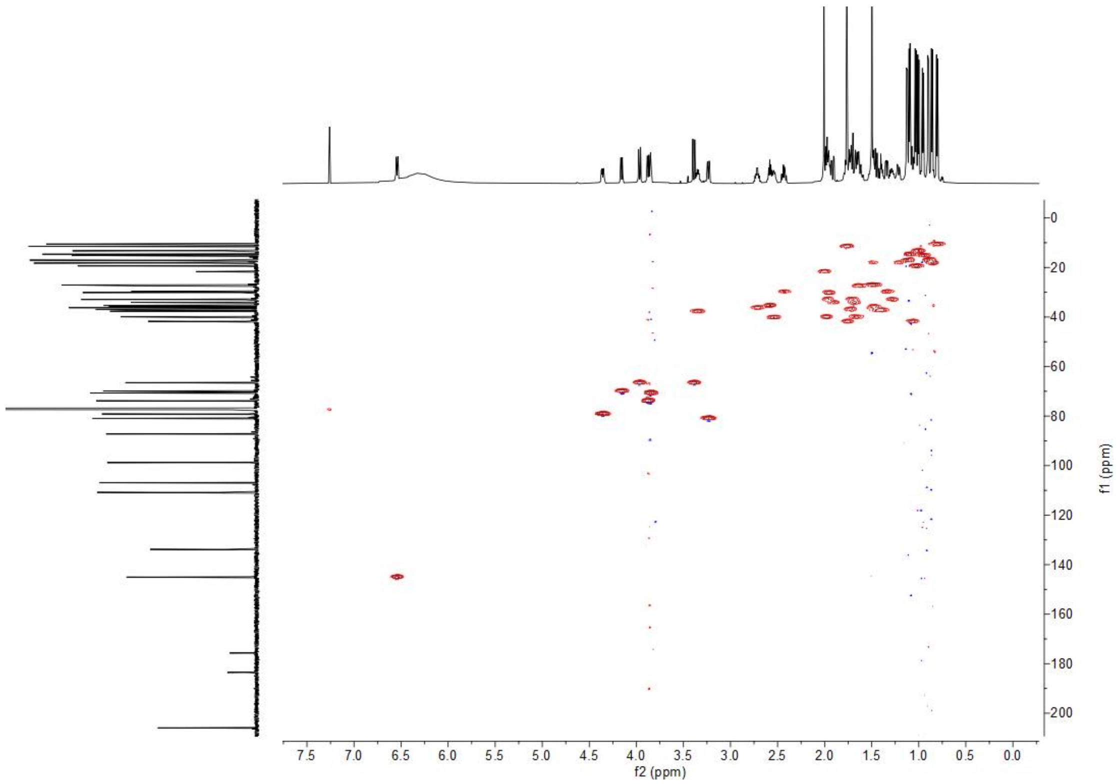

HSQC spectrum of End-2 in $CDCl_3$.

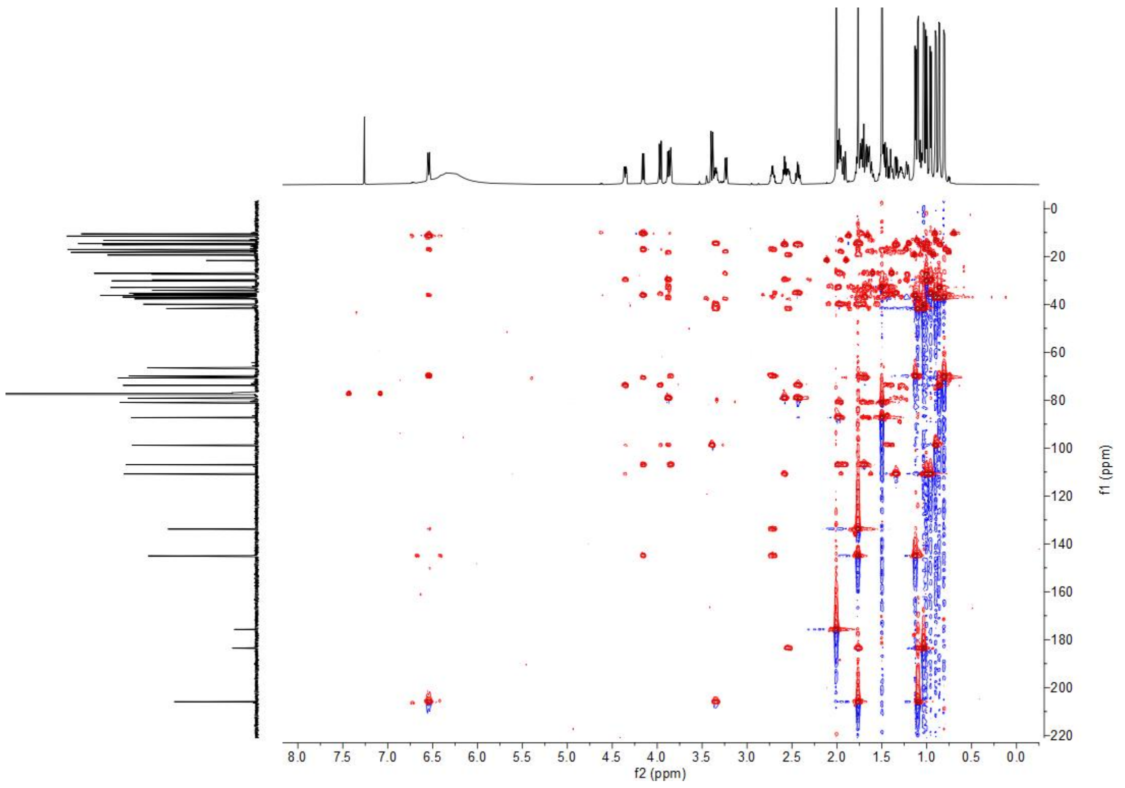

HMBC spectrum of End-2 in CDCl$_3$.

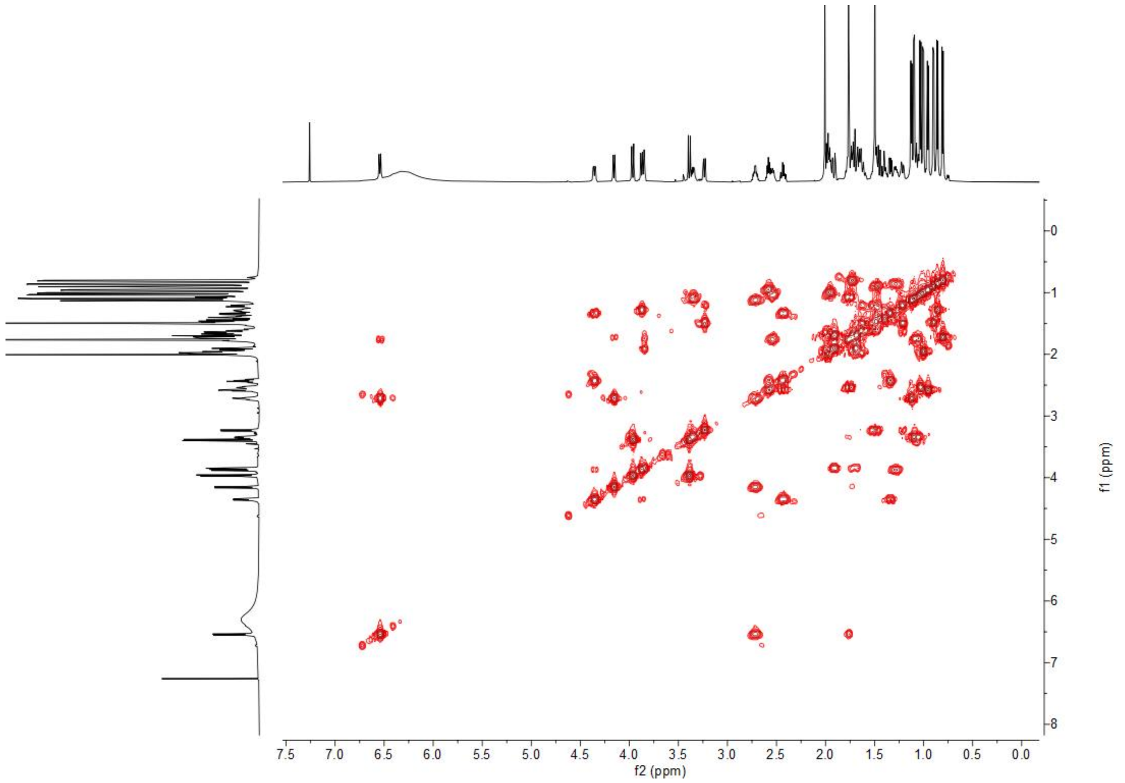

$^1H-^1H$ COSY spectrum of End-2 in CDCl$_3$.

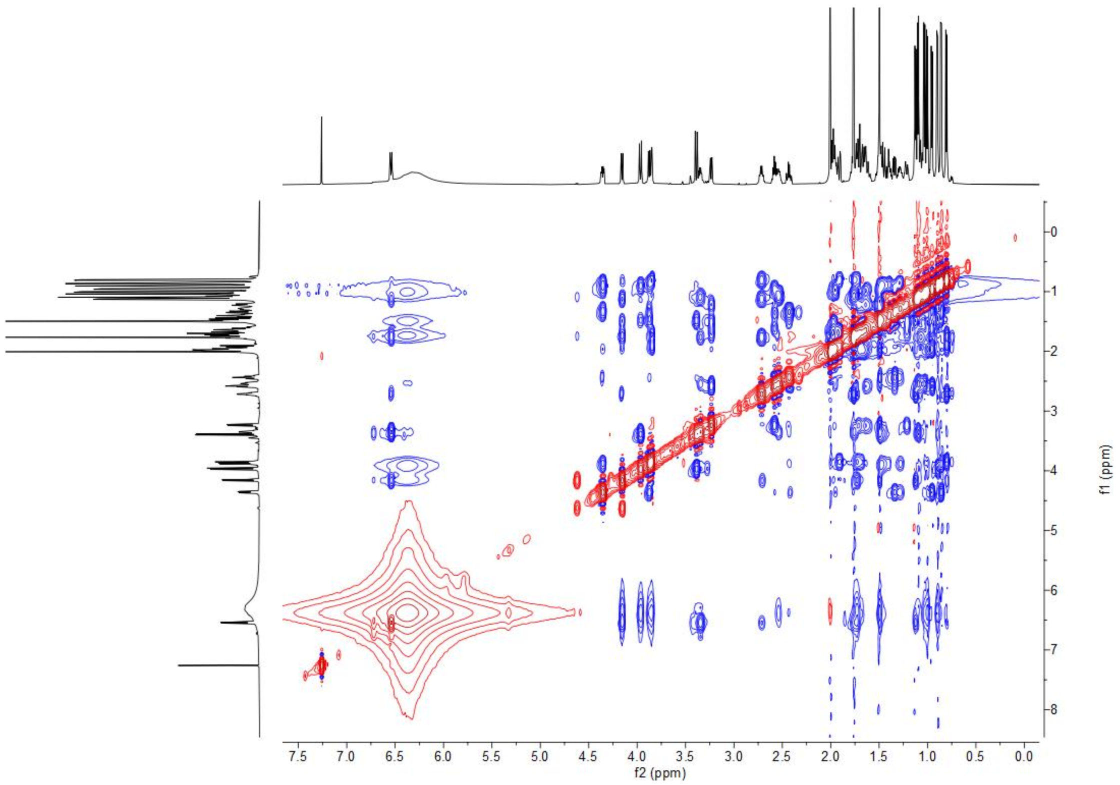

NOESY spectrum of End-2 in $CDCl_3$.

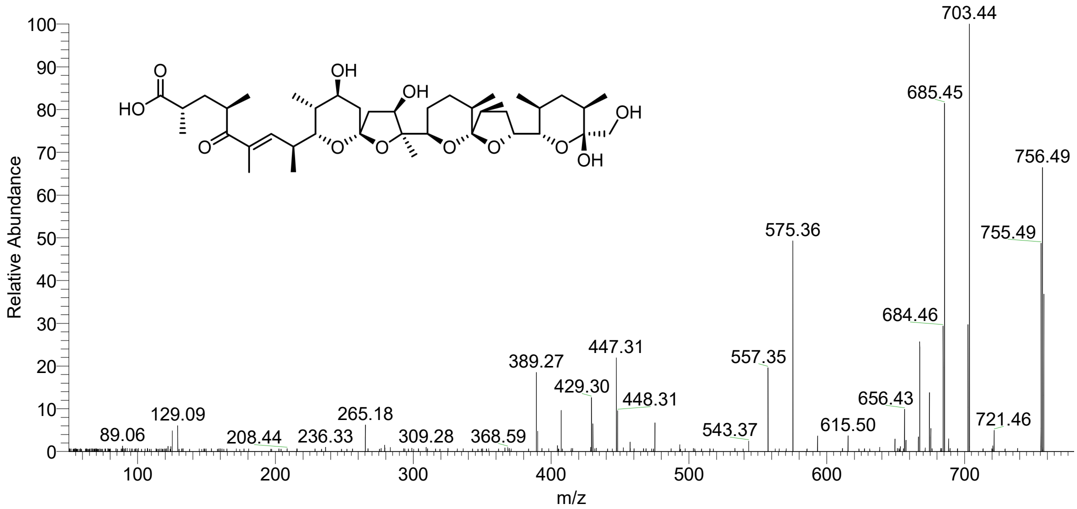

MS^2 fragmentation spectra of End-3.

Rudolph Research Analytical

This sample was measured on an Autopol VI, Serial #91058
Manufactured by Rudolph Research Analytical, Hackettstown, NJ, USA.

Measurement Date : Tuesday, 20-SEP-2022

Set Temperature : OFF

Time Delay : Disabled

Delay between Measurement : Disabled

n	Average	Std.Dev.	% RSD	Maximum	Minimum
5	44.72	0.35	0.78	45.20	44.30

S.No	Sample ID	Time	Result	Scale	OR °Arc	WLG.nm	Lg.mm	Conc.g/100ml	Temp.
1	YP-4-756	02:39:03 PM	45.20	SR	0.0452	589	100.00	0.100	25.3
2	YP-4-756	02:39:12 PM	44.90	SR	0.0449	589	100.00	0.100	25.2
3	YP-4-756	02:39:20 PM	44.50	SR	0.0445	589	100.00	0.100	25.2
4	YP-4-756	02:39:28 PM	44.70	SR	0.0447	589	100.00	0.100	25.2
5	YP-4-756	02:39:36 PM	44.30	SR	0.0443	589	100.00	0.100	25.2

Optical rotation value of End-3.

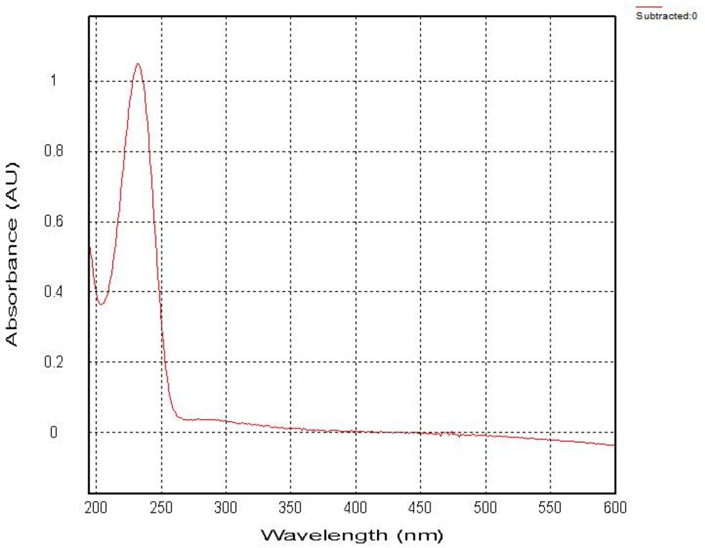

UV spectrum of End-3.

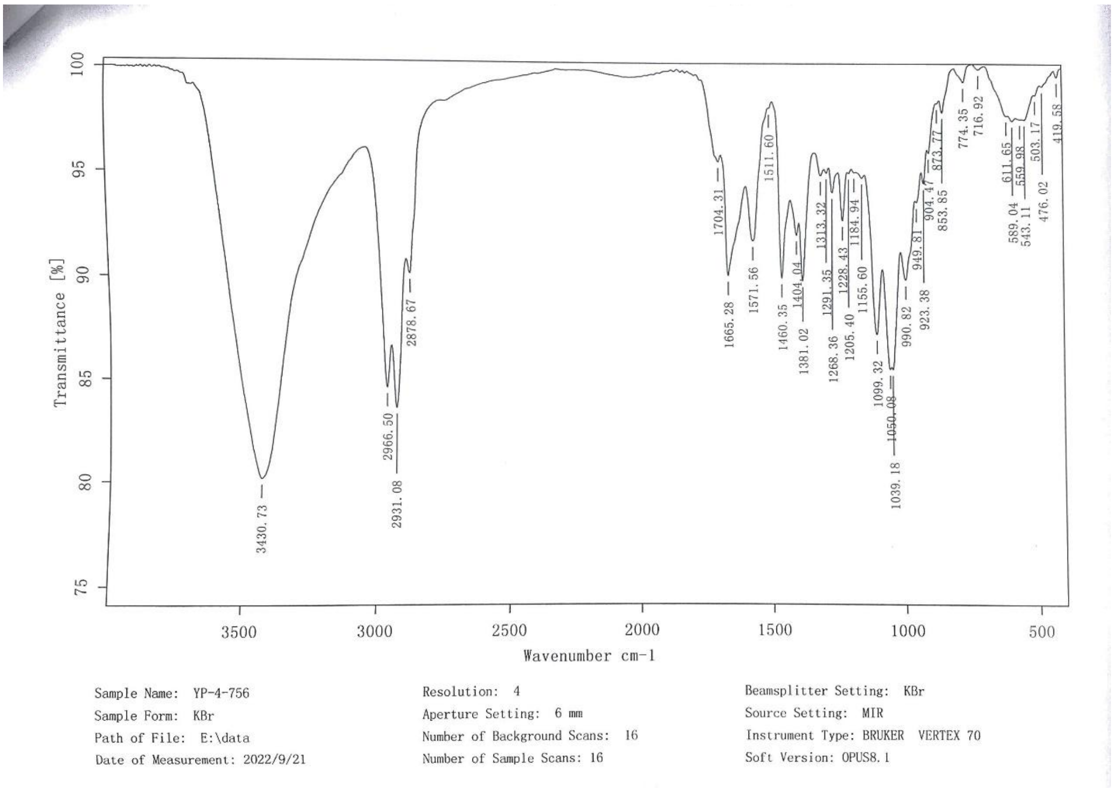

IR spectrum of End-3.

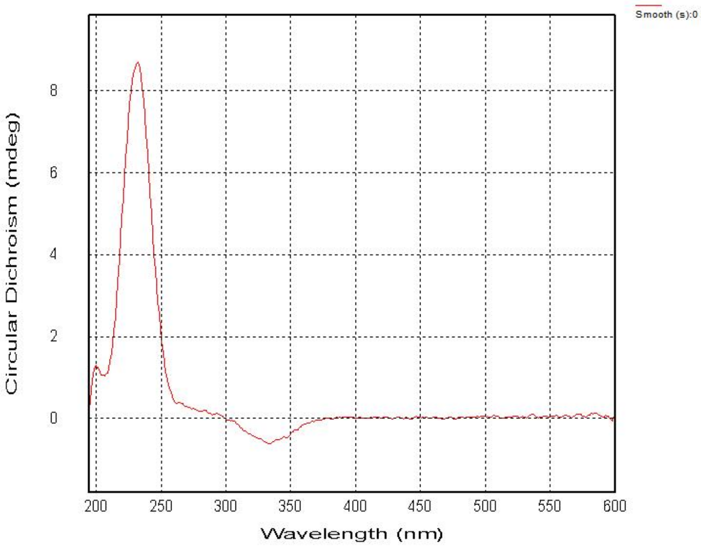

Experimental ECD spectra of End-3.

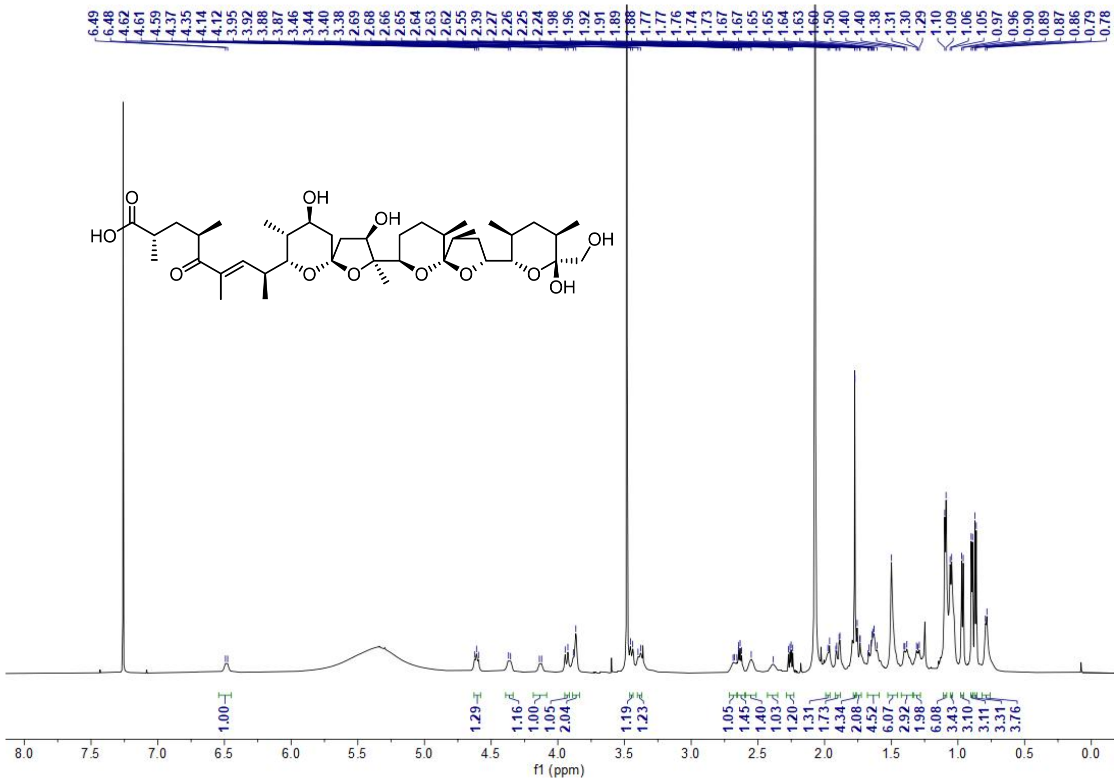

^{1}H NMR spectrum of End-3 in $CDCl_3$.

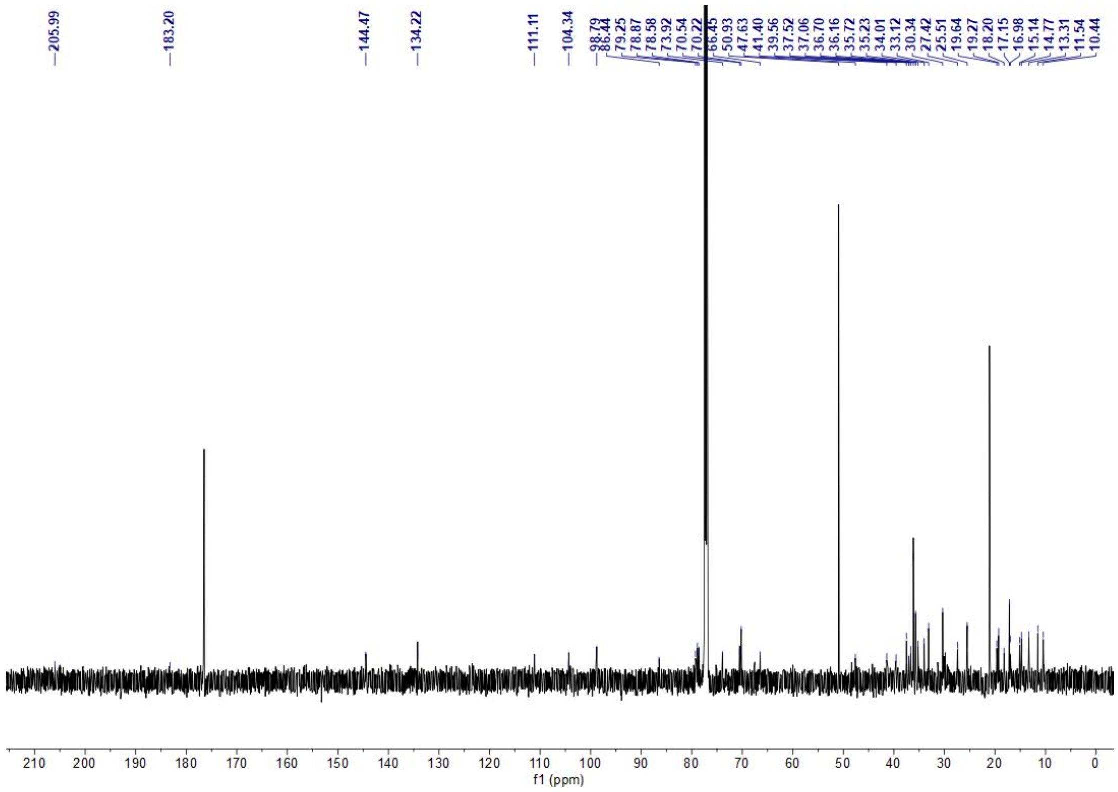

^{13}C NMR spectrum of End-3 in $CDCl_3$.

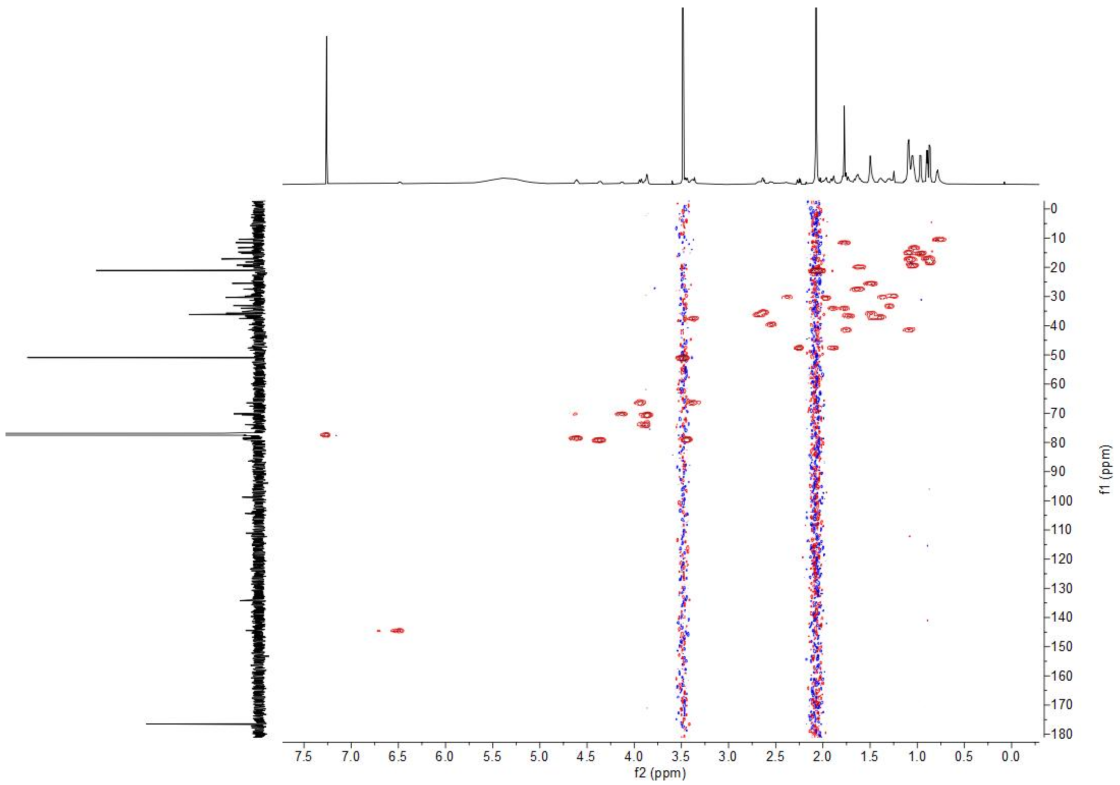

HSQC spectrum of End-3 in $CDCl_3$.

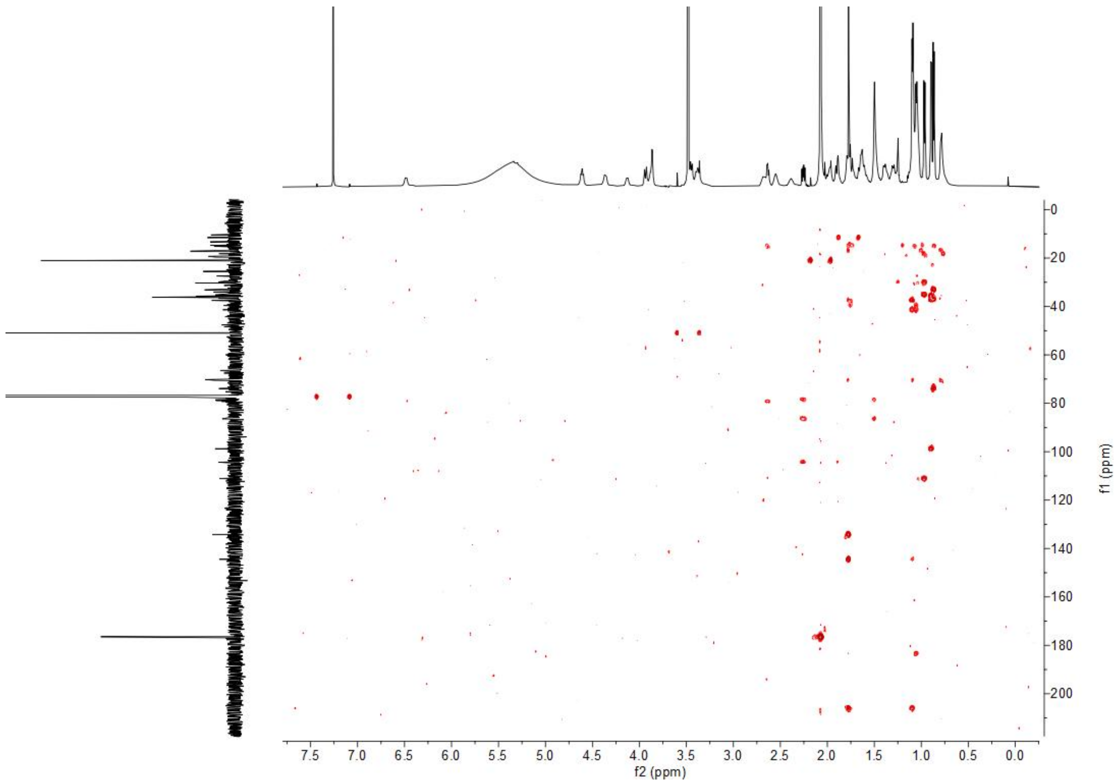

HMBC spectrum of End-3 in $CDCl_3$.

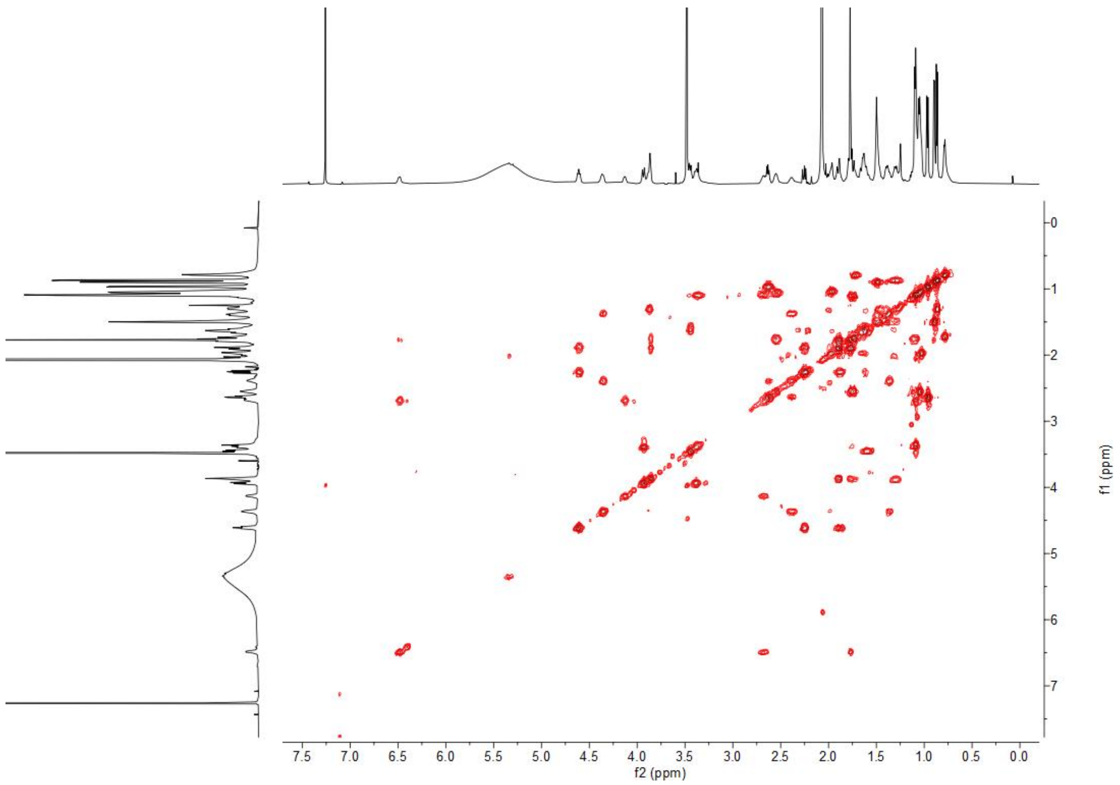

$^1H-^1H$ **COSY spectrum of End-3 in $CDCl_3$.**

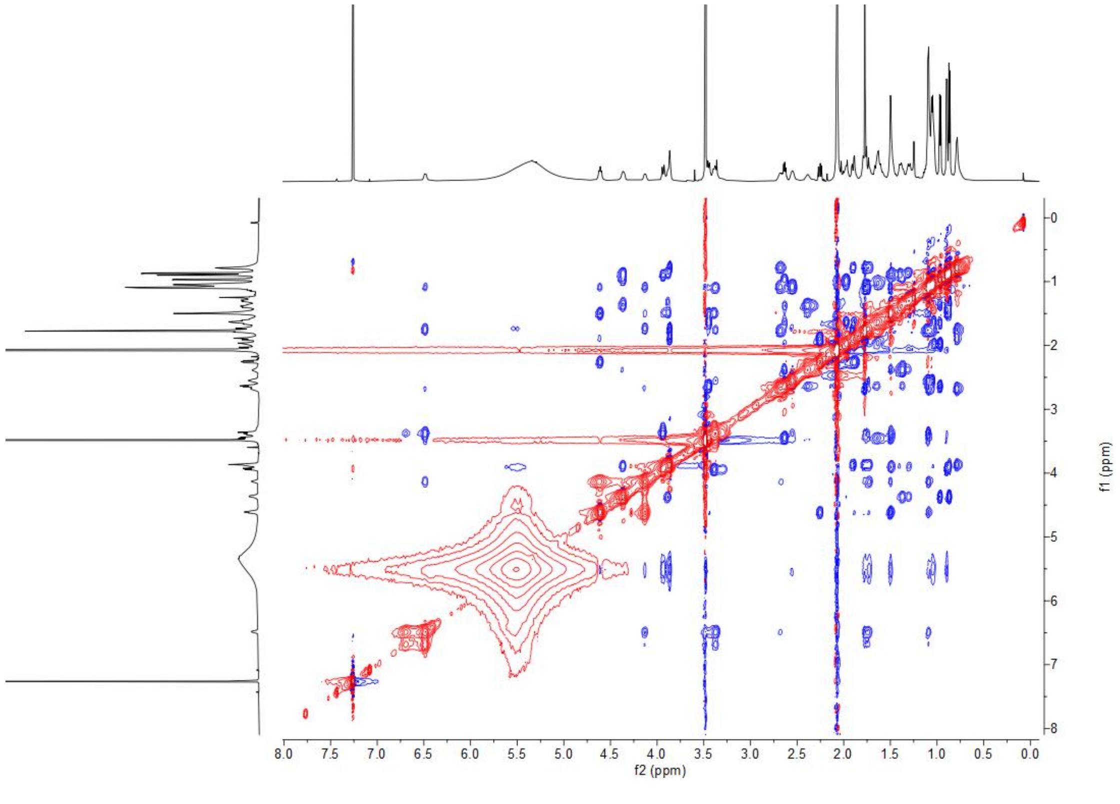

NOESY spectrum of End-3 in $CDCl_3$.

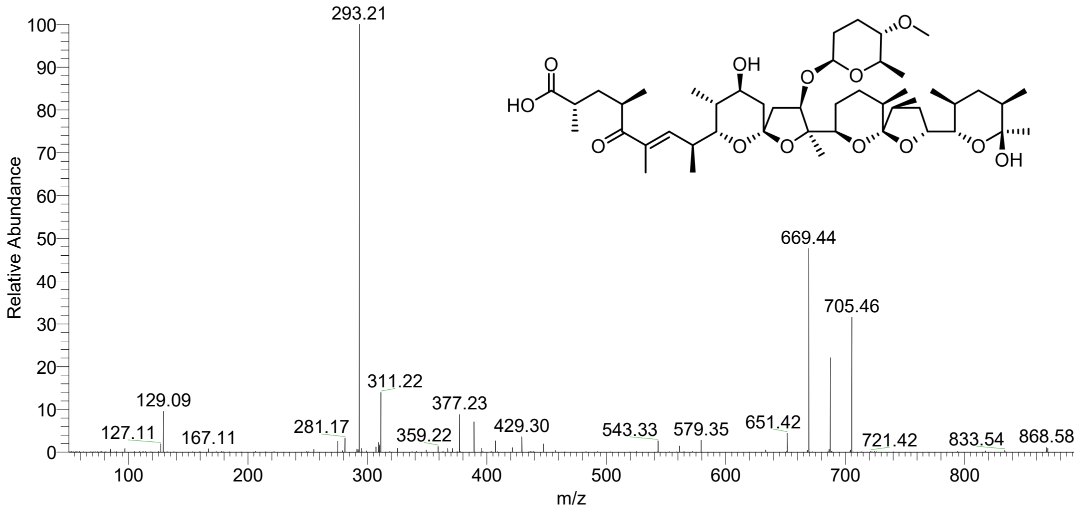

MS² fragmentation spectra of End-4

Rudolph Research Analytical

This sample was measured on an Autopol VI, Serial #91058
Manufactured by Rudolph Research Analytical, Hackettstown, NJ, USA.

Measurement Date : Tuesday, 20-SEP-2022

Set Temperature : OFF

Time Delay : Disabled

Delay between Measurement : Disabled

n	Average	Std.Dev.	% RSD	Maximum	Minimum
5	39.84	0.36	0.90	40.30	39.40

S.No	Sample ID	Time	Result	Scale	OR °Arc	WLG.nm	Lg.mm	Conc.g/100ml	Temp.
1	YP-5-868	02:52:28 PM	40.30	SR	0.0403	589	100.00	0.100	25.4
2	YP-5-868	02:52:36 PM	40.10	SR	0.0401	589	100.00	0.100	25.3
3	YP-5-868	02:52:44 PM	39.40	SR	0.0394	589	100.00	0.100	25.3
4	YP-5-868	02:52:52 PM	39.80	SR	0.0398	589	100.00	0.100	25.3
5	YP-5-868	02:53:01 PM	39.60	SR	0.0396	589	100.00	0.100	25.3

Optical rotation value of End-4.

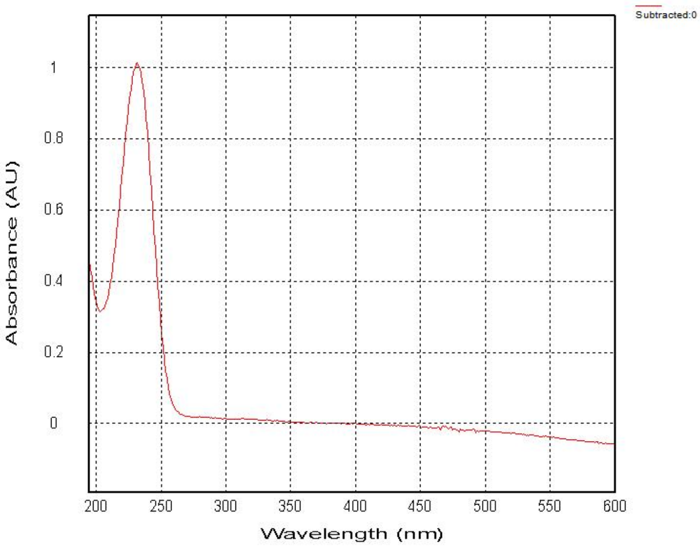

UV spectrum of End-4.

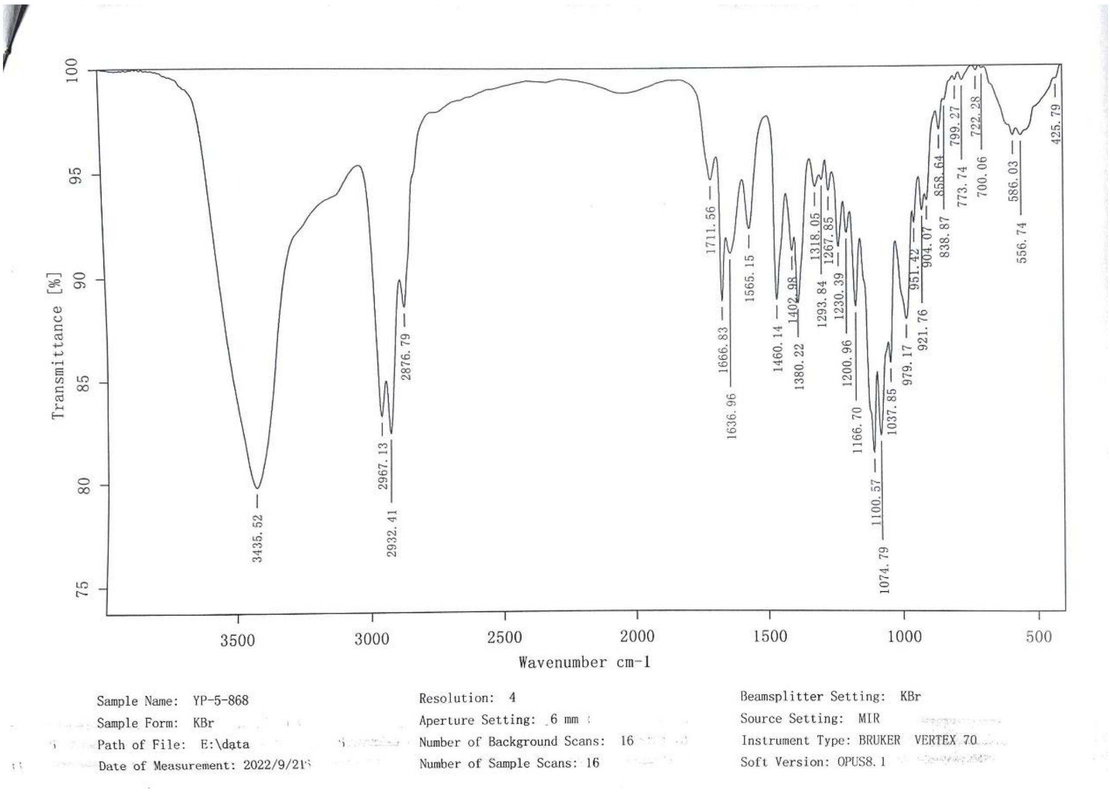

IR spectrum of End-4.

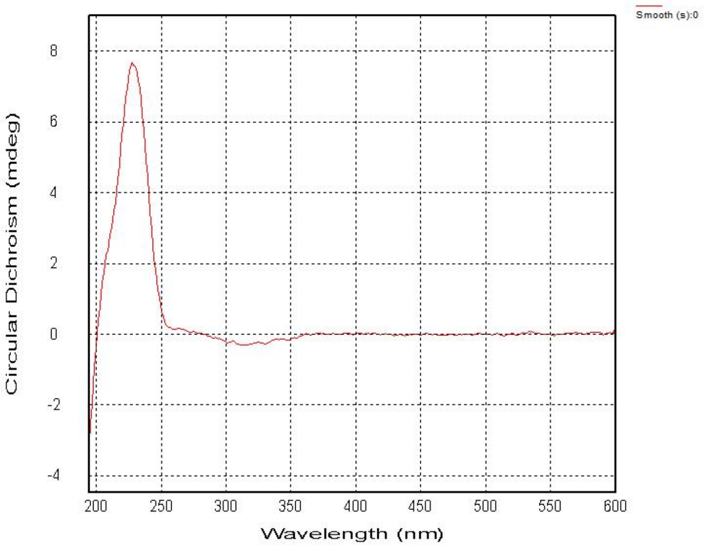

Experimental ECD spectra of End-4.

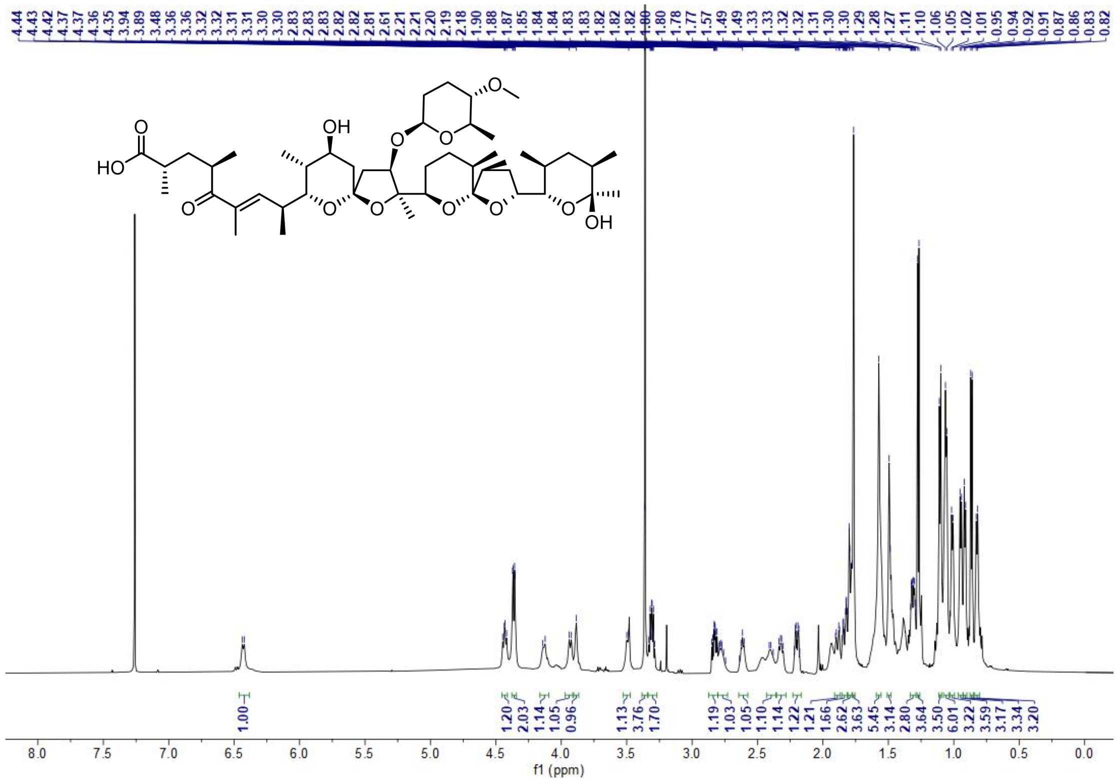

^{1}H NMR spectrum of End-4 in $CDCl_3$.

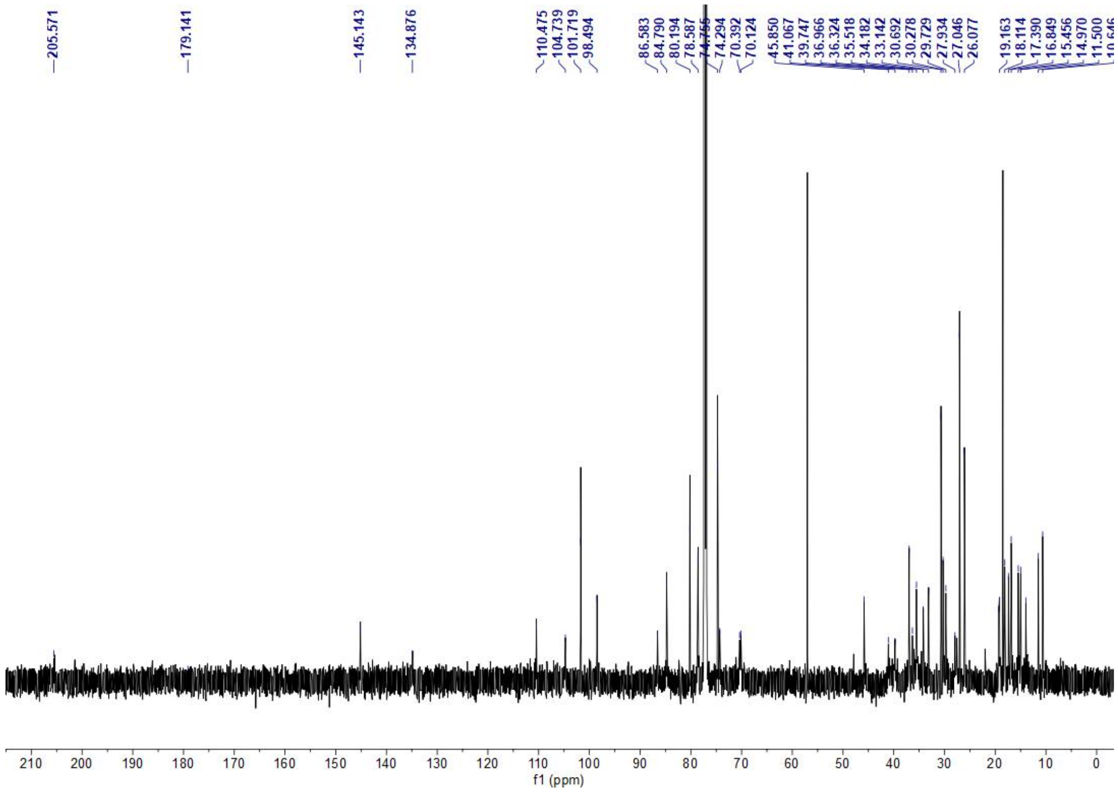

^{13}C NMR spectrum of End-4 in $CDCl_3$.

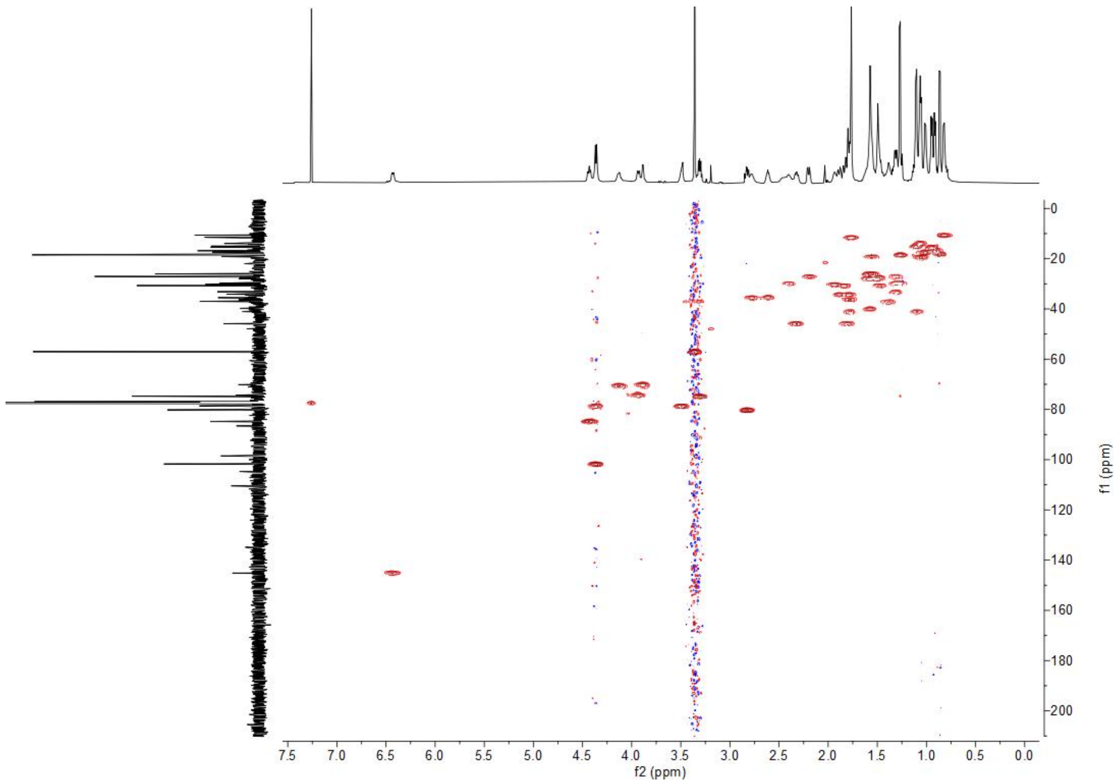

HSQC spectrum of End-4 in $CDCl_3$.

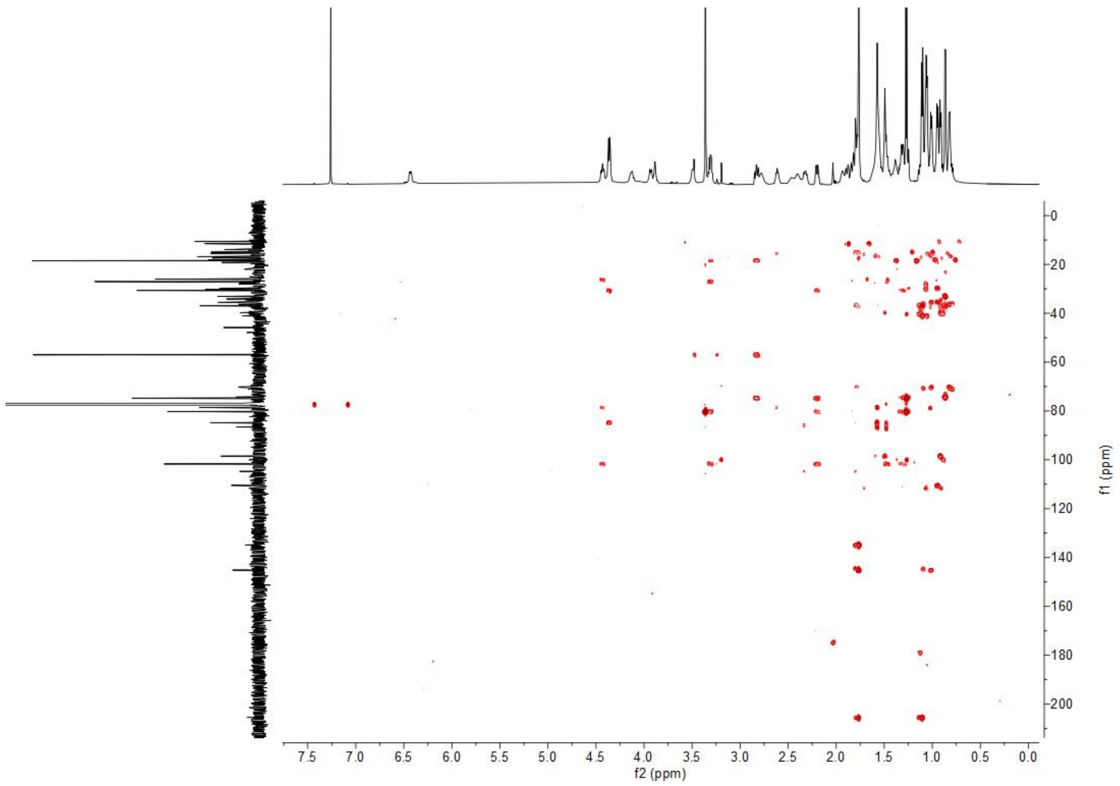

HMBC spectrum of End-4 in $CDCl_3$.

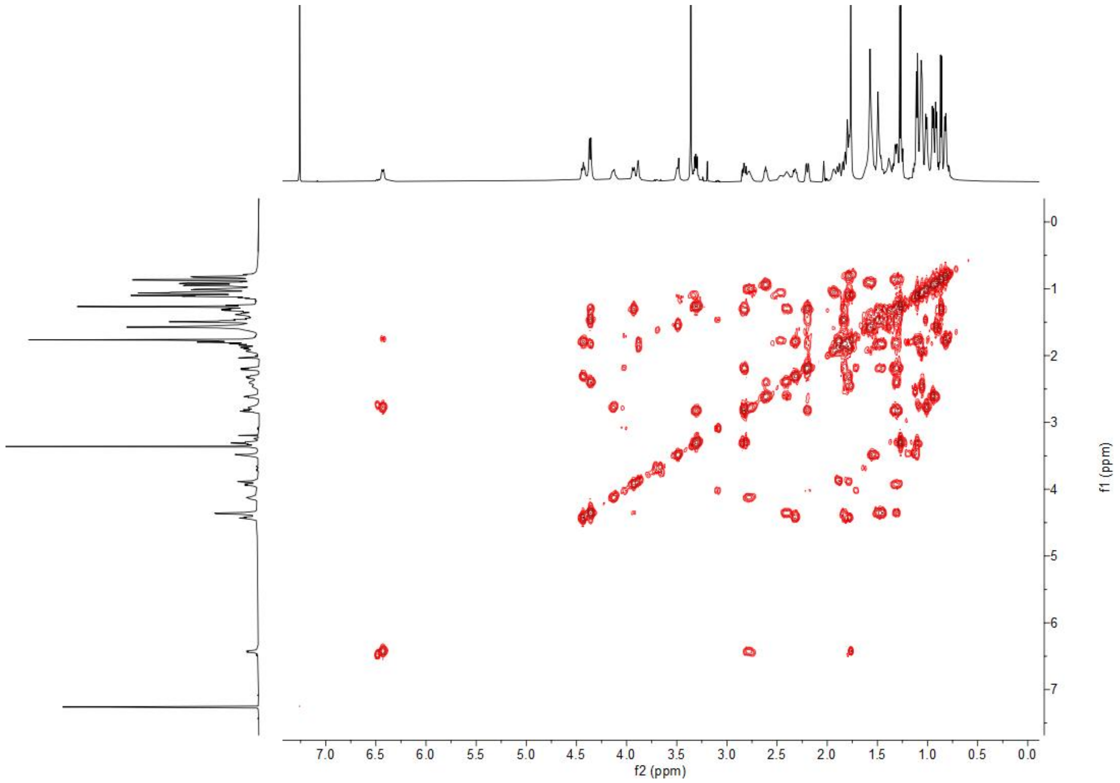

$^1H-^1H$ COSY spectrum of End-4 in $CDCl_3$.

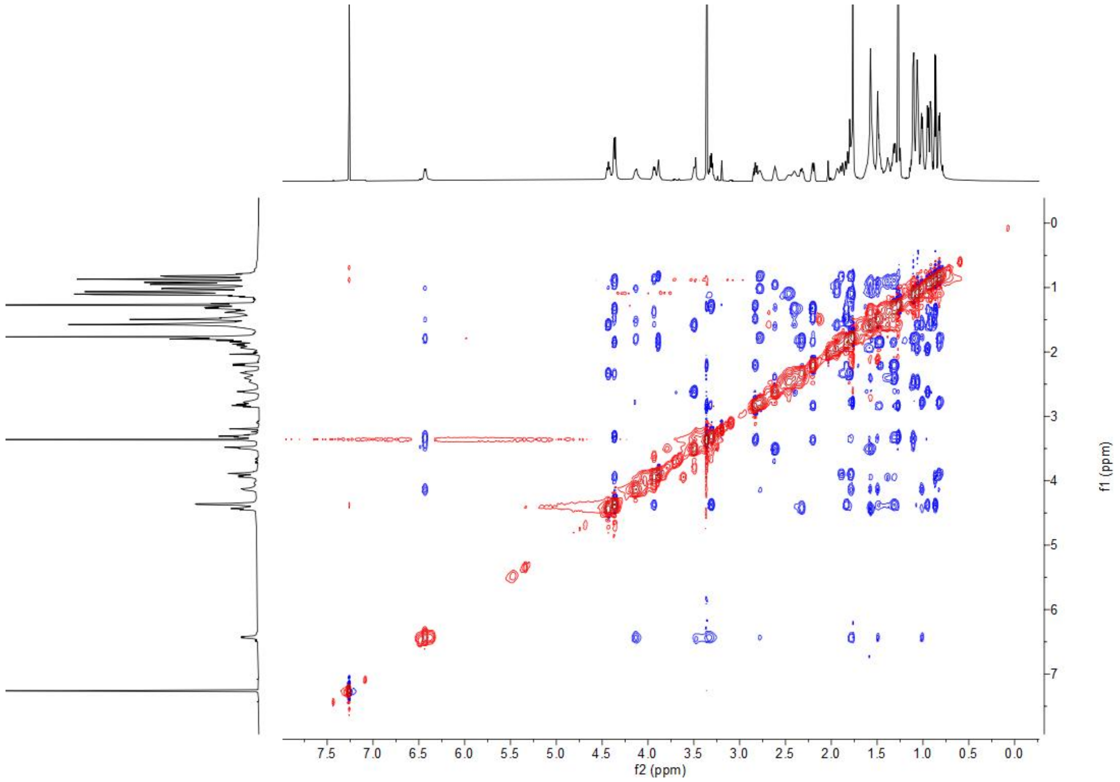

NOESY spectrum of End-4 in CDCl$_3$.

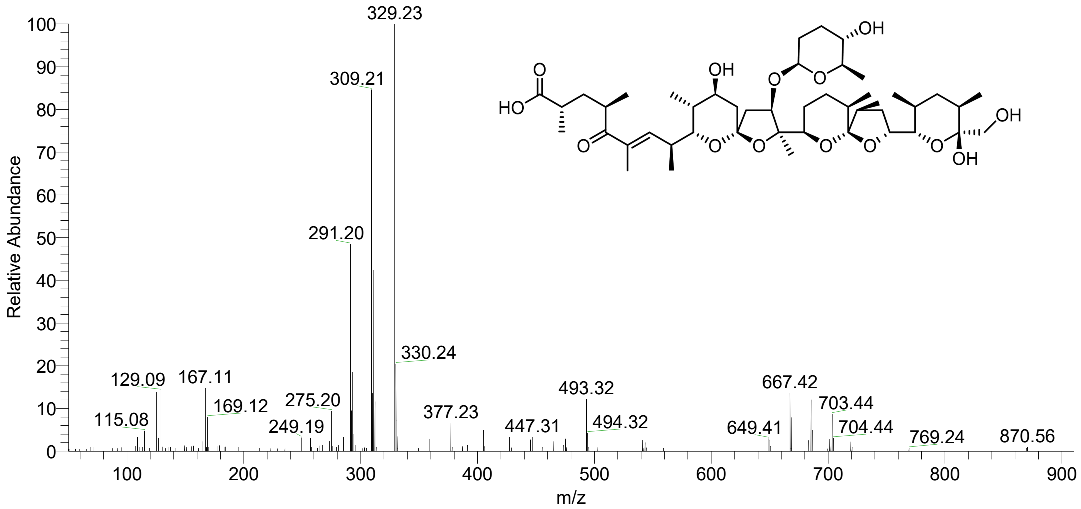

MS^2 fragmentation spectra of End-5.

Rudolph Research Analytical

This sample was measured on an Autopol VI, Serial #91058
Manufactured by Rudolph Research Analytical, Hackettstown, NJ, USA.

Measurement Date : Thursday, 25-AUG-2022

Set Temperature : OFF

Time Delay : Disabled

Delay between Measurement : Disabled

n	Average	Std.Dev.	% RSD	Maximum	Minimum
5	33.72	0.39	1.15	34.30	33.30

S.No	Sample ID	Time	Result	Scale	OR °Arc	WLG.nm	Lg.mm	Conc.g/100ml	Temp.
1	870	12:31:46 PM	34.30	SR	0.0343	589	100.00	0.100	26.6
2	870	12:31:54 PM	33.90	SR	0.0339	589	100.00	0.100	26.5
3	870	12:32:02 PM	33.50	SR	0.0335	589	100.00	0.100	26.5
4	870	12:32:11 PM	33.60	SR	0.0336	589	100.00	0.100	26.5
5	870	12:32:19 PM	33.30	SR	0.0333	589	100.00	0.100	26.5

Optical rotation value of End-5.

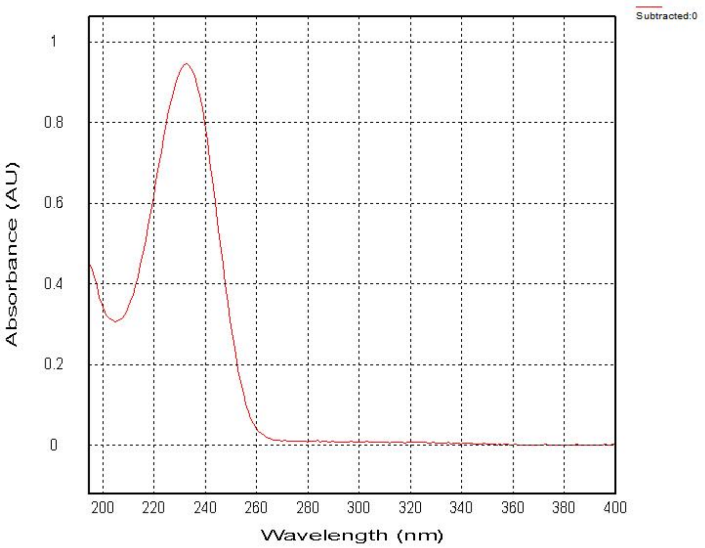

UV spectrum of End-5.

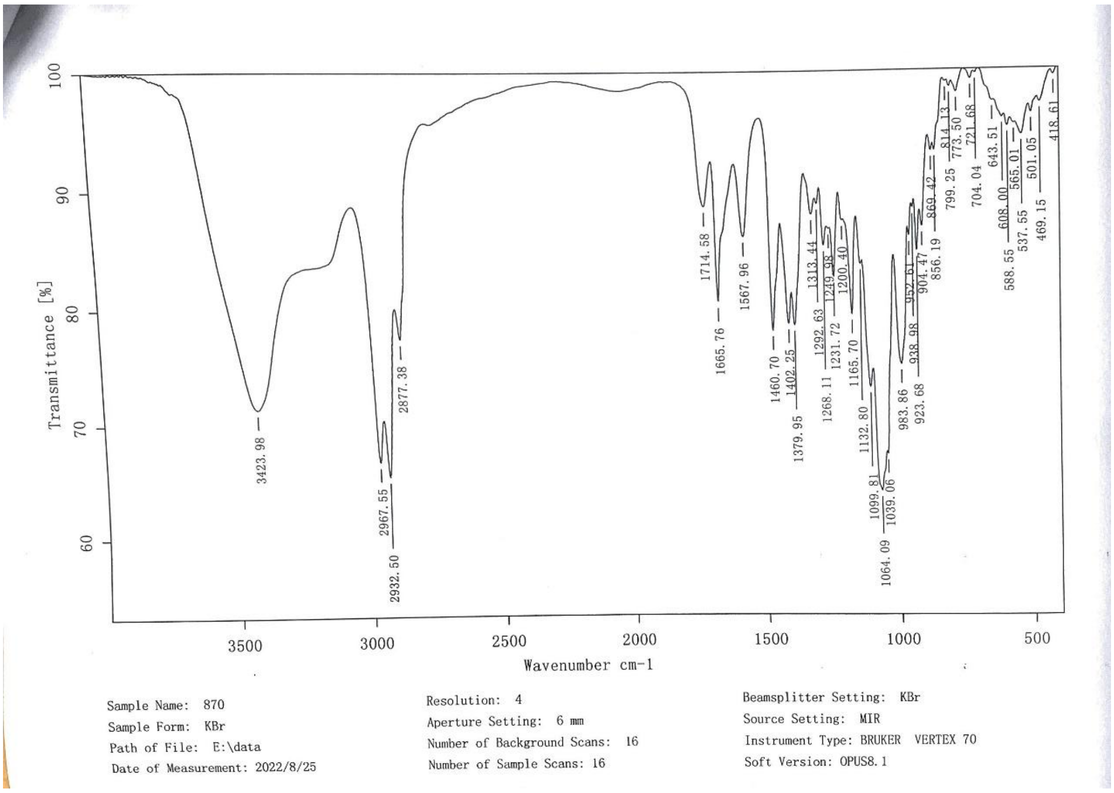

IR spectrum of End-5.

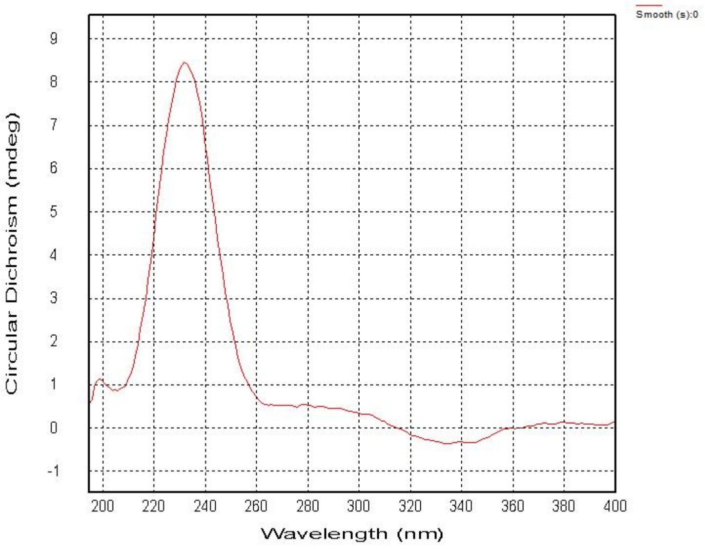

Experimental ECD spectra of End-5.

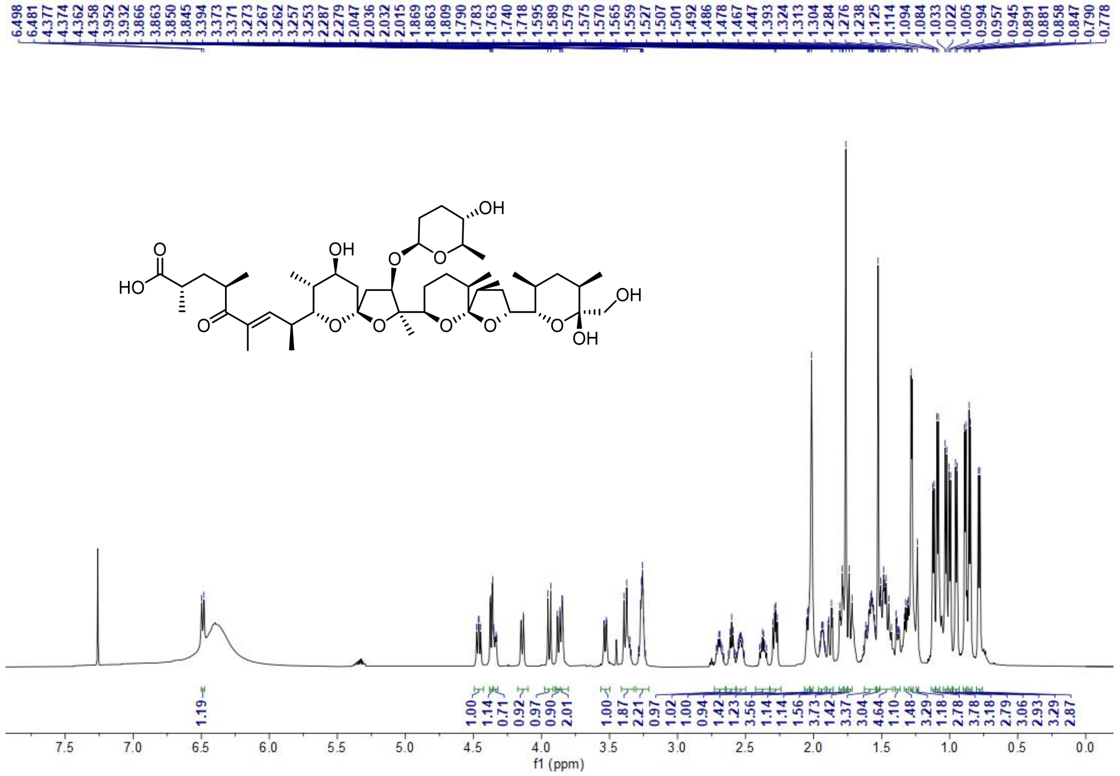

^{1}H NMR spectrum of End-5 in $CDCl_3$.

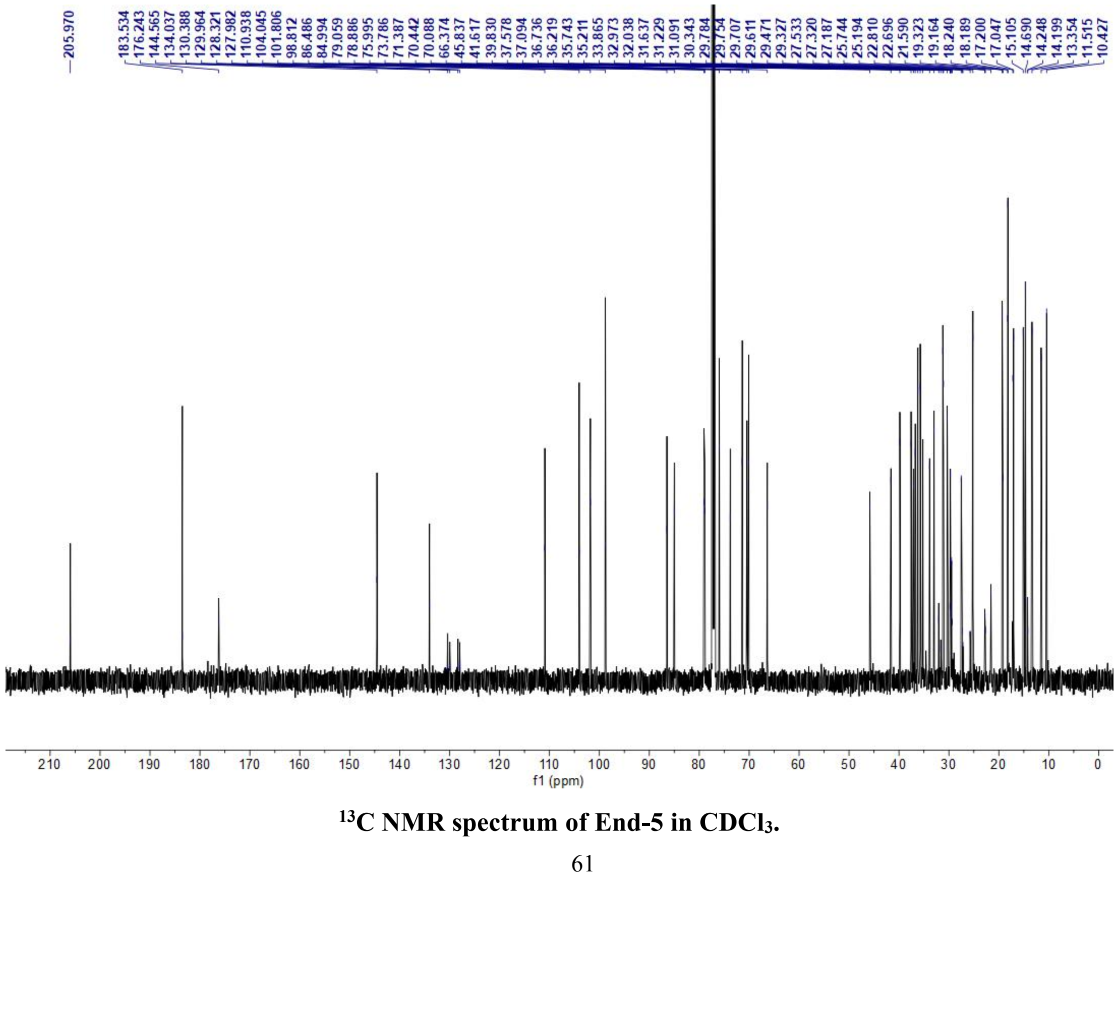

^{13}C NMR spectrum of End-5 in $CDCl_3$.

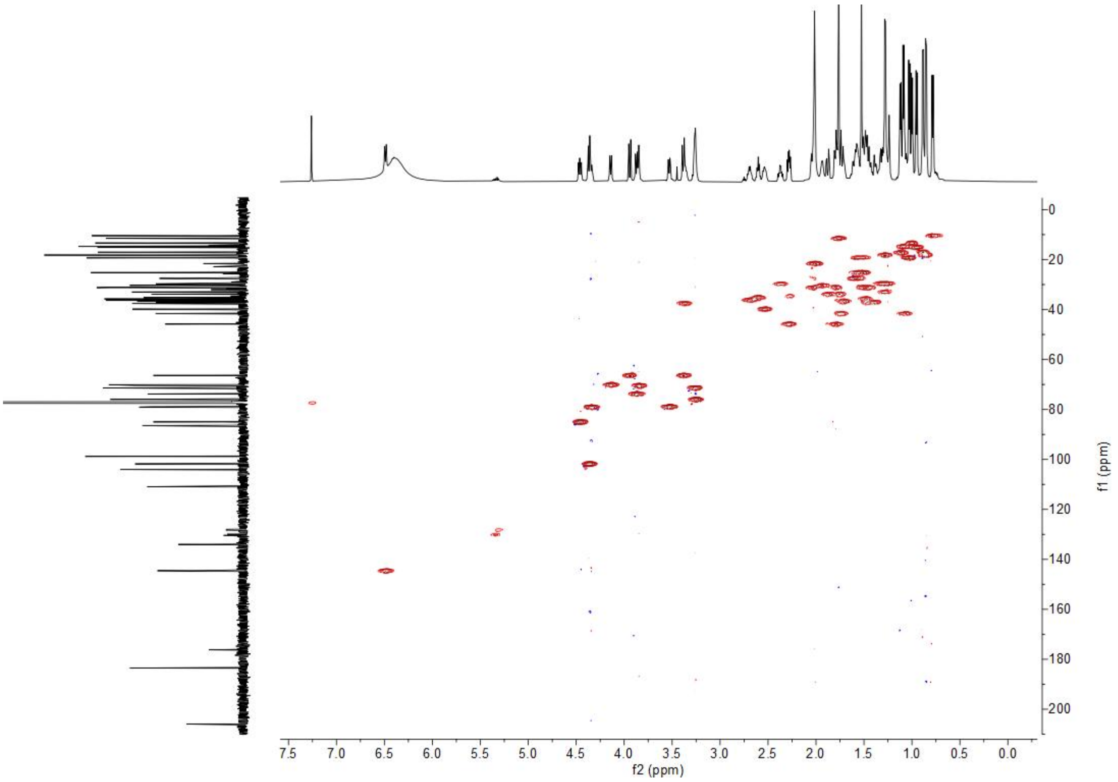

HSQC spectrum of End-5 in $CDCl_3$

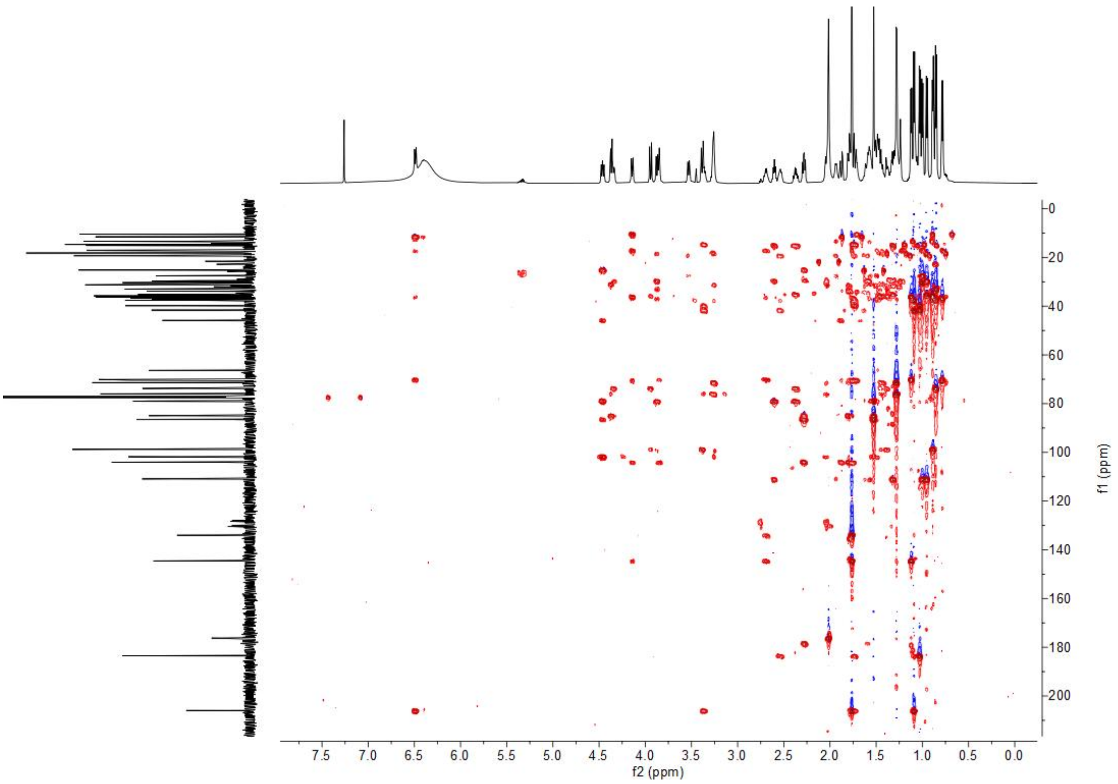

HMBC spectrum of End-5 in $CDCl_3$.

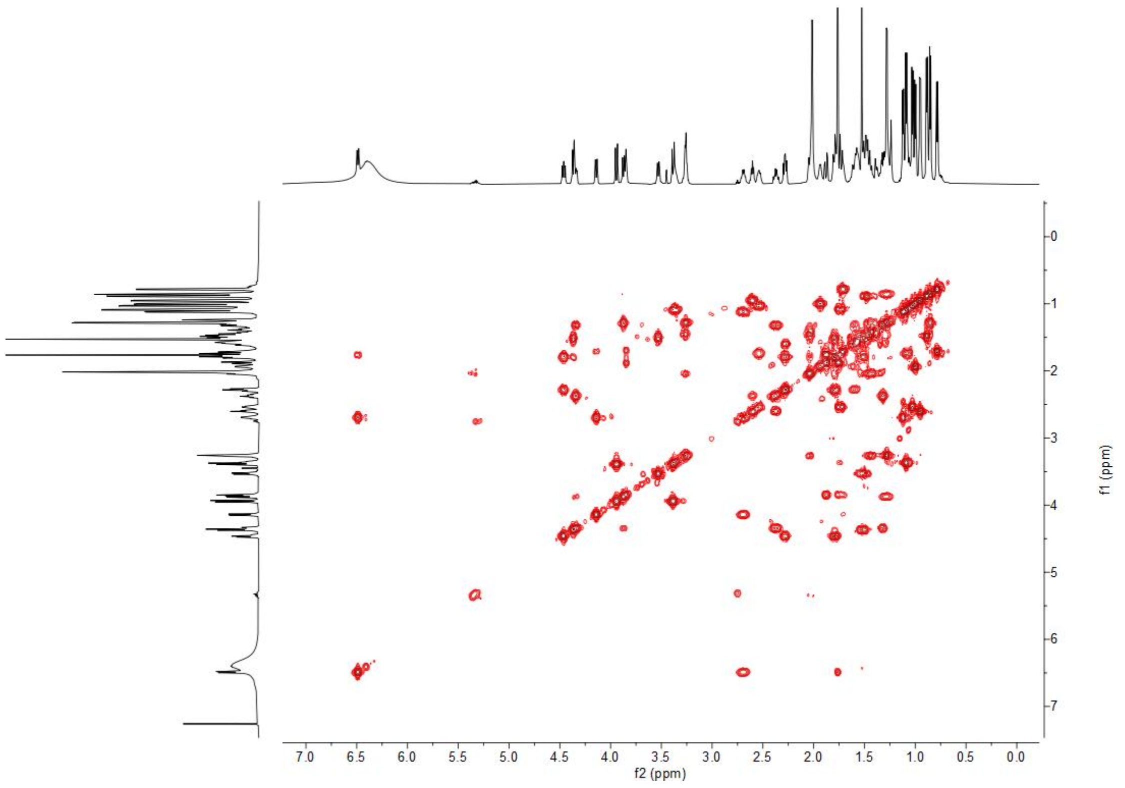

$^1H-^1H$ COSY spectrum of End-5 in $CDCl_3$.

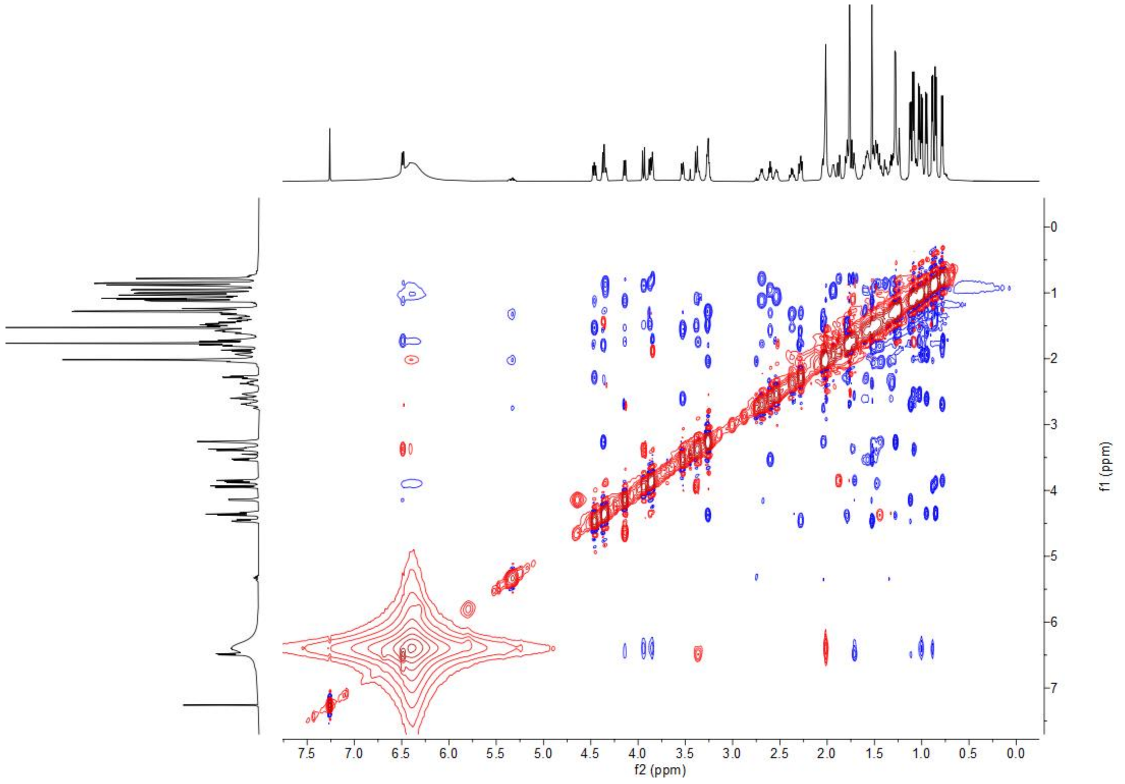

NOESY spectrum of End-5 in $CDCl_3$.

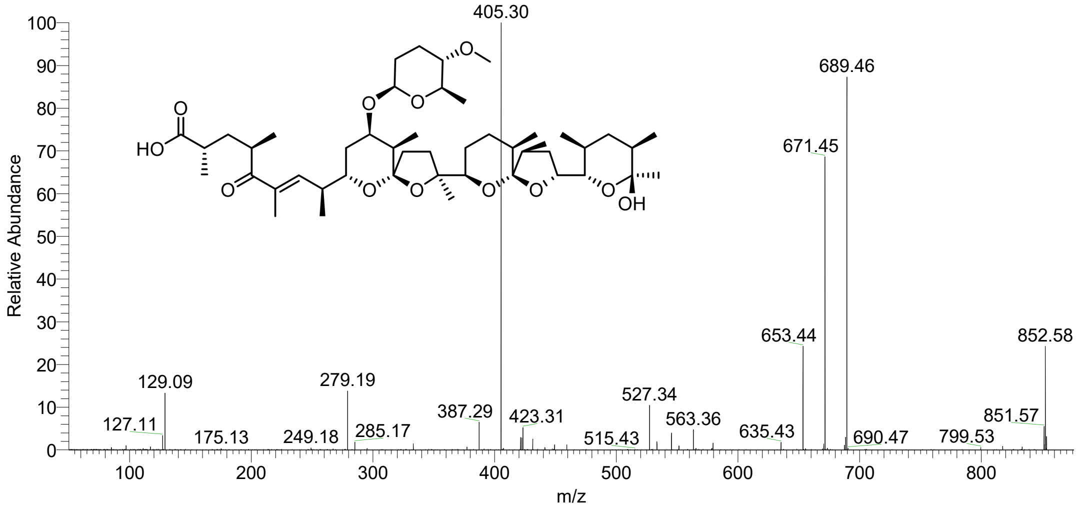

MS^2 fragmentation spectra of Len-10.

Rudolph Research Analytical

This sample was measured on an Autopol VI, Serial #91058
Manufactured by Rudolph Research Analytical, Hackettstown, NJ, USA.

Measurement Date : Tuesday, 20-SEP-2022

Set Temperature : OFF

Time Delay : Disabled

Delay between Measurement : Disabled

n	Average	Std.Dev.	% RSD	Maximum	Minimum
5	32.80	1.10	3.35	34.00	32.00

S.No	Sample ID	Time	Result	Scale	OR °Arc	WLG.nm	Lg.mm	Conc.g/100ml	Temp.
1	YP-10	06:36:56 PM	34.00	SR	0.034	589	100.00	0.100	25.1
2	YP-10	06:37:02 PM	32.00	SR	0.032	589	100.00	0.100	25.1
3	YP-10	06:37:08 PM	32.00	SR	0.032	589	100.00	0.100	25.1
4	YP-10	06:37:14 PM	32.00	SR	0.032	589	100.00	0.100	25.1
5	YP-10	06:37:21 PM	34.00	SR	0.034	589	100.00	0.100	25.0

Optical rotation value of Len-10.

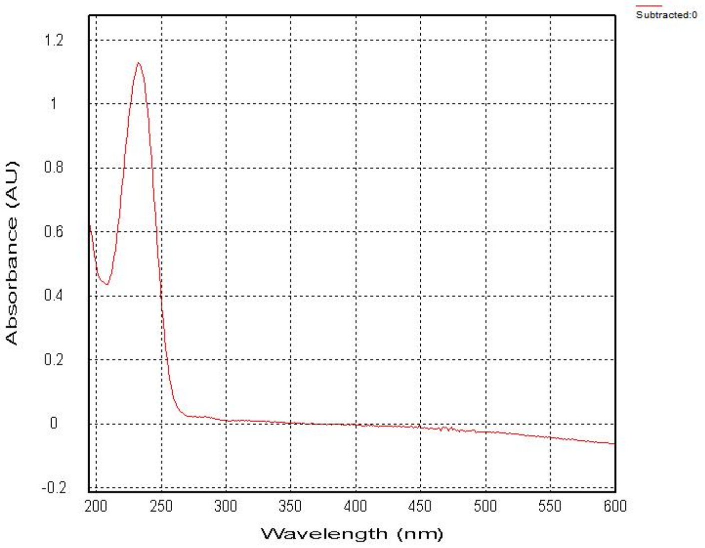

UV spectrum of Len-10.

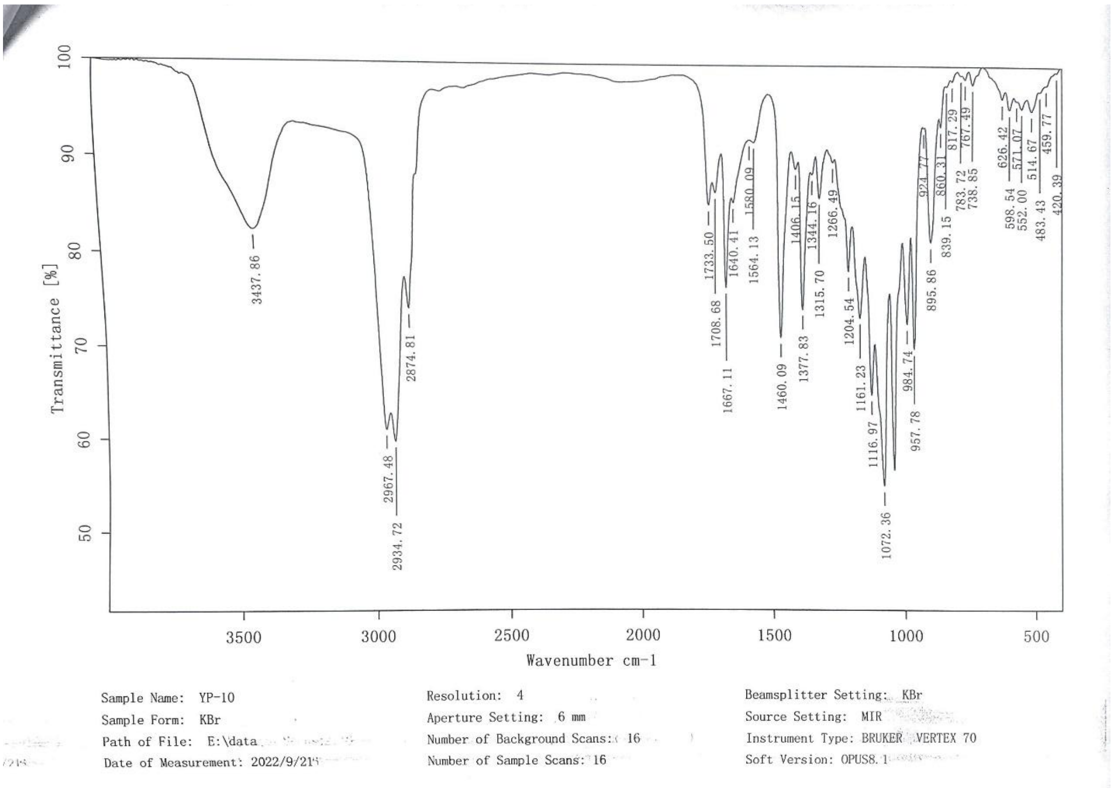

IR spectrum of Len-10.

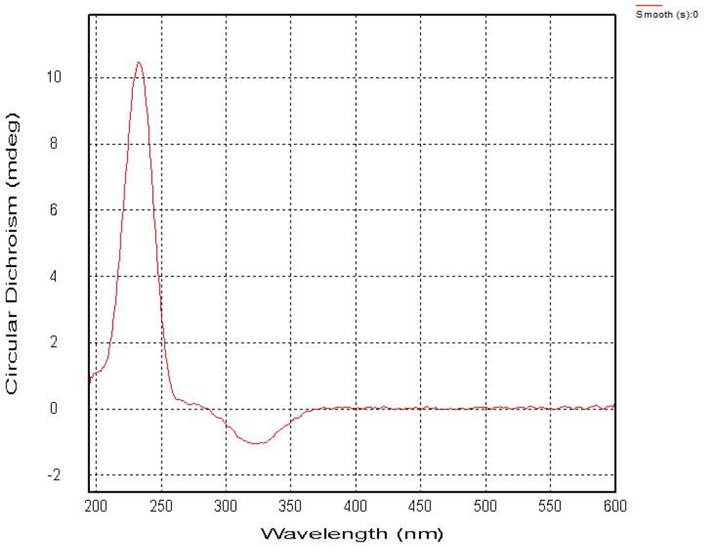

Experimental ECD spectra of Len-10.

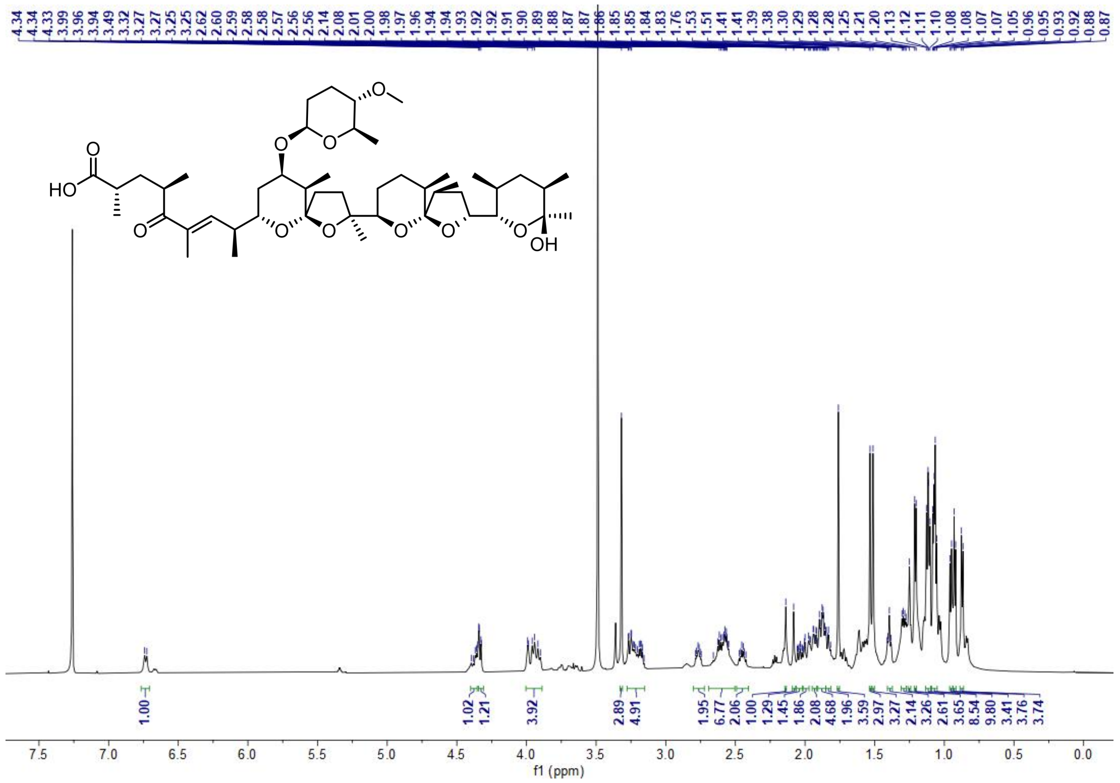

^{1}H NMR spectrum of Len-10 in $CDCl_3$.

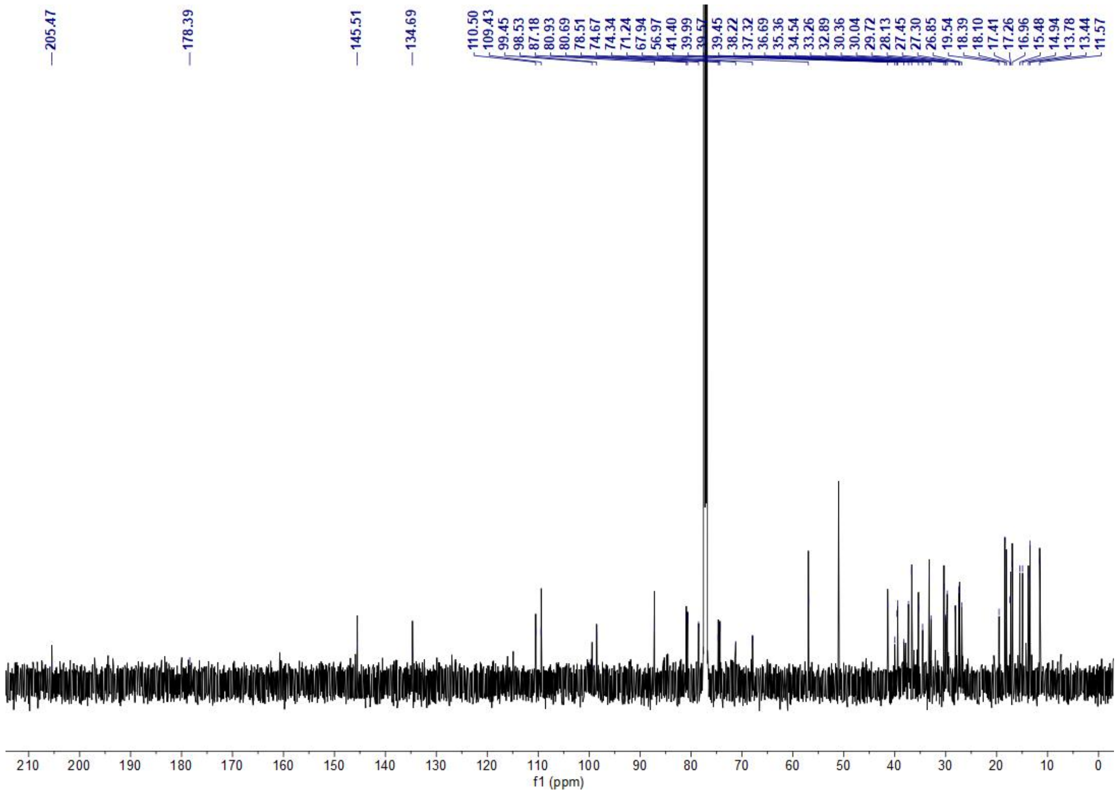

^{13}C NMR spectrum of Len-10 in $CDCl_3$.

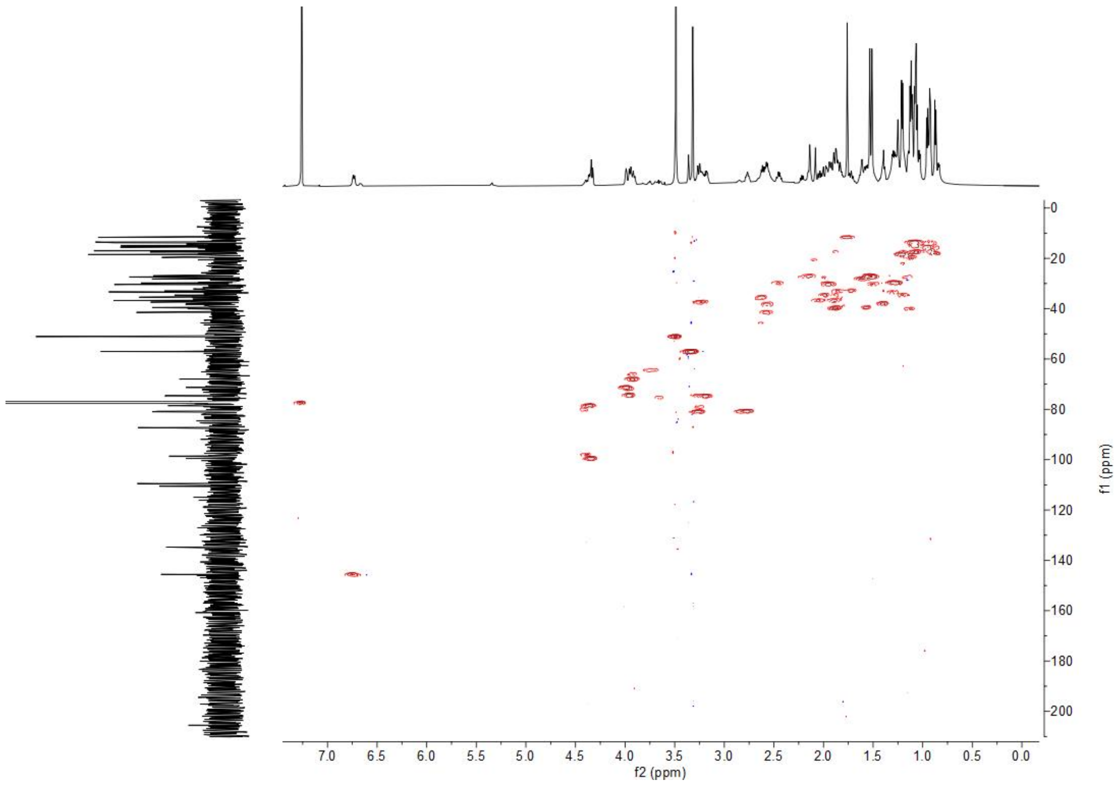

HSQC spectrum of Len-10 in $CDCl_3$.

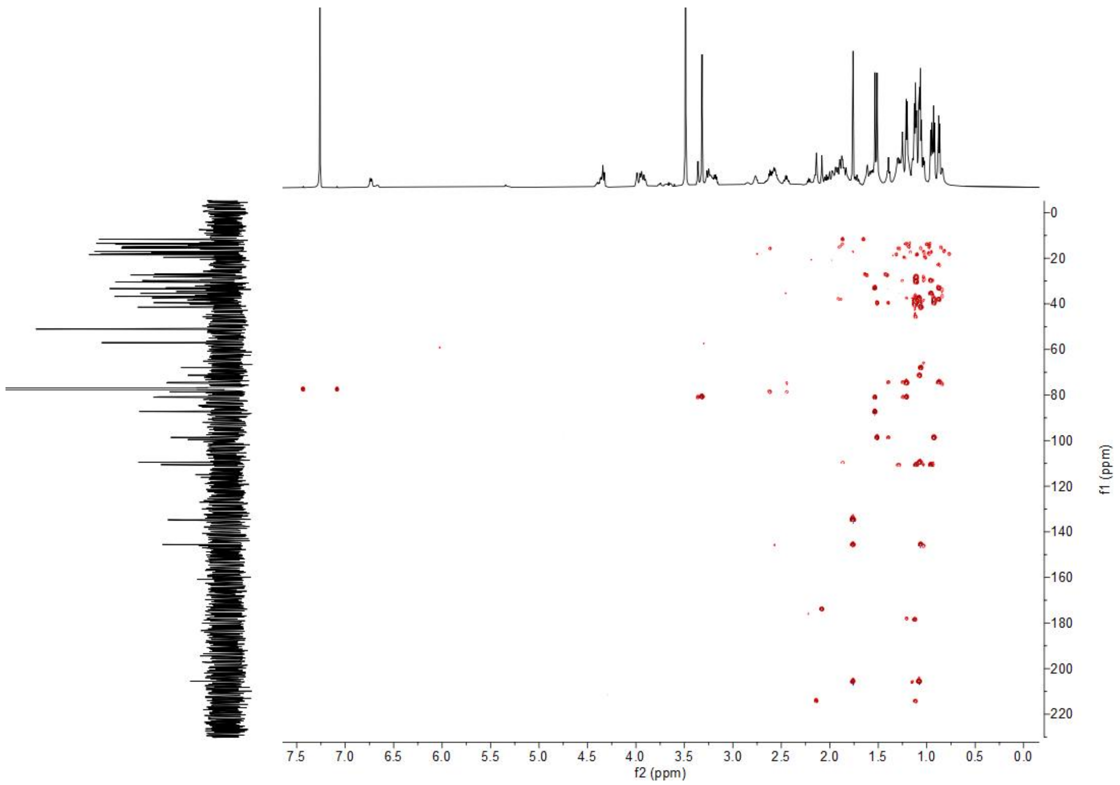

HMBC spectrum of Len-10 in $CDCl_3$.

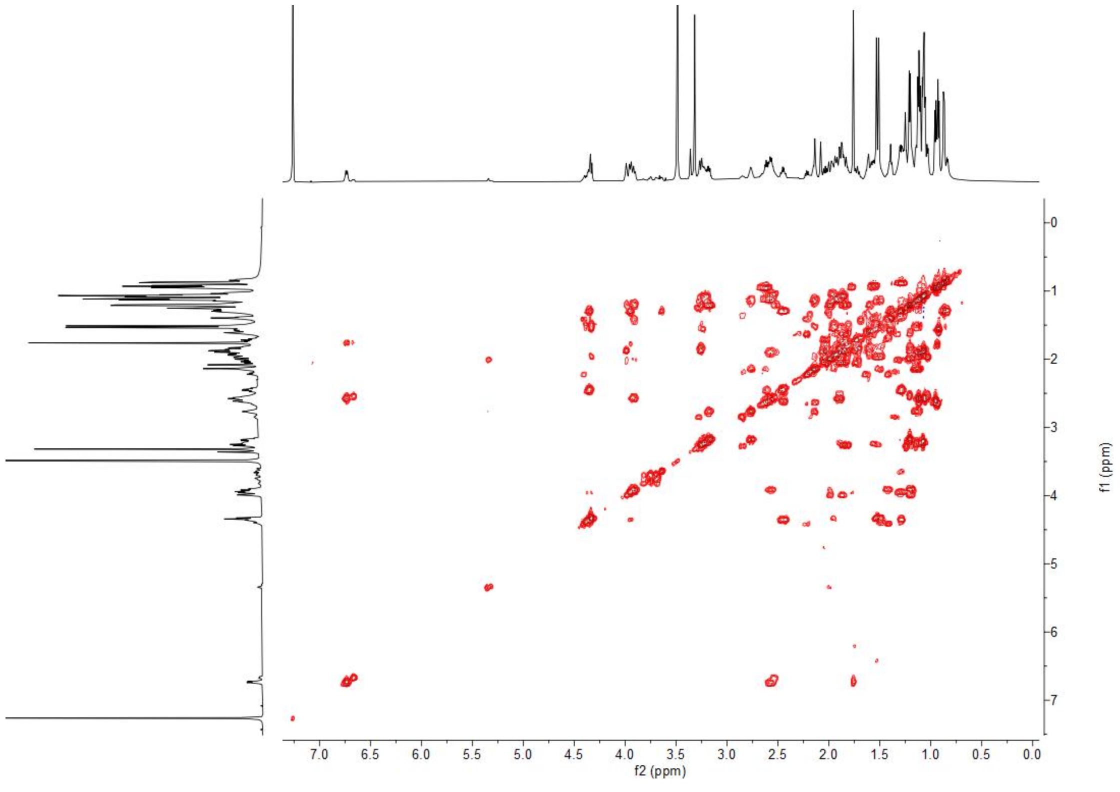

$^1H-^1H$ COSY spectrum of Len-10 in $CDCl_3$.

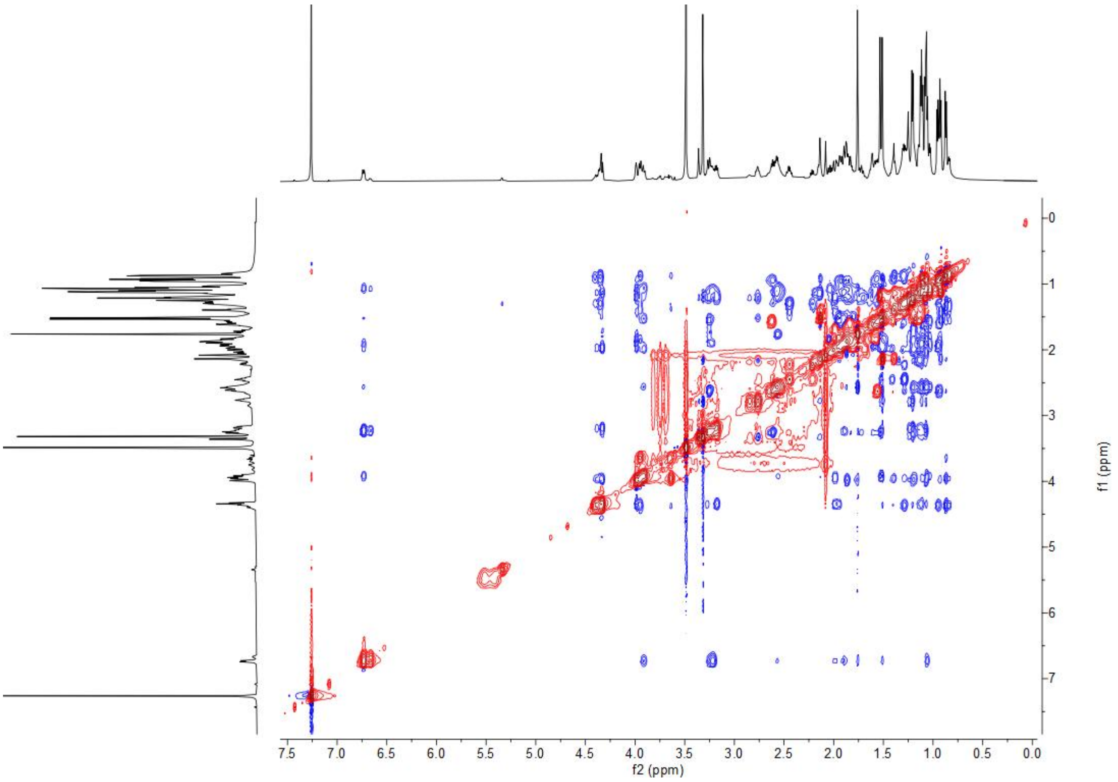

NOESY spectrum of Len-10 in $CDCl_3$.

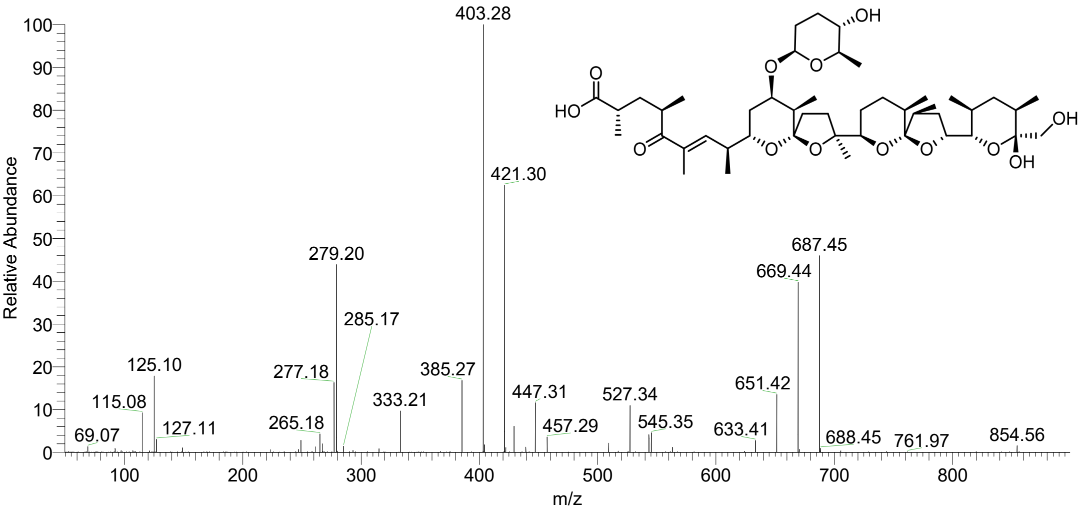

MS^2 fragmentation spectra of Len-11.

Rudolph Research Analytical

This sample was measured on an Autopol VI, Serial #91058
Manufactured by Rudolph Research Analytical, Hackettstown, NJ, USA.

Measurement Date : Tuesday, 20-SEP-2022

Set Temperature : OFF

Time Delay : Disabled

Delay between Measurement : Disabled

n	Average	Std.Dev.	% RSD	Maximum	Minimum
5	34.84	0.23	0.66	35.10	34.60

S.No	Sample ID	Time	Result	Scale	OR °Arc	WLG.nm	Lg.mm	Conc.g/100ml	Temp.
1	YP-8-854	02:45:30 PM	35.10	SR	0.0351	589	100.00	0.100	25.3
2	YP-8-854	02:45:39 PM	34.90	SR	0.0349	589	100.00	0.100	25.3
3	YP-8-854	02:45:47 PM	34.60	SR	0.0346	589	100.00	0.100	25.3
4	YP-8-854	02:45:55 PM	34.60	SR	0.0346	589	100.00	0.100	25.3
5	YP-8-854	02:46:03 PM	35.00	SR	0.0350	589	100.00	0.100	25.3

Optical rotation value of Len-11.

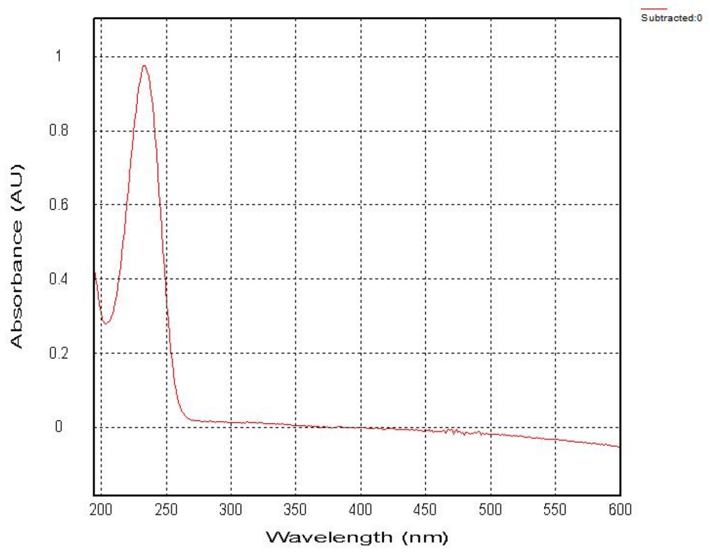

UV spectrum of Len-11.

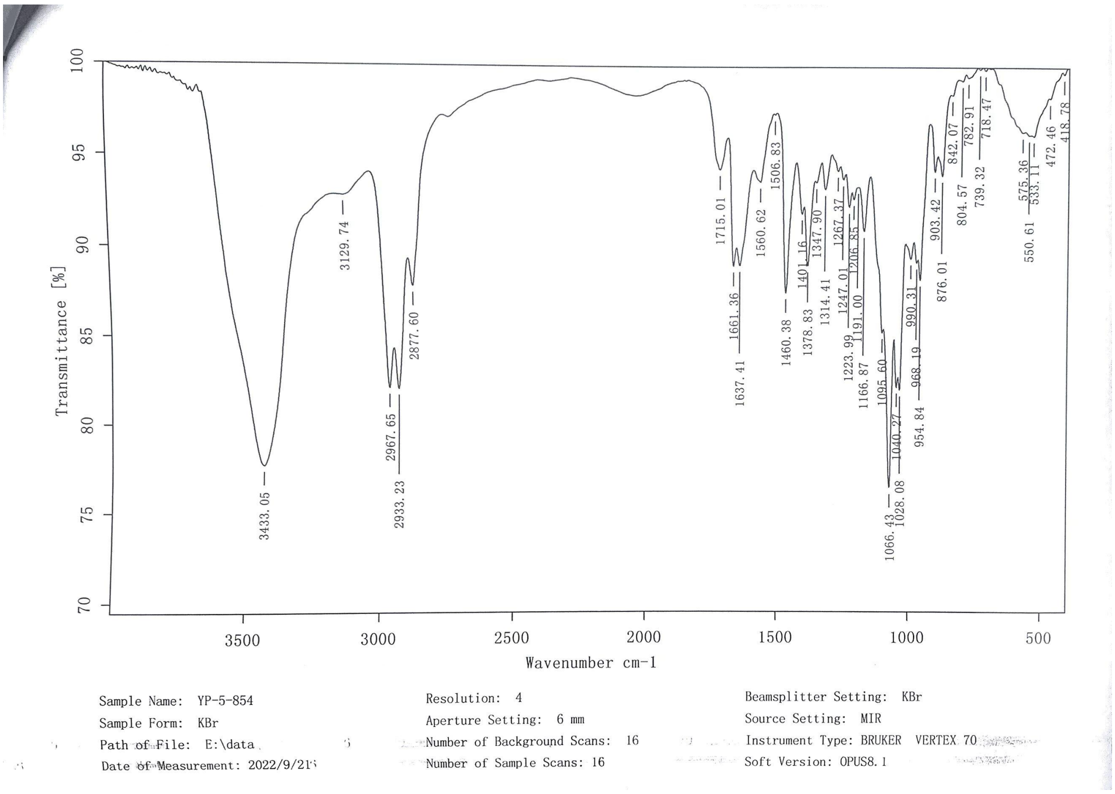

IR spectrum of Len-11.

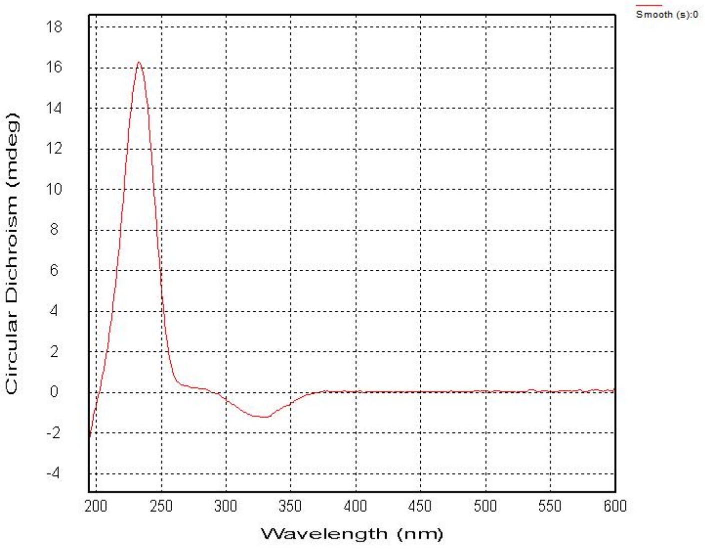

Experimental ECD spectra of Len-11.

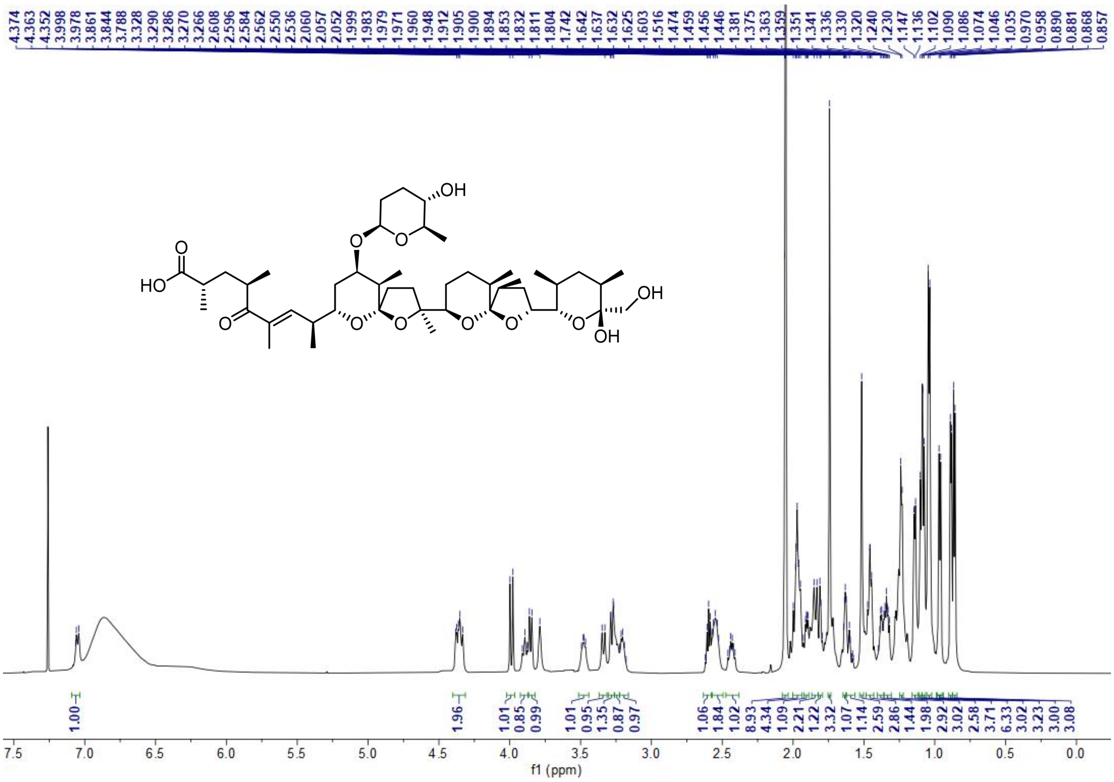

^{1}H NMR spectrum of Len-11 in $CDCl_3$.

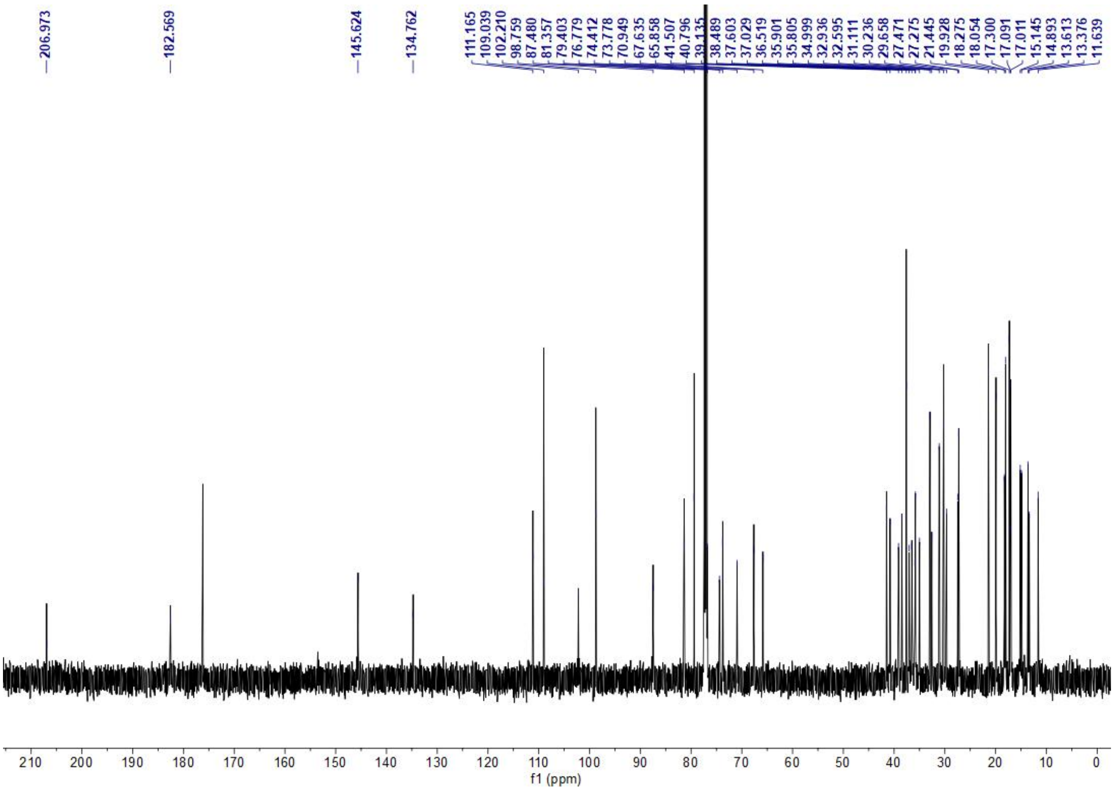

^{13}C NMR spectrum of Len-11 in $CDCl_3$.

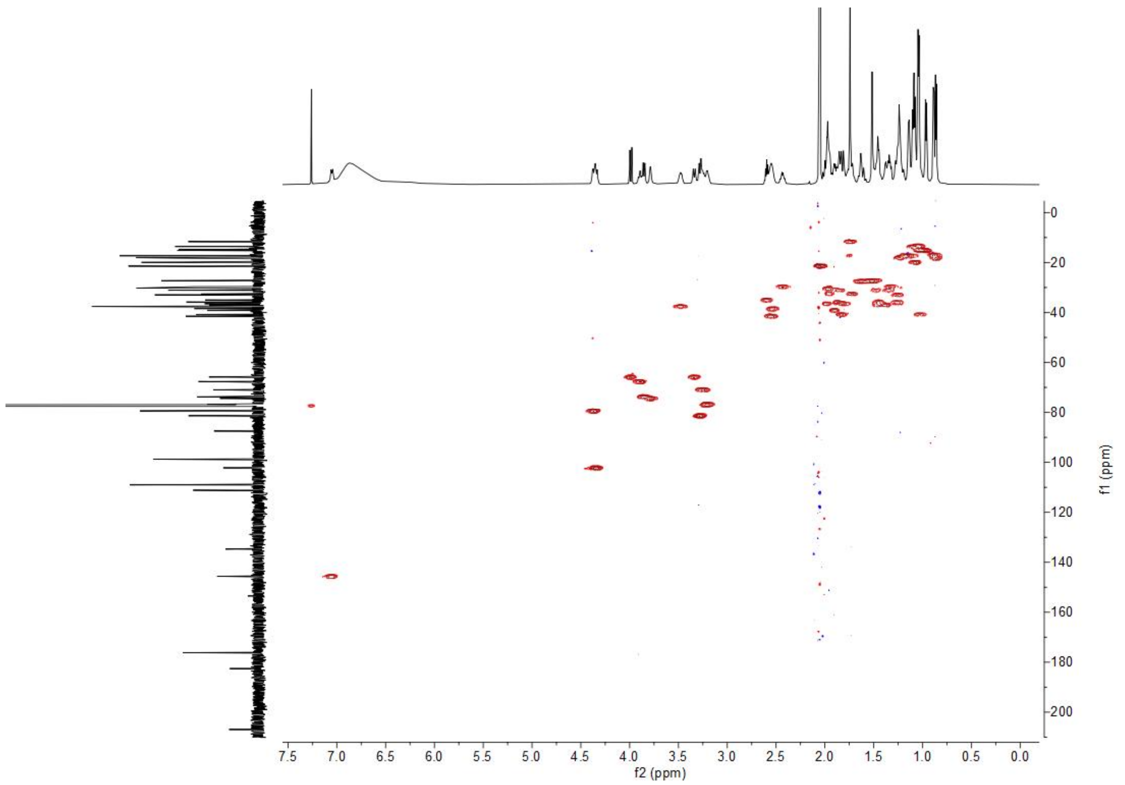

HSQC spectrum of Len-11 in $CDCl_3$.

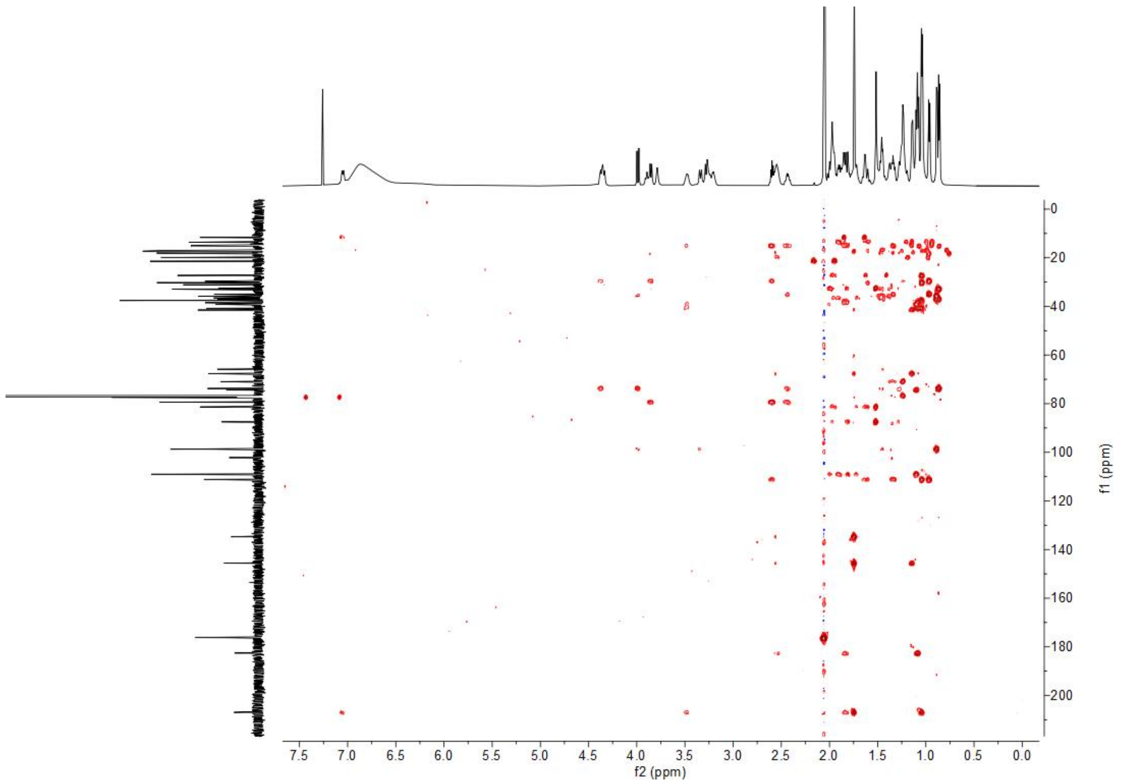

HMBC spectrum of Len-11 in $CDCl_3$.

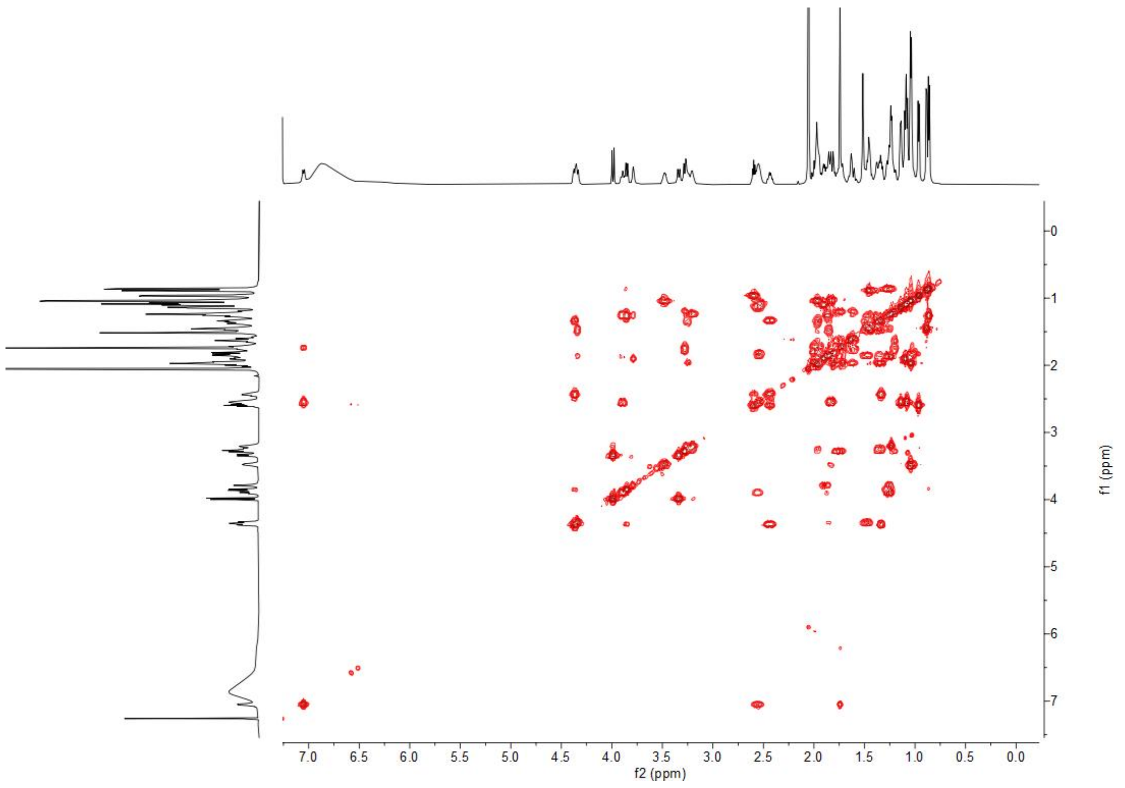

^{1}H−^{1}H COSY spectrum of Len-11 in $CDCl_3$.

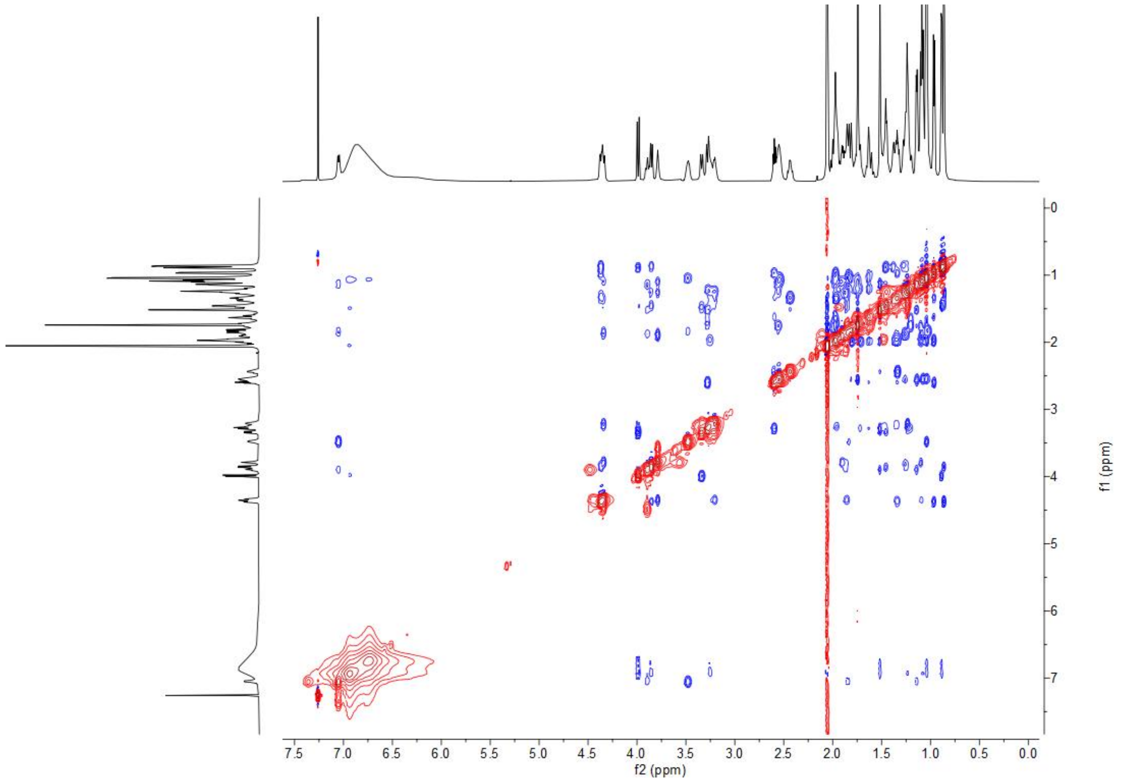

NOESY spectrum of Len-11 in $CDCl_3$.

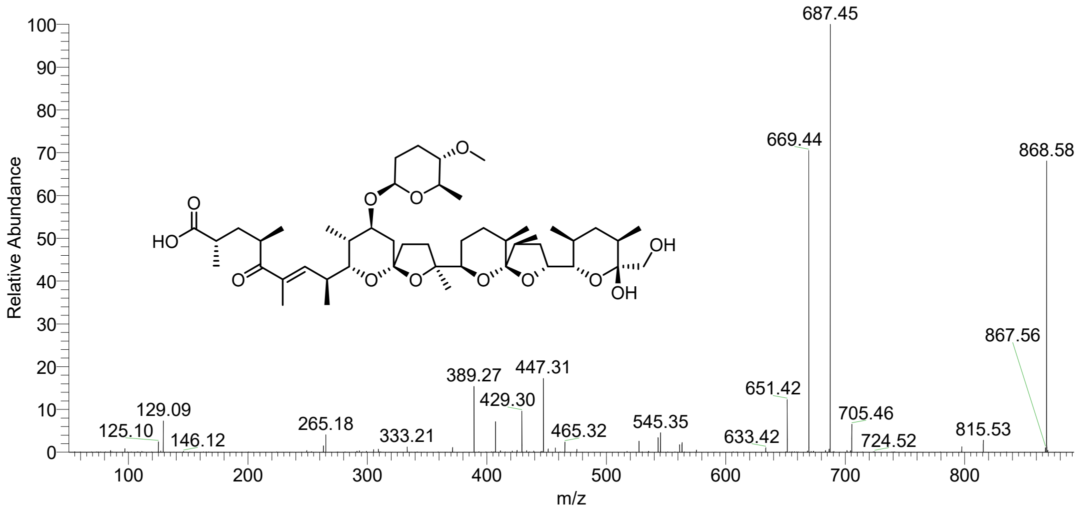

MS[2] fragmentation spectra of End-16.

Rudolph Research Analytical

This sample was measured on an Autopol VI, Serial #91058
Manufactured by Rudolph Research Analytical, Hackettstown, NJ, USA.

Measurement Date : Thursday, 25-AUG-2022

Set Temperature : OFF

Time Delay : Disabled

Delay between Measurement : Disabled

n	Average	Std.Dev.	% RSD	Maximum	Minimum
5	63.08	0.37	0.58	63.60	62.60

S.No	Sample ID	Time	Result	Scale	OR °Arc	WLG.nm	Lg.mm	Conc.g/100ml	Temp.
1	868	12:39:20 PM	63.60	SR	0.0636	589	100.00	0.100	26.7
2	868	12:39:29 PM	63.20	SR	0.0632	589	100.00	0.100	26.7
3	868	12:39:37 PM	63.10	SR	0.0631	589	100.00	0.100	26.7
4	868	12:39:45 PM	62.90	SR	0.0629	589	100.00	0.100	26.6
5	868	12:39:53 PM	62.60	SR	0.0626	589	100.00	0.100	26.6

Optical rotation value of End-16.

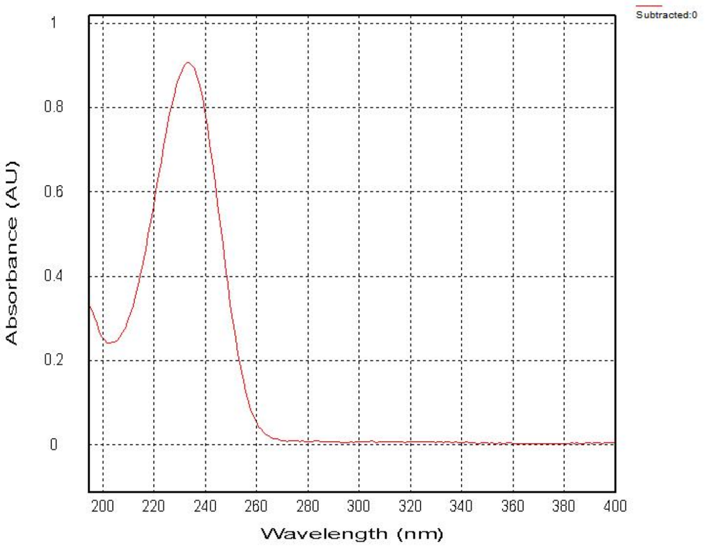

UV spectrum of End-16.

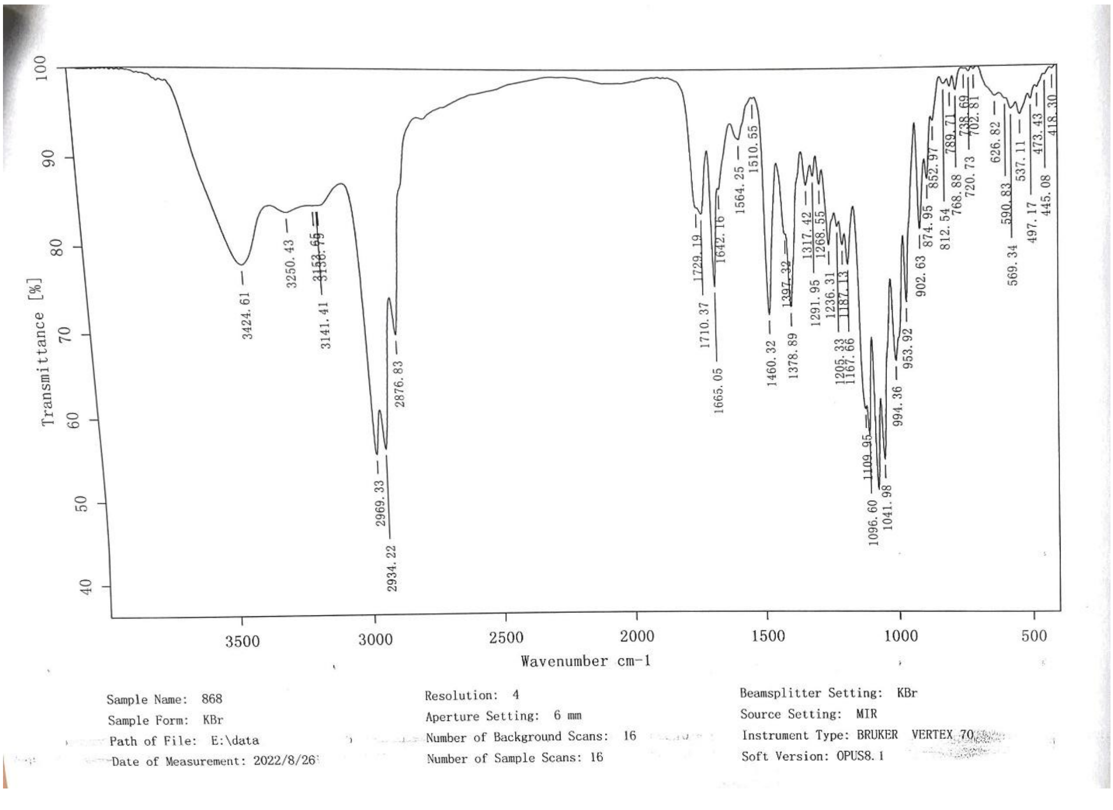

IR spectrum of End-16.

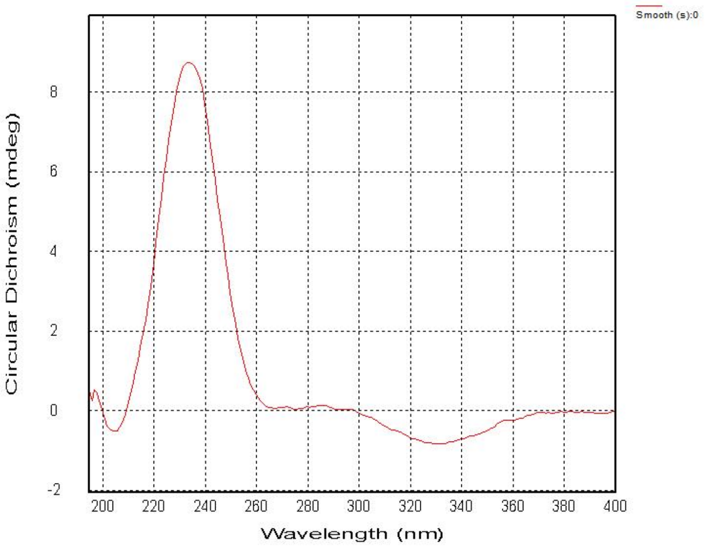

Experimental ECD spectra of End-16.

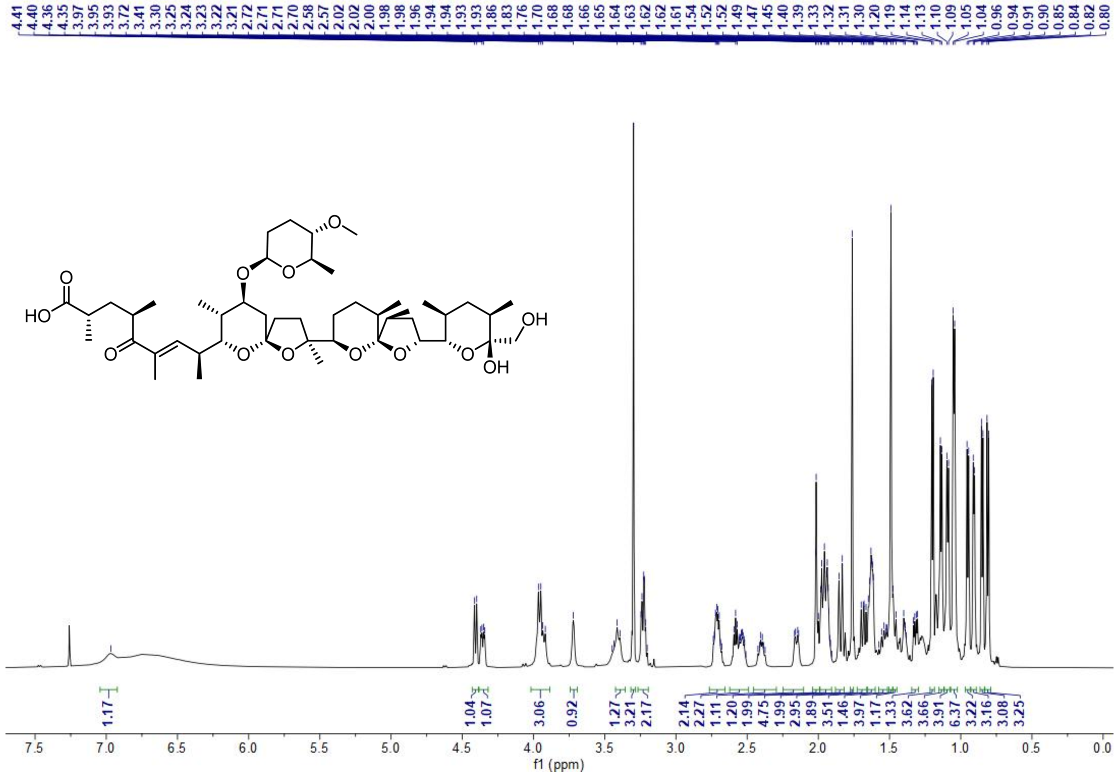

^{1}H NMR spectrum of End-16 in CDCl3.

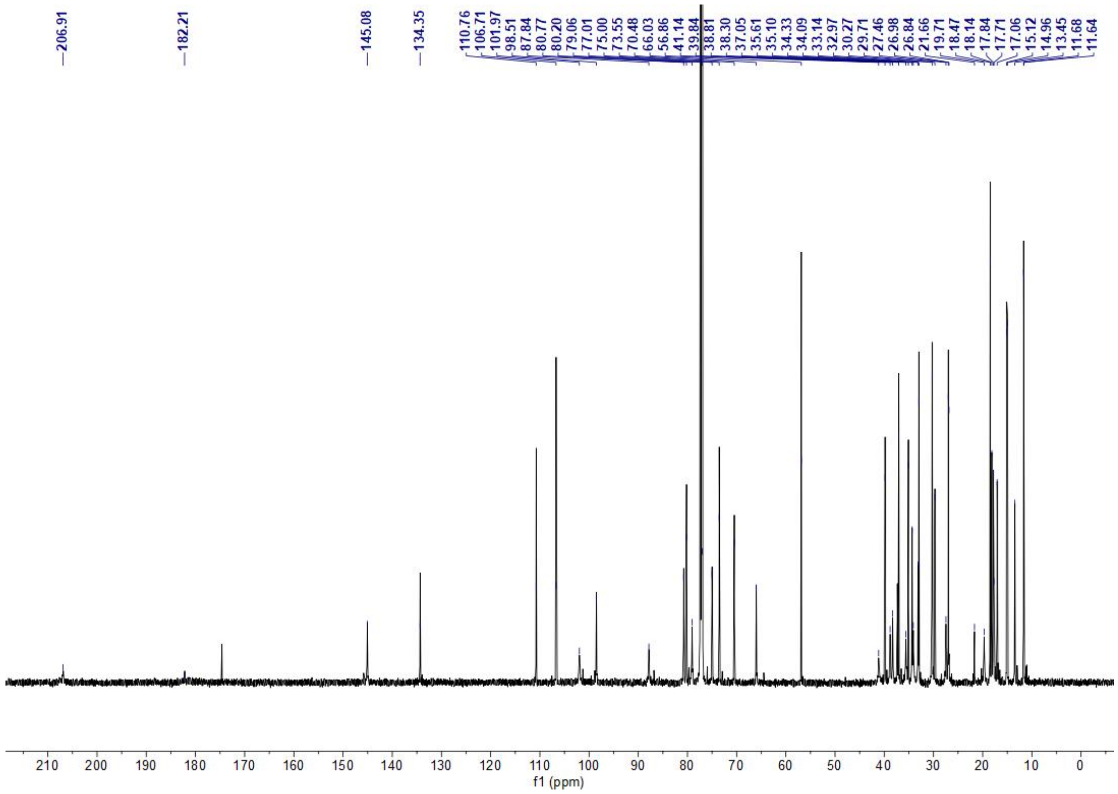

^{13}C NMR spectrum of End-16 in $CDCl_3$.

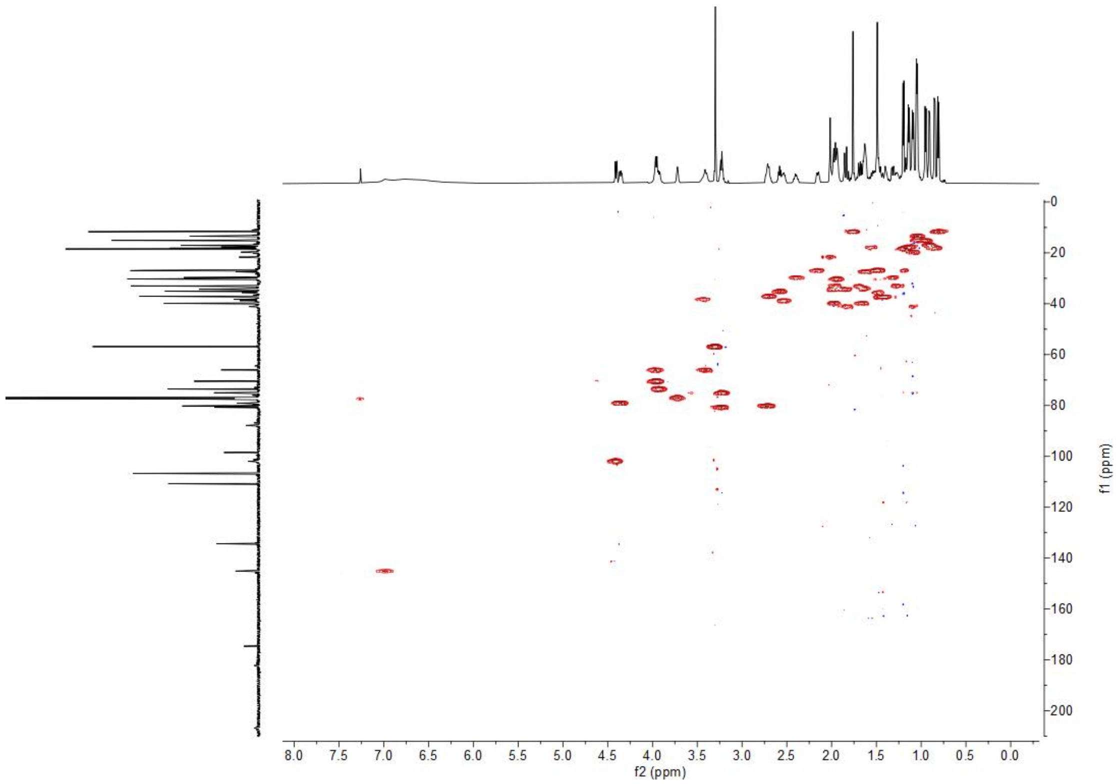

HSQC spectrum of End-16 in $CDCl_3$.

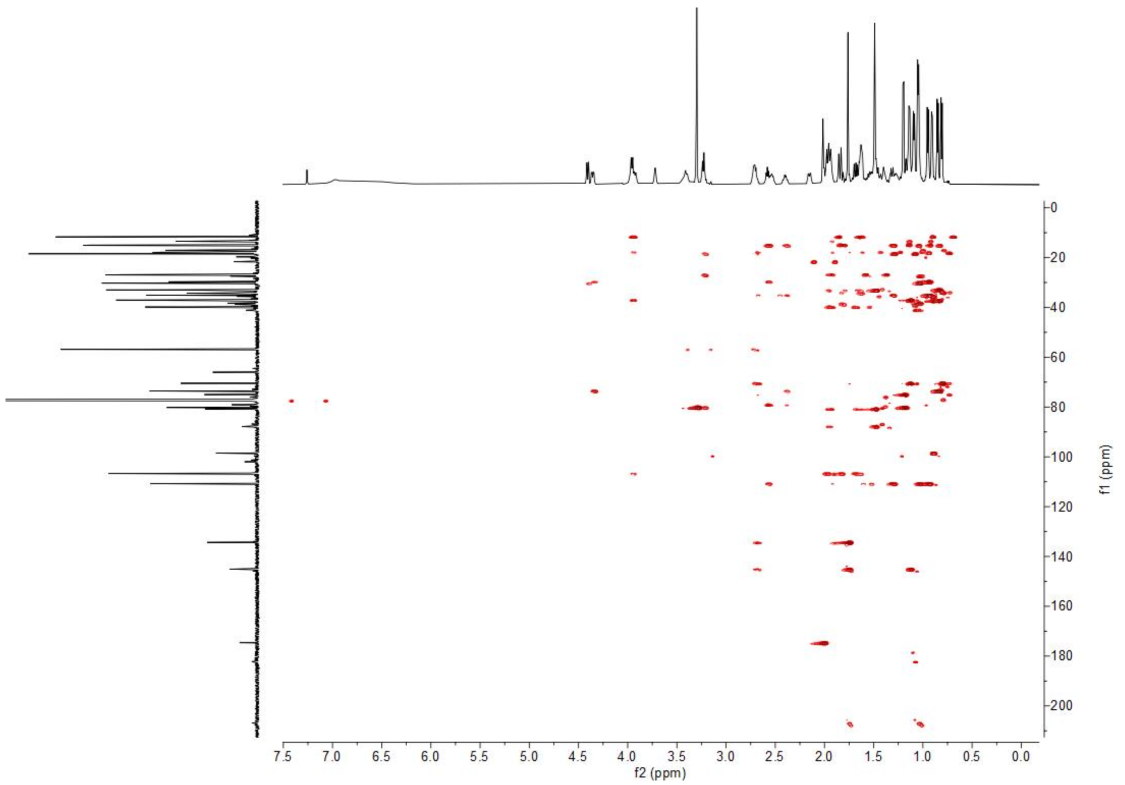

HMBC spectrum of End-16 in $CDCl_3$.

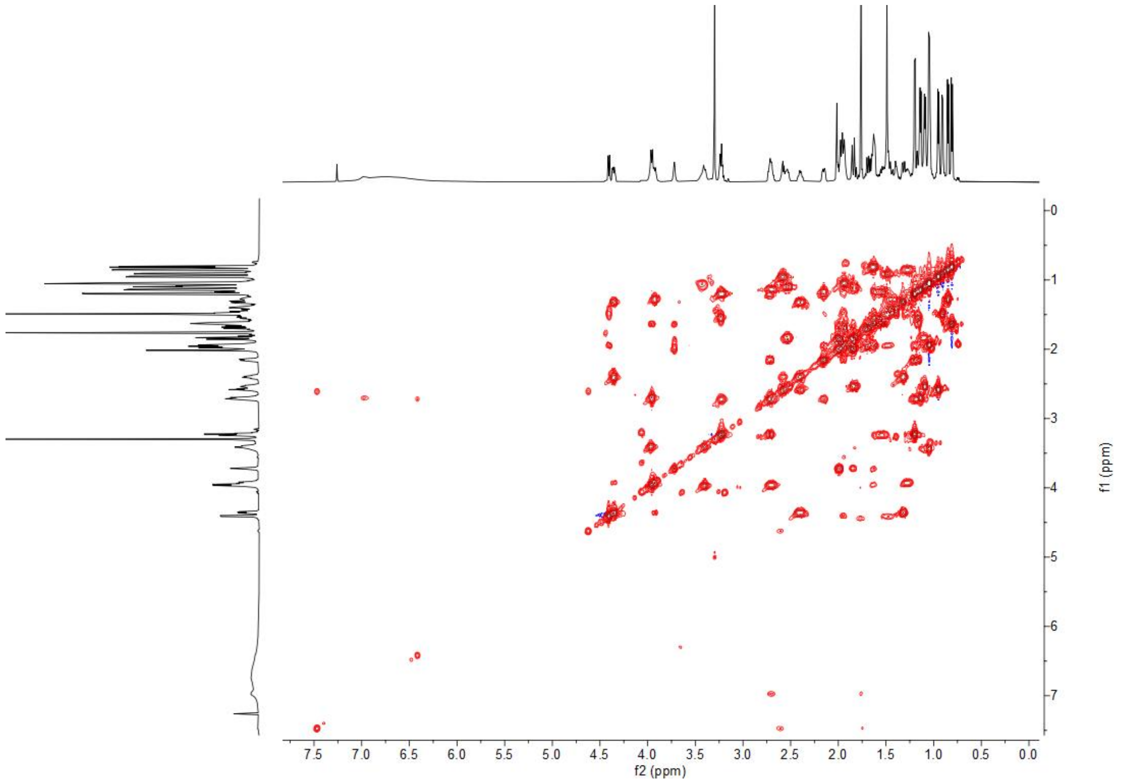

$^1H-^1H$ COSY spectrum of End-16 in $CDCl_3$.

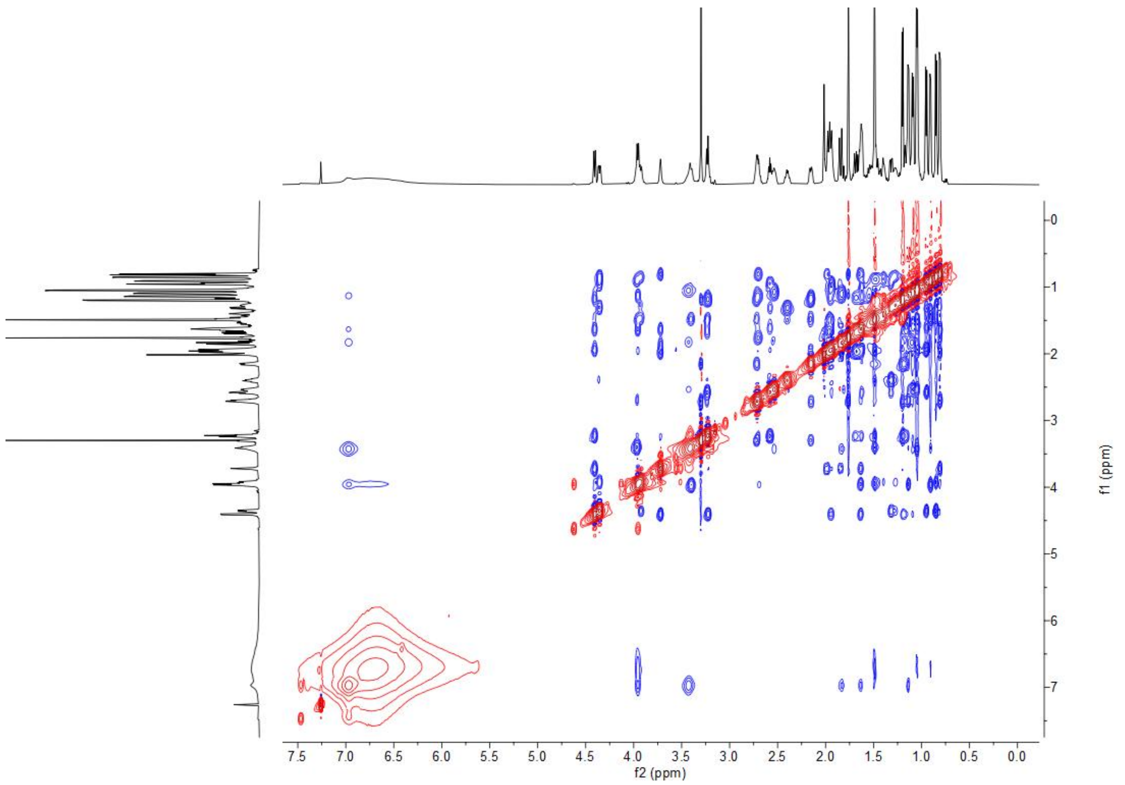

NOESY spectrum of End-16 in $CDCl_3$.

Supplementary References

[1] M. S. B. Paget, L. Chamberlin, A. Atrih, S. J. Foster, M. J. Buttner, *J. Bacteriol.* **1999**, *181*, 204-211.

[2] F. Flett, V. Mersinias, C. P. Smith, *FEMS Microbiol. Lett.* **1997**, *155*, 223-229.

[3] J. Fu, S. C. Wenzel, O. Perlova, J. Wang, F. Gross, Z. Tang, Y. Yin, A. F. Stewart, R. Müller, Y. Zhang, *Nucleic Acids Res.* **2008**, *36*, e113.

[4] K. F. Chater, L. C. Wilde, *J. Bacteriol.* **1976**, *128*, 644-650.

[5] Y. Sun, H. Hong, M. Samborskyy, T. Mironenko, P. F. Leadlay, S. F. Haydock, *Microbiology* **2006**, *152*, 3507-3515.

[6] C. Wilkinson, Z. Hughes-Thomas, C. Martin, I. Böhm, T. Mironenko, M. Deacon, M. Wheatcroft, G. Wirtz, J. Staunton, P. Leadlay, *J. Mol. Microbiol. Biotechnol.* **2002**, *4*, 417-426.

[7] H. Wang, X. Bian, L. Xia, X. Ding, R. Müller, Y. Zhang, J. Fu, A. F. Stewart, *Nucleic Acids Res.* **2014**, *42*, e37.

[8] H. Wang, Z. Li, R. Jia, Y. Hou, J. Yin, X. Bian, A. Li, R. Müller, A. F. Stewart, J. Fu, Y. Zhang, *Nat. Protoc.* **2016**, *11*, 1175-1190.

[9] M. Myronovskyi, E. Welle, V. Fedorenko, A. Luzhetskyy, *Appl. Environ. Microbiol.* **2011**, *77*, 5370-5383.

[10] Q. Liu, L. Xiao, Y. Zhou, K. Deng, G. Tan, Y. Han, X. Liu, Z. Deng, T. Liu, *Synth. Syst. Biotechnol.* **2016**, *1*, 207-214.
